# A Comprehensive Analysis of the Phylogeny, Genomic Organization and Expression of Immunoglobulin Light Chain Genes in *Alligator sinensis*, an Endangered Reptile Species

**DOI:** 10.1371/journal.pone.0147704

**Published:** 2016-02-22

**Authors:** Xifeng Wang, Gang Cheng, Yan Lu, Chenglin Zhang, Xiaobing Wu, Haitang Han, Yaofeng Zhao, Liming Ren

**Affiliations:** 1 State Key Laboratory of Agrobiotechnology, College of Biological Sciences, National Engineering Laboratory for Animal Breeding, China Agricultural University, Beijing, People’s Republic of China; 2 Beijing Zoo, Beijing 100044, People’s Republic of China; 3 College of Life Sciences, Anhui Normal University, Anhui Provincial Key Laboratory of the Conservation and Exploitation of Biological Resources, Wuhu 241000, People’s Republic of China; Chang Gung University, TAIWAN

## Abstract

Crocodilians are evolutionarily distinct reptiles that are distantly related to lizards and are thought to be the closest relatives of birds. Compared with birds and mammals, few studies have investigated the Ig light chain of crocodilians. Here, employing an *Alligator sinensis* genomic bacterial artificial chromosome (BAC) library and available genome data, we characterized the genomic organization of the *Alligator sinensis* IgL gene loci. The *Alligator sinensis* has two IgL isotypes, λ and κ, the same as *Anolis carolinensis*. The Igλ locus contains 6 C_λ_ genes, each preceded by a J_λ_ gene, and 86 potentially functional V_λ_ genes upstream of (J_λ_-C_λ_)_n_. The Igκ locus contains a single C_κ_ gene, 6 J_κ_s and 62 functional V_κ_s. All V_L_ genes are classified into a total of 31 families: 19 V_λ_ families and 12 V_κ_ families. Based on an analysis of the chromosomal location of the light chain genes among mammals, birds, lizards and frogs, the data further confirm that there are two IgL isotypes in the *Alligator sinensis*: Igλ and Igκ. By analyzing the cloned Igλ/κ cDNA, we identified a biased usage pattern of V families in the expressed V_λ_ and V_κ_. An analysis of the junctions of the recombined VJ revealed the presence of N and P nucleotides in both expressed λ and κ sequences. Phylogenetic analysis of the V genes revealed V families shared by mammals, birds, reptiles and *Xenopus*, suggesting that these conserved V families are orthologous and have been retained during the evolution of IgL. Our data suggest that the *Alligator sinensis* IgL gene repertoire is highly diverse and complex and provide insight into immunoglobulin gene evolution in vertebrates.

## Introduction

Immunoglobulin (Ig) is one of the most important primary effector molecules in the adaptive immune system of jawed vertebrates [1]. Each immunoglobulin is composed of a heavy (H) chain and one of two light (L) chain types: λ or κ in mammals. Each of these L chains typically covalently links to H by disulfide bonds formed by positionally conserved cysteine residues [2]. As exceptions, shark IgNAR and camelid IgGs are only composed of heavy chains [3, 4]. The Ig light chain is encoded by λ and κ loci, which differ significantly in their genomic organization. At the λ locus, multiple V_λ_ segments are followed by J_λ_-C_λ_ repeats. In contrast, the cluster of V_κ_ gene segments is followed by a cluster of J_κ_ gene segments and then by a single C_κ_ gene [5–7]. Lymphocytes can generate specific immunoglobulins against diverse antigens by a somatic recombination process, known as V (D) J recombination [8–10]. A pair of recombination signal sequences (RSSs) are composed of conserved heptamer and nonamer sequences and are separated by a relatively non-conserved spacer of either 12 or 23 bp, which is recognized by RAG1 and RAG2. Then, RAG introduces a double-strand break (DSB) between the RSS and the coding segments [11, 12]. Each of the L chains is the result of the imprecise and random combinatorial assembly of several gene fragments by a non-homologous end joining (NHEJ) pathway with the removal or addition of a random number of nucleotides [10, 13]. This imprecision in the coding joint arises from short additions of self-complementary (P) or random (N) nucleotides [9], small deletions, or a combination of these and contributes to the antigen receptor diversity generated by V (D) J joining [14].

IgL genes in cartilaginous fishes belong to four major groups: κ, λ, σ and σ-cart [13]. Among cartilaginous fish, the *Ginglymostoma cirratum* L chain genes have been studied most comprehensively. In a previous study, four L chain isotypes were identified in *Ginglymostoma cirratum*: type I (NS5), type II (NS3), type III (NS4) and type IV. The type III L chain is clearly κ, the type II light chain is somewhat more λ-like, the type I gene is closely related to but distinct from the σ gene [15–17] and is referred to as σ-cart, and type IV is homologous with the L chain isotype σ, found first in *Xenopus* and later in bony fish [13, 17]. The IgL isotypes currently found in teleost belong to κ (L1/G and L3/F), λ and σ(L2). These have been found in a cluster assemblage and, depending on the species, the number of IgL isotypes is different [17–26].

Three types of light chains have been identified in amphibians as well, based on studies of *Xenopus laevis*: ρ, σ and type III [17, 27–30]. Qin and colleagues completely characterized all three gene loci in *Xenopus tropicalis* [31] and supported the classification of amphibians in which the ρ gene belongs to the κ gene family and type III appears λ-like [17, 29]. Evolutionarily, mammals express two types of Ig light chain, λ and κ, which are expressed in varying ratios in different species [5, 32–36]. In *Mus musculus* serum, 95% of the light chains are κ and 5% are λ [5], whereas *Bos taurus* exhibit a biased usage pattern of λ chain [32]. Like *Homo sapiens*, *Sus scrofa* do not show any preference for the usage of the light chain [36]. Surprisingly, unlike reptiles and mammals, birds possess only one light chain, which is orthologous to the *Homo sapiens*/*Mus musculus* λ chain [37–41]. The genomic organization of the λ chain is similar to the heavy chain in birds: only one functional V_λ_ and J_λ_ are 1.8 kb apart and are located upstream from the C_λ_ gene in the *Gallus gallus* [42]. The light chain has evolved an exceptional mechanism of generating diversity due to multiple V_λ_ pseudogenes that modify the functional V_λ_ gene and can act as donors to form intrachromosomal gene conversion [43]. These results suggested that the typical birds IgL was likely already present in the common ancestor and remained unchanged over a long period of evolution [40].

Reptilia can be divided into two main evolutionary lineages: one gave rise to Squamata, while the other gave rise to Testudines, Crocodylia, and birds [44]. Some studies have been conducted to investigate Ig gene isotypes and their genomic organization in reptilia. Until now, IgM, IgD and IgY encoding genes have been identified in all Squamata species studied to date [45–47]. While it was shown that the *Anolis carolinensis* express two types of light chains: λ and κ [7, 39, 48], snakes lack the Igκ light chain isotype [45]. In the Testudine*s*, IgM, IgD, IgY and IgD2 encoding genes were described, and two immunoglobulin domains of IgD2 are shown to be homologous to bird IgA domains, suggesting that they may originate from a common ancestral gene [49–51]. Crocodilians appeared during the Middle Triassic, approximately 240 million years ago (MYA). Although similar in appearance, crocodilians, as reptiles, are only distantly related to lizards and are thought to be the closest relatives of birds and have thus occupied an important position in evolution [52, 53]. According to phylogenetic studies, crocodilians provide a phylogenetic link to other reptiles and birds, and analysis of their Ig genes may provide important clues to understanding Ig evolution. In addition, despite living in poor conditions, crocodilians are rarely subject to infections caused by bacteria and viruses because of their strong immune systems [54, 55]. However, there have been few studies on the crocodilian immune system. Recently, IgH genes of crocodilians were identified; the results indicated that there are multiple μ genes and that IgM subclasses can be expressed through class-switch recombination. The crocodilian α genes are the first IgA-encoding genes identified in reptiles and suggested that reptiles and birds share a common ancestral organization [56, 57].

Crocodilians are the closest phylogenetic group to birds, and they all come from a group known as archosaurs. However, little is known about the IgL locus of crocodilians. Although a previous study suggested that two distinct light chain types were present in alligator [48], the isotypes and the genomic organization of their encoding genes are still not known [39]. In this study, we present the phylogeny, genomic organization and expression of the Igλ/κ of the *Alligator sinensis* and provide insight into understanding the crocodilian immune system and the evolution of immunoglobulin in vertebrates.

## Materials and Methods

### Sample collection, DNA and RNA extract

Blood samples of *Alligator sinensis* were collected from the Beijing Zoo. Genomic DNA was extracted from the blood following the standard protocol. Total RNA was extracted from the blood using a TRIzol kit (TIANGEN BIOTECH, Beijing) following the manufacturer’s instructions. Our studies were approved by the Animal Care and Use Committee of the China Agricultural University.

### BAC library

An *Alligator sinensis* genomic BAC library was constructed using a service provided by Bioestablish Biotechnology Co., Ltd. (Beijing, China) and was stored in our laboratory [56].

### BAC screening and sequencing

Based on sequences derived from *Gallus gallus* and other related species, we designed degenerate primers for the Igκ/λ. We ascertained the identities of the PCR-generated product sequences by BLAST against the NCBI GenBank, and then designed specific primers for the Igκ/λ genes based on the determined sequences (S1 Table). BAC clones containing Igκ/λ genes were rescreened from the BAC library using PCR. The positive BAC clones were sequenced from both ends, and the end sequences were used to design primers to determine overlap ping BAC clones and to obtain the extended segments in the next round of screening (S1 Table). The positive BAC clones were then sequenced by shotgun sequencing and assembled with the next generation sequencing platform by BGI (Beijing, China).

### Cloning of expressed *Alligator sinensis* Igλ and Igκ light chain genes at the cDNA level

Expressed *Alligator sinensis* Igλ and Igκ chains were amplified using the 5’ RACE System kit (Invitrogen, Beijing). The gene-specific primers for the Igλ chain are as follows: IgLCL338L18, 5’-CAT TAG GGA GAT ACT ACA-3’; IgLCL303L21, 5’-CAG GGA TCC CAG CTC TCT ACT-3’; IgLCL219L21, 5’-AGG GTC TTC TCG ATG CTC TTC-3’; IgLCL129L21, 5’-GCT GGC CAT GTA CTT GTT GTC-3’. The sequences of these primers are conserved in the *Alligator sinensis* C_λ_ gene. Gene-specific primers for the Igκ chain are as follows: IgLCκ301L18, 5’-ATA AAG AAA GCA TAA GAA-3’; IgLCκ236L21, 5’-CGT ACA CTC GGT CCT CTT GAA-3’; IgLCκ121L21, 5’-CTG CTC TTG CTG TAC GTG TTG-3’, which are conserved in the *Alligator sinensis* C_κ_ gene.

All PCR amplifications were performed using a proofreading enzyme Pyrobest DNA polymerase (TaKaRa, Dalian). The PCR products were cloned into the pMD-19 T vector (TaKaRa, Dalian) and sequenced.

### Southern blotting

Genomic DNA was digested with restriction endonuclease and was loaded into a 0.9% agarose gel, electrophoresed for 6 h, and transferred to a positively charged nylon membrane (Roche, Germany) for hybridization. The restriction endonucleases *Bgl* II, *Nco* I, *Hind* III and *Sph* I were used to digest genomic DNA to identify Igλ. Genomic DNA was digested with restriction endonucleases *Kpn* I, *Nde* I and *Xba* I to validate Igκ. The single exon of the C_λ_/C_κ_ probe was labeled using a PCR digoxigenin probe synthesis kit (Roche, Germany). The primers used to amplify the C_λ_/C_κ_ exon probes were as follows: LC-F, 5’-ACA GCC AAA GGC CTC TCC T-3’; LC-R, 5’-CGA TCT CTT CAG GGT CTT CTC-3’; KC-F, 5’-AAA GGG GGA AGA GCC ACC-3’; KC-R, 5’-TAC ACT CGG TCC TCT TGA-3’. The hybridization and detection were performed following the manufacturer’s instructions.

### Construction of phylogenetic trees

The phylogenetic trees were constructed using MrBayes3.1.2 [58] and were viewed in TREEVIEW [59]. Furthermore, in order to validate the topologies of the phylogenetic trees, we also used MEGA6.0 and Phylip3.695 [60] to build all the phylogenetic trees [59]. Multiple amino-acid alignments for the tree construction were performed using *ClustalW*. Each V_λ_/_κ_ subgroup was represented with one family per species chosen at random. The accession numbers of sequences used for variable regions are as follows: *Heterodontus francisci* σ (ABO64185); *Heterodontus francisci* type I (CAA33375); *Heterodontus francisci* type II (AAA59379); *Heterodontus francisci* type III (AAA59373); *Ginglymostoma cirratum* NS5 (AAV34678); *Ginglymostoma cirratum* σ (ABO64187); *Danio rerio* type I (AAG31721); *Danio rerio* type II (AAG31729); *Danio rerio* type III (AAG31698); *X*. *laevis* type III V1 (AAL40100); *X*. *laevis* type III V2 (AAL40101); *X*. *laevis* type III V3 (AAL40102); *X*. *laevis* type III V4 (AAL40103); *X*. *laevis* type III V5 (AAL40097); *X*. *laevis* type III V6 (AAL40093); *X*. *laevis* σ (NP_001087883); *X*. *laevis* ρ (AAH68859); *Gallus gallus* IGλV (BAB71862); *Anas platyrhynchos* IGλV (AAA03006); *Anolis carolinensis* IGκV (ACB45832); *Anolis carolinensis* IGλV1 (XP_008115579); *Anolis carolinensis* IGλV2 (XP_008115579); *Anolis carolinensis* IGλV3 (XP_008115579); *Anolis carolinensis* IGλV4 (XP_008115579); *Anolis carolinensis* IGλV5 (XP_008115579); *Mus musculus* IGκV1-132 (CAB46115); *Mus musculus* IGκV2-112 (AAA39032); *Mus musculus* IGκV3-4 (CAA75909); *Mus musculus* IGκV4-61 (CAB46123); *Mus musculus* IGκV5-45 (CAB46329); *Mus musculus* IGκV6-25 (CAB46320); *Mus musculus* IGκV7-33 (AAC04340); *Mus musculus* IGκV8-30 (CAB46308); *Mus musculus* IGκV9-120 (CAA24186); *Mus musculus* IGκV10-95 (AAC14726); *Mus musculus* IGκV11-125 (CAB51813); *Mus musculus* IGκV12-38 (CAB46311); *Mus musculus* IGκV13-84 (CAB46176); *Mus musculus* IGκV14-130 (CAB46155); *Mus musculus* IGκV15-103 (CAB46175); *Mus musculus* IGκV16-104 (CAB46298); *Mus musculus* IGκV17-121 (CAB46168); *Mus musculus* IGκV18-36 (CAB46323); *Mus musculus* IGκV19-93 (CAB46297); *Mus musculus* IGλV1 (AAA39165); *Mus musculus* IGλV3 (AAA39169); *Mus musculus* IGλV4 (AAA39434). All other sequences were derived in this study. The accession numbers of sequences used for constant regions are as follows: *Gallus gallus* λ (AAA48862); *Anas platyrhynchos* λ (AAA03009); *Ornithorhynchus* λ (AAO16062); *Homo sapiens* λ (AAA59107); *Mus musculus* λ (AAA39089); *Bos taurus*λ (AAI46273); *Didelphimorphia* λ (AAL37214); *Oryctolagus cuniculus* λ (AAA31360); *Anolis carolinensis* λ (XP_008115579); *Sus scrofa* λ (AAA03572); *Chelonia mydas* λ (XP_007055069.1); *Chrysemys pictabellii* λ (XM_008167097.1); *X*. *laevis* type III (AAL40101); *X*. *tropicalis* type III (AAI66944); *Mus musculus* κ (CAA24185); *Homo sapiens* κ (AAY24201); *Bos taurus* κ (AAI51501); *X*. *laevis* ρ (AAA49880); *X*. *laevis* σ (NP_001087883.1|); *X*. *tropicalis* σ (AAI67133); *X*. *tropicalis* ρ (AAI58339); *Oryctolagus cuniculus* κ (CAA10920); *Sus scrofa* κ (AHB17990); *Didelphimorphia* κ (AAL17618); *Ornithorhynchus* κ (AAO84649); *Chelonia mydas* κ (EMP6807.1); *Chrysemys pictabellii* κ (XM_008169724.1); *Ginglymostoma cirratum* NS5 (AAV34681); *Ginglymostoma cirratum* NS4 (A49633); *Ginglymostoma cirratum* NS3 (Ref.[61]); *Ginglymostoma cirratum* σ (ABO64188); *Danio rerio* IGIC1 (AAG31721); *Danio rerio* IGIC2 (XP_009298120); *Heterodontus francisci* type I (CAA33376); *Heterodontus francisci* type II (CAA33375); *Heterodontus francisci*type III (AAA59373); *Heterodontus francisci* σ (ABO64185); *Salmo salar* IGIC1 (AAG18364); *Salmo salar* IGIC2 (AAG37201); *Salmo salar* IGIC3 (AAK97642). All other sequences were derived in this study.

### Sequence computations

DNA and protein sequence editing, alignments and comparisons were performed with the MegAlign software (DNASTAR). The EquCab2 assembly in Ensembl database (http://www.ensembl.org/index.html) was used to retrieve the genomic contig that contained the *Alligator sinensis* Igλ/Igκ chain sequences. IgBLAST (http://www.ncbi.nlm.nih.gov/igblast/) was used to predict the V_λ_/_κ_ segments. Germline V_λ_ and V_κ_ gene segments were grouped into families using the IMGT numbering system [62]. The RSSs for the V and J gene segments were analyzed using the online program FUZZNUC (http://embossgui.sourceforge.net/demo/fuzznuc.html).

## Results

### Genomic organization of IgL chain gene loci in *Alligator sinensis*

**Fig 1:**
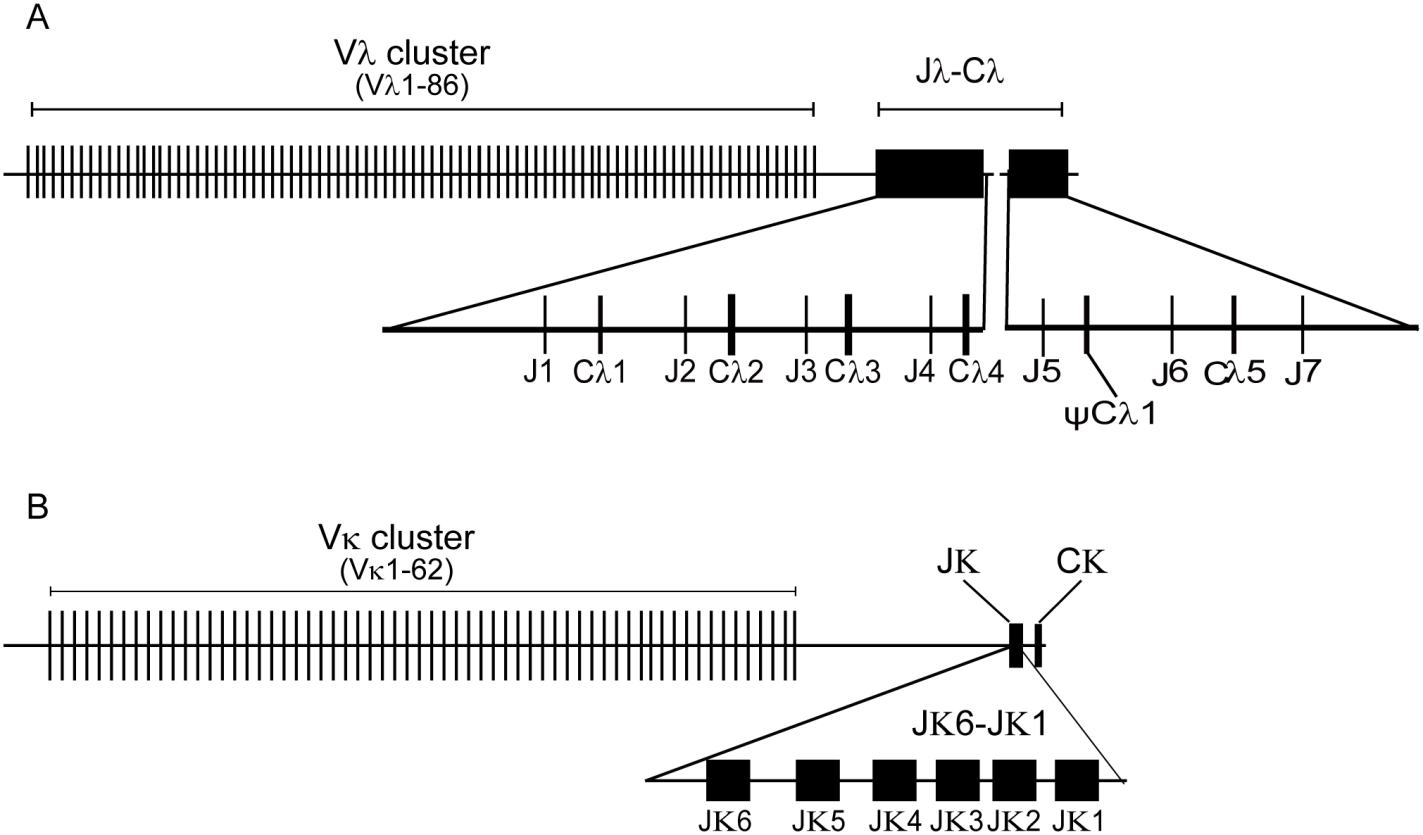
Schematic map of the *Alligator sinensis* immunoglobulin light chain gene loci. (A) Schematic map of the *Alligator sinensis* immunoglobulin light chain λ gene loci. (B) Schematic map of the *Alligator sinensis* immunoglobulin light chain κ gene loci. V: variable gene segments; J: joining gene segments; C: constant region gene; pseudo-variable gene segments were not be shown in the figure.

**Fig 2:**
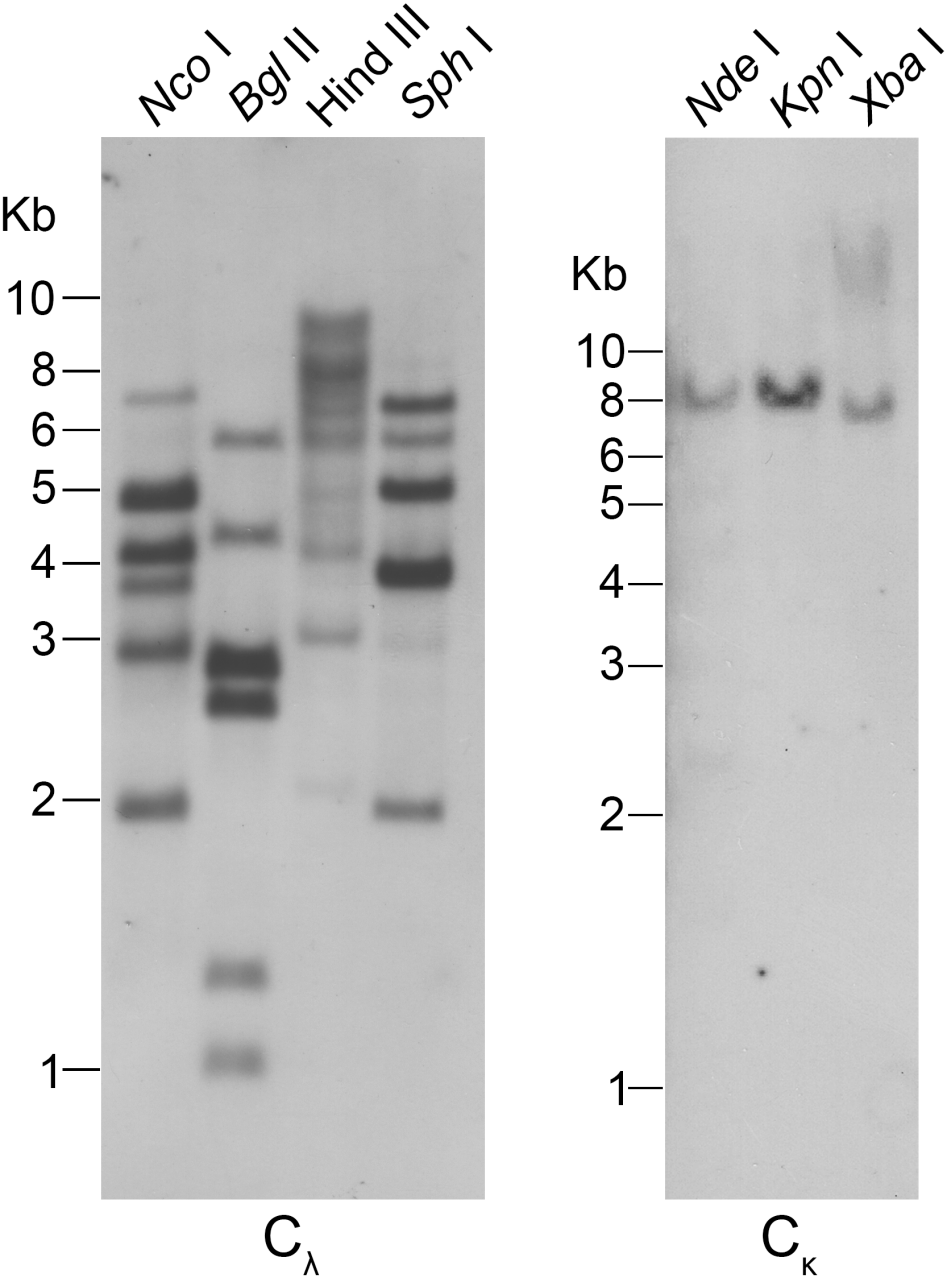
Southern blotting detection of the *Alligator sinensis* Ig light chain C gene segments. Genomic DNA was digested with restriction endonucleases, which are indicated above each lane, and hybridized with probes for C_λ_ and C_κ_, respectively.

According to the IgL chain isotypes in *Anolis carolinensis*, the genomic organization of the Igλ gene locus in the *Alligator sinensis* was analyzed. An *Alligator sinensis* BAC (bacterial artificial chromosome) genomic library, which was constructed using the peripheral blood leucocytes from an *Alligator sinensis* and stored in our laboratory, was employed. The library is composed of 2.1 × 10^5^ clones with an average insert size of ~100 kb, representing ~9 × genomic coverage (*Alligator sinensis* genome size of ~2.5 Gb). Using a PCR-based approach and sequencing, we identified four Igλ gene-positive BAC clones (Y210O3, Y47P24, Y147P18 and Y127H24) (S1 Table). An ~331 Kb genomic sequence was obtained and was found to contain four λ chain C genes (C_λ_1, C_λ_2, C_λ_3 and C_λ_4) and four λchain J genes (J_λ_1, J_λ_2, J_λ_3 and J_λ_4) in front of each C gene, spanning approximately 12 kb DNA, there are potentially 37 functional λ chain V genes, 32 λ chain V pseudogenes and one ORF. Furthermore, using the available genomic database of the *Alligator sinensis* (http://www.ncbi.nlm.nih.gov/), a genomic contig (AVPB01119656.1) was identified by BLAST; three λ chain C genes were identified in the contig: one is identical with C_λ_4 in the ~331 kb genomic sequence, and one appears to be a pseudogene because it contains an in-frame stop codon. Furthermore, three J genes were found in the contig. There are six λ chain C genes (C_λ_1, C_λ_2, C_λ_3, C_λ_4, ΨC_λ_1 and C_λ_5) and seven λ chain J genes (J_λ_1, J_λ_2, J_λ_3, J_λ_4, J_λ_5, J_λ_6 and J_λ_7) (Fig 1 and S1 Fig). All of the C_λ_ genes share at least 84.1% amino acid sequence identity, of which the amino acid sequence identities between C_λ_1 and C_λ_2, C_λ_1 and C_λ_4, C_λ_2 and C_λ_3, C_λ_2 and C_λ_4, and C_λ_3 and C_λ_4 are greater than 90.7%. Each C_λ_ gene was preceded by a single J gene segment that was 5' flanked by conserved RSS (nonamer and heptamer) with a 12 bp nucleotide spacer, resembling the genomic organization of the λ chain gene loci in mammals (S2A Fig). However, a single J segment (J_λ_7) was found downstream from C_λ_5, but no additional λ chain C genes were identified (Fig 1A and S1 Fig), which implied that there are more C_λ_ genes in the *Alligator sinensis* Igλ locus. A protein sequence alignment of the identified C genes with the C_λ_ in lizards, birds and mammals uncovered an identical pattern with regard to the cysteine distribution (S2B Fig). Genomic Southern blotting with the C_λ_ exon as a probe was conducted to verify the numbers of λ chain C genes. In *Bgl* II, *Nco* I and *Sph* I digested DNA, different shades of six bands were detected, and there were more than six bands in *Hind* III digested DNA, which indicated that there are additional C_λ_ genes in the chromosome (Fig 2).

**Fig 3:**
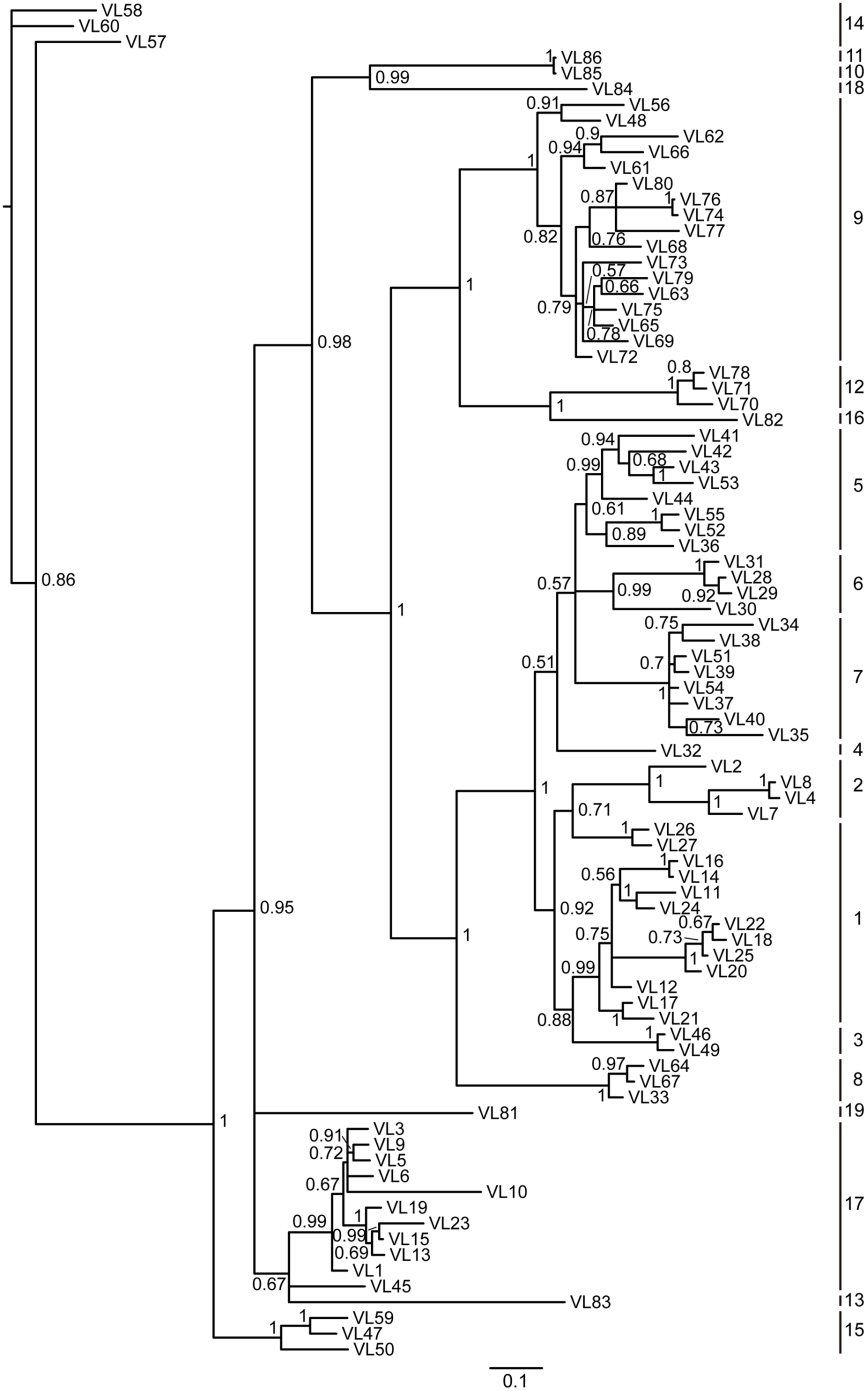
Phylogenetic tree analysis of the 86 *Alligator sinensis* V_λ_ genes. A phylogenetic tree of the nucleotides of 86 *Alligator sinensis* V_λ_ segments was constructed. The 19 V_λ_ gene families are labeled with numbers on the right. Phylogenetic trees were constructed using MrBayes3.1.2 [58] and viewed in TREEVIEW [59].

We performed a BLAST search against the *Alligator sinensis* whole-genome shotgun sequence (WGS) assembly deposited in the Ensemble database. Seven genomic contigs (KE698600.1, AVPB01102472.1, KE698001.1, KE698031.1, KE697531.1, KE697626.1 and KE695978.1) were found to contain λ chain V gene segments (S2 Table). Each V_λ_ gene, which is 3' flanked by a conserved RSS (heptamer and nonamer) with 23 bp nucleotide spacer, was identified, resembling the genomic organization of the V_λ_ chain gene loci in mammals. In summary, a total of 86 potentially functional V_λ_ segments (Fig 1B and S1 Appendix), two ORFs and 67 V_λ_ pseudogenes were identified upstream from the (J_λ_-C_λ_)_n_ segments (S1 Fig), and 67 V_λ_ that either contain in-frame stop codons or lack a leading peptide appear to be pseudogenes (S2 Appendix). According to the sequence identity (> 75% sequence identity within a single family) and phylogenetic analysis, the potentially functional V_λ_ genes can be classified into at least 19 families (Fig 3; S3 and S4 Figs; S3 Appendix). In addition, there may be more V_λ_ segments unidentified in the *Alligator sinensis* based on the gaps in the contig and incomplete genomic data.

A similar approach was used to identify the C_κ_ from the genome of the *Alligator sinensis*. Using a PCR-based approach and sequencing, we obtained 5 Igκ gene-positive BAC clones (Y146M9, Y65C14, Y77E6, Y329F14 and Y146B4) (S1 Table). An ~484 kb genomic sequence was found to contain a single copy of the *Alligator sinensis* C_κ_ gene, which showed homology to several mammalian species, six J_κ_ gene segments and 66 V_κ_ gene segments, including 29 V_κ_ pseudogenes. We performed a BLAST search against the *Alligator sinensis* whole-genome shotgun sequence (WGS) assembly deposited in the Ensemble database. Seventeen DNA contigs (AVPB01013186.1, AVPB01053098.1, AVPB01130521.1, AVPB01143799.1, KE695928.1, KE697554.1, KE697644.1, KE698008.1, KE698055.1, KE698081.1, KE698096.1, KE698098.1, KE698149.1, KE698335.1, KE698356.1, KE698428.1, and KE698585.1) comprise a leash of V_κ_ genes that is variable in number from 1 to 15 (S3 Table). At least 62 potentially functional V_κ_ gene segments (Fig 1B and S4 Appendix); 56 V_κ_ pseudogenes, which either contain in-frame stop codons or lack a leading peptide (S5 Appendix); and 4 partial V_κ_ genes were identified from the *Alligator sinensis* genomic sequence (S5 Fig).

The C_κ_ gene as a single copy in the genome was subjected to confirmation by Southern blotting. We designed a pair of degenerate primers for the C_κ_ gene based on the conserved C_κ_ sequences of the *Alligator sinensis*. Only a single band was observed in *Kpn* I, *Nde* I and *Xba* I digested genomic DNA, which supported the C_κ_ gene as a single copy present in the genome (Fig 2). Upstream of the single copy of the C_κ_ gene, six functional J_κ_s (J_κ_1-J_κ_6) gene segments with RSS interrupted by a 23 bp nucleotide spacer at their 5’ ends were identified (S6A Fig). An amino acid sequence alignment of the C_κ_ gene in the *Alligator sinensis* with other species suggested homology to the Igκ chains of several vertebrates, including the *Homo sapiens*, *Mus musculus*, *Didelphimorphia*, *Ornithorhynchus*, *Anolis carolinensis*, *X*. *laevis* and *X*. *tropicalis* (S6B Fig). The C_κ_ protein sequence contained three cysteines, among which the third one at the carboxyl terminal was assumed to link heavy chains (S6B Fig).

**Fig 4:**
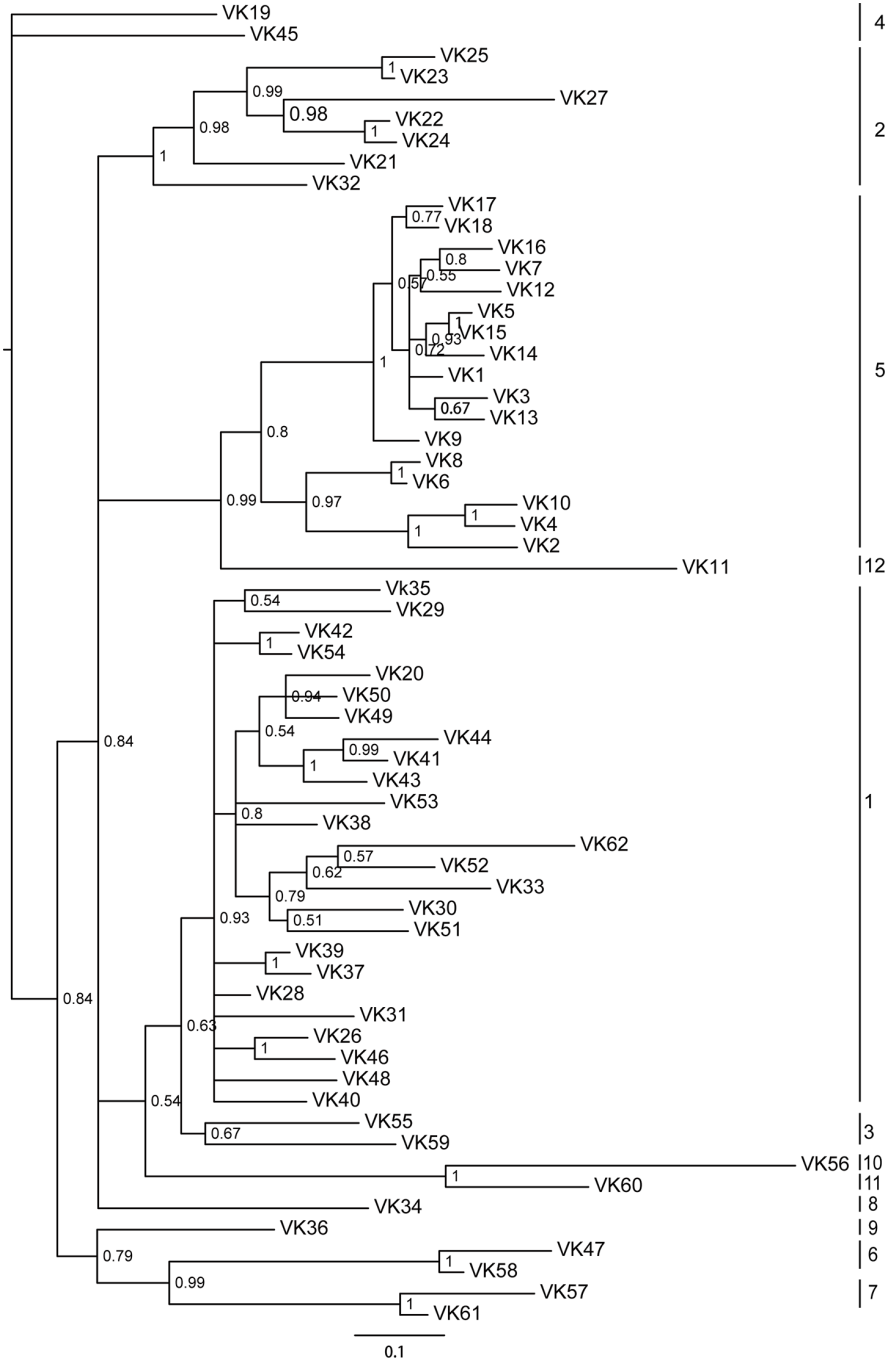
Phylogenetic tree analysis of the 62 *Alligator sinensis* V_κ_ genes. A phylogenetic tree of the nucleotides of the *Alligator sinensis* V_κ_ segments was constructed. The 12 V_κ_ gene families are labeled with numbers on the right. Phylogenetic trees were constructed using MrBayes3.1.2 [58] and viewed in TREEVIEW [59].

Almost all V_κ_ genes were flanked on the 3’ end by RSS and were separated by a 12 bp nucleotide spacer to conform the 12–23 rules (S5 Appendix). All V_κ_ genes showed the same transcriptional orientation as (J_κ_)_n_-C_κ_, with the exception of pseudogene V_κ_46. The 62 potentially functional V_κ_ genes can be integrated into 12 families based on the phylogenetic analysis and the rule that V_κ_ members in one family share at least 75% identity at the nucleotide level (Fig 4; S7 and S8 Figs; S6 Appendix). Because gaps exist in the contigs and the genomic data are incomplete, it is possible that more V_κ_ genes present in the *Alligator sinensis* genome were not found.

### Phylogenetic analysis of the *Alligator sinensis* Ig light chain gene segments

**Fig 5:**
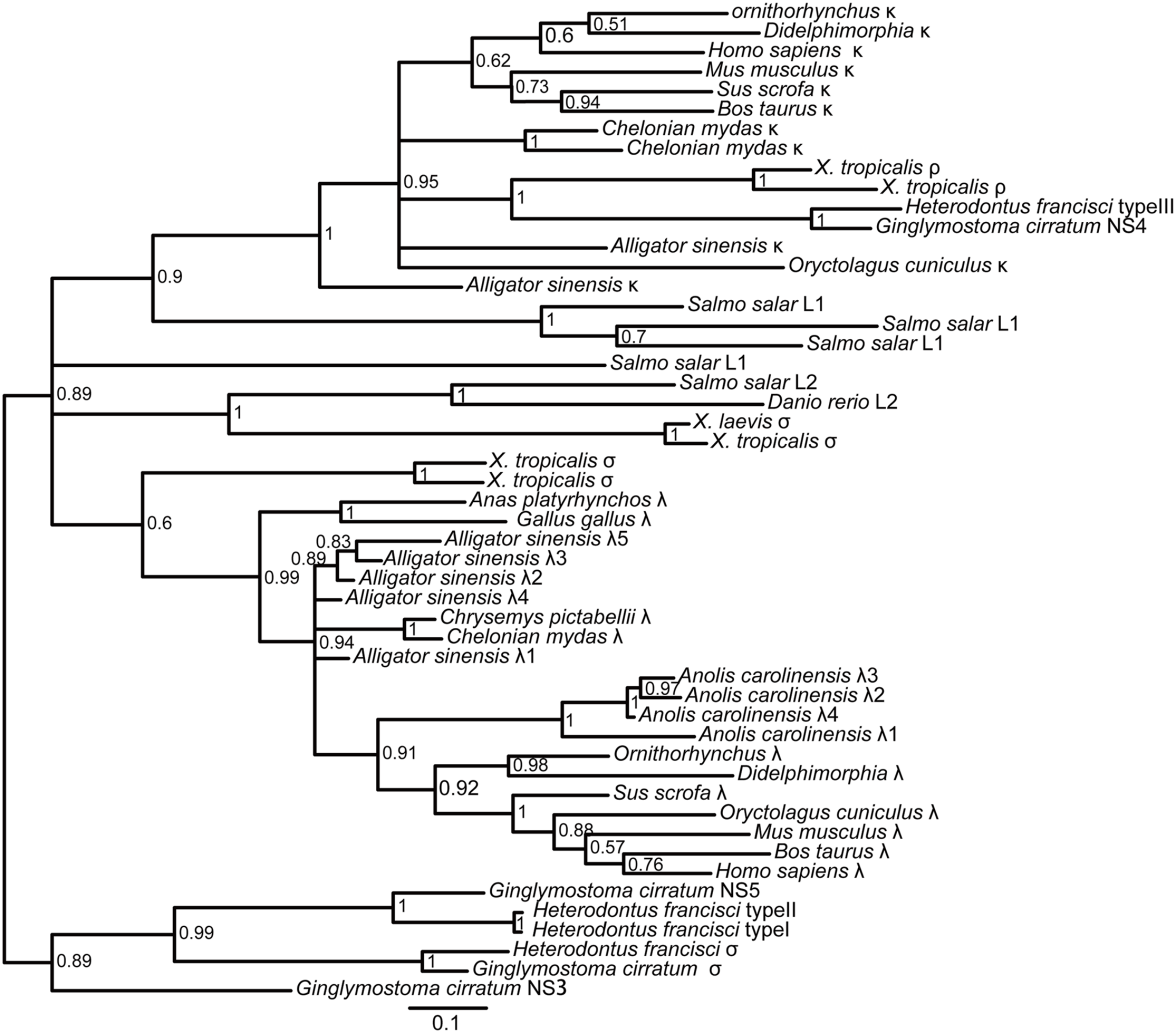
Phylogenetic analysis of the IgL chain C genes in jawed vertebrates. The phylogenetic tree was constructed using C domains. The scale shown as a bar represents the genetic distance (number of nucleotide changes at the given scale). The credibility value for each node is shown. Phylogenetic trees were constructed using MrBayes3.1.2 [58] and viewed in TREEVIEW [59].

**Fig 6:**
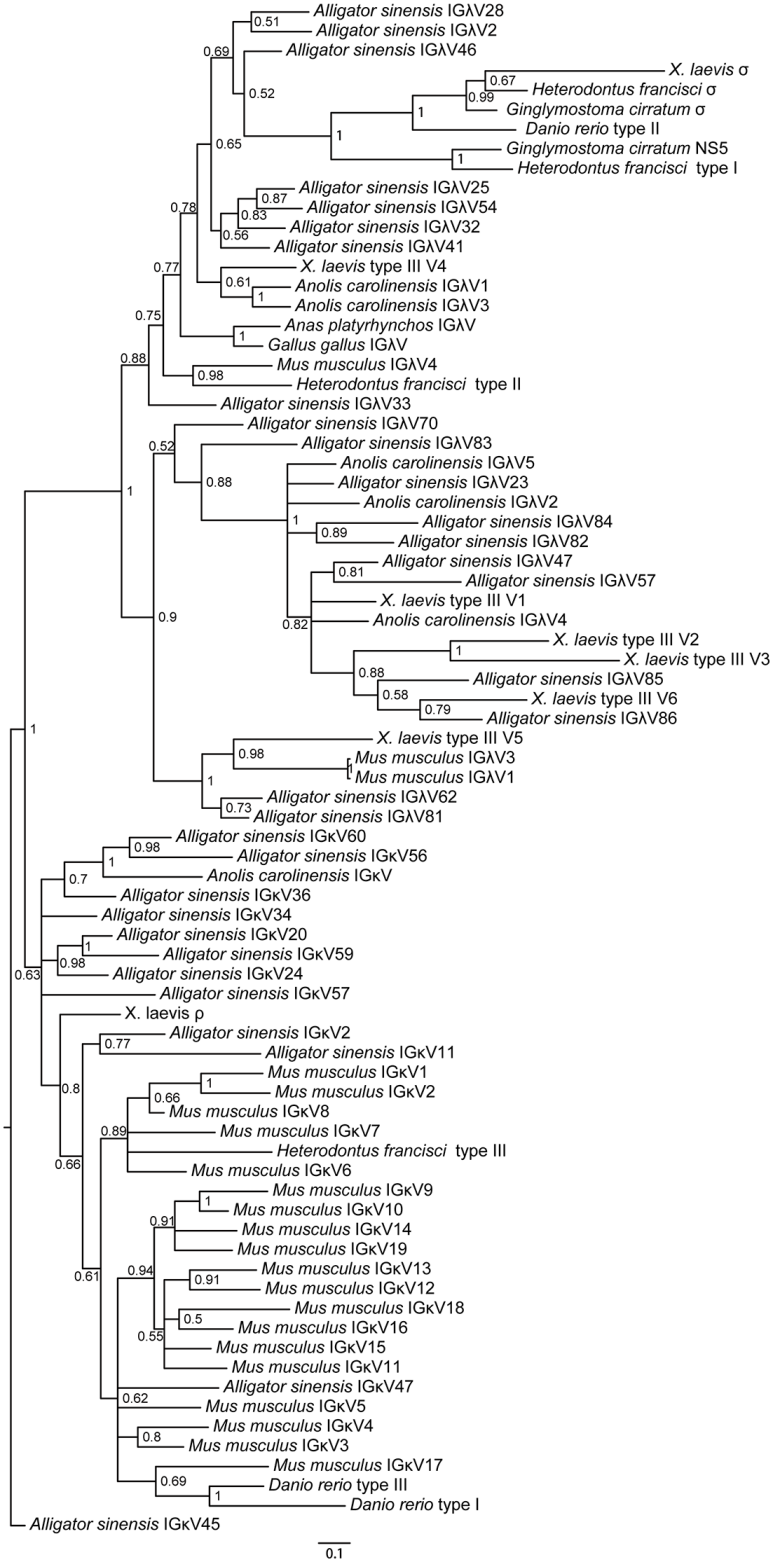
Phylogenetic analysis of the IgL chain V genes in jawed vertebrates. The phylogenetic tree was constructed using V domains. The scale shown as a bar represents the genetic distance (number of nucleotide changes at the given scale). The credibility value for each node is shown. Phylogenetic trees were constructed using MrBayes3.1.2 [58] and viewed in TREEVIEW [59].

Using the amino acid sequences of *IGLV-* and *IGLC*- encoded genes from different jawed vertebrates, we constructed V and C phylogenetic trees, respectively. The trees were constructed using protein sequences without CDR3. The phylogenetic trees, based on both the C domains and the V domains, support the fact that there are three major groups of IgL genes in jawed vertebrates: κ, λ and σ (including σ-cart), and *Alligator sinensis* κ and λ clearly fall into their own respective groups, suggesting that the *Alligator sinensis* has only two IgL isotypes: κ and λ (Figs 5 and 6; S9, S10, S11 and S12 Figs). The results reveal that the ρ gene of *X*. *tropicalis*, teleost L1 and L3 and cartilaginous fish type III/NS4 is located in the κ group, which also includes the κ genes of the Crocodilians, lizards and mammals. Teleost L2, cartilaginous fish type II/NS3, and *X*. *tropicalis* type III all belong to λ groups, including the λ genes of the Crocodilians, lizards, birds and mammals. The σ genes are only found in cartilaginous fish, teleost and amphibians. Taken together with our shared synteny of the κ and λ locus in the *Alligator sinensis* and the phylogenetic analysis, these data provide convincing evidence that the *Alligator sinensis* expresses two IgL isotypes: κ and λ. From the phylogenetic analysis, it is not difficult to obtain the relationships between the *Alligator sinensis* and other species’ V families. *Alligator sinensis* families V_κ_10 and V_κ_11 are clustered with *Anolis carolinensis* V_κ_; *Alligator sinensis* family V_κ_7 is clustered with *X*. *laevis* ρ; and the same phylogenetic analysis was also performed for V_λ_. As shown in Fig 6, *Alligator sinensis* families V_λ_9 and V_λ_19 are clustered with *X*. *laevis* type III V5 and *Mus musculus* families 1 and 3; *Alligator sinensis* families V_λ_1-V_λ_8 are related to the *Anolis carolinensis* V_λ_1, V_λ_3, *Gallus gallus* and *Anas platyrhynchos* V_λ_ and *X*. *laevis* type III V4; and *Alligator sinensis* families V_λ_11 is clustered with *X*. *laevis* type III V6. The V genes were orthologous in different isotypes of IgL. We found no relations between the remaining V_λ_ genes and other jawed vertebrate species, suggesting that V_λ_ genes exhibit more abundant diversity in the *Alligator sinensis*.

### Syntenic analysis of Igλ and Igκ chain loci in tetrapods

**Fig 7:**
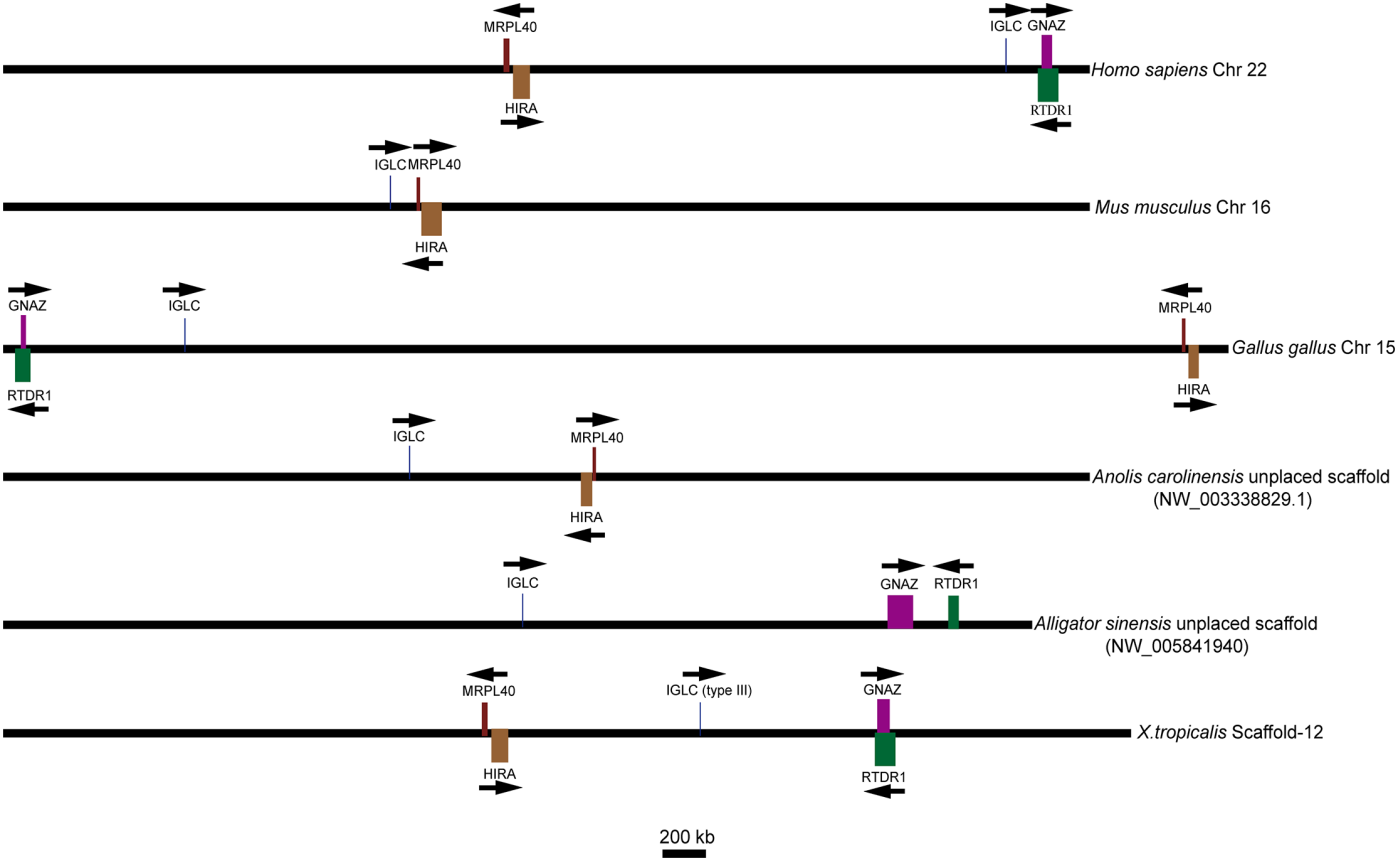
Chromosomal locations of the λ genes in different species and type III genes in *X*. *tropicalis*. Arrows indicate the transcriptional orientation of the genes. Chr: chromosome; IGLC: immunoglobulin λ chain constant region gene; GNAZ: guanine nucleotide-binding protein, α z subunit; HIRA: histone cell cycle regulation defective homologue A; MRPL40: mitochondrial ribosomal protein L40; RTDR1: rhabdoid tumor deletion region gene 1. The figure was modified from Ref. [31].

To determine the identified genes belonging to the λ lineage, we analyzed the chromosomal location relative to the flanking genes of the available genomic data containing the Igλ loci in tetrapods. *GNZA* (guanine nucleotide-binding protein, α z subunit) and *RTDR1* (rhabdoid tumor deletion region gene 1), *MRPL40* (mitochondrial ribosomal protein L40) and *HIRA* (histone cell cycle regulation defective homologue A) located on, respectively, the two sides of the λ locus in *Homo sapiens* were selected as markers to provide evidence for the gene. An available genomic contig (NW_005841940) containing the Igλ locus of the *Alligator sinensis* was used for analysis. The results showed three situations in which the λ genes had the same transcriptional orientation: first, the Igλ locus was flanked downstream by *MRPL40* and *HIRA* and upstream by *GNZA* and *RTDR1*, as in *Homo sapiens* and *X*. *tropicalis*; second, the opposite situation existed, with the Igλ locus flanked downstream by *GNZA* and *RTDR1* and upstream by *MRPL40* and *HIRA*, as in *Gallus gallus*, which can occur via intrachromosomal gene conversion; and third, the Igλ locus was only flanked upstream by *MRPL40* and *HIRA*, as in *Mus musculus* and *Anolis carolinensis*. In the third situation, *GNZA* and *RTDR1* were identified on chromosome 10, which does not contain *IGL* in *Mus musculus*, and in *Anolis carolinensis*, the chromosomal position of *GNAZ* was identified in contig (NW_003341094.1). However, no *IGL* gene was found in this contig, and the *RTDR1* gene was not identified in *Anolis carolinensis*. In *Mus musculus*, the chromosome was recombined, leading to *GNZA* and *RTDR1* being separated from the Igλ locus and located on another chromosome, whereas in *Anolis carolinensis*, the position of *GNAZ* could not be confirmed because of limited genomic data. The Igλ locus of the *Alligator sinensis* was also flanked upstream by *GNZA* and *RTDR1* (Fig 7), whereas *MRPL40* and *HIRA* were located in another *Alligator sinensis* genomic contig (NW_005841997.1), which could not be identified as an *IGL* gene. We cannot confirm that the two contigs of *Alligator sinensis* assembled together due to the preliminary nature of the genome assembly. The results suggested that the position of the Igλ locus on the chromosome in the *Alligator sinensis* was syntenic to that in *Homo sapiens* and *X*. *tropicalis*. In the other species, the flanking genes of the Igλ locus have changed in different ways, including possible intrachromosomal gene conversion (*e*.*g*., *Gallus gallus*), chromosome recombination (*e*.*g*., *Mus musculus*), and others that are not confirmed because of limited genomic data *(e*.*g*., *Anolis carolinensis*). All taxa studied showed the same flanking genes on one side or both sides of the Igλ locus. These data provide convincing evidence that the identified genes originated from the same ancestral gene as the λ gene in tetrapods and originated from the same ancestral gene as the type III light chain gene in *X*. *tropicalis*. The position of the Igλ locus on chromosome in *X*. *tropicalis* may be the oldest form in tetrapods.

**Fig 8:**
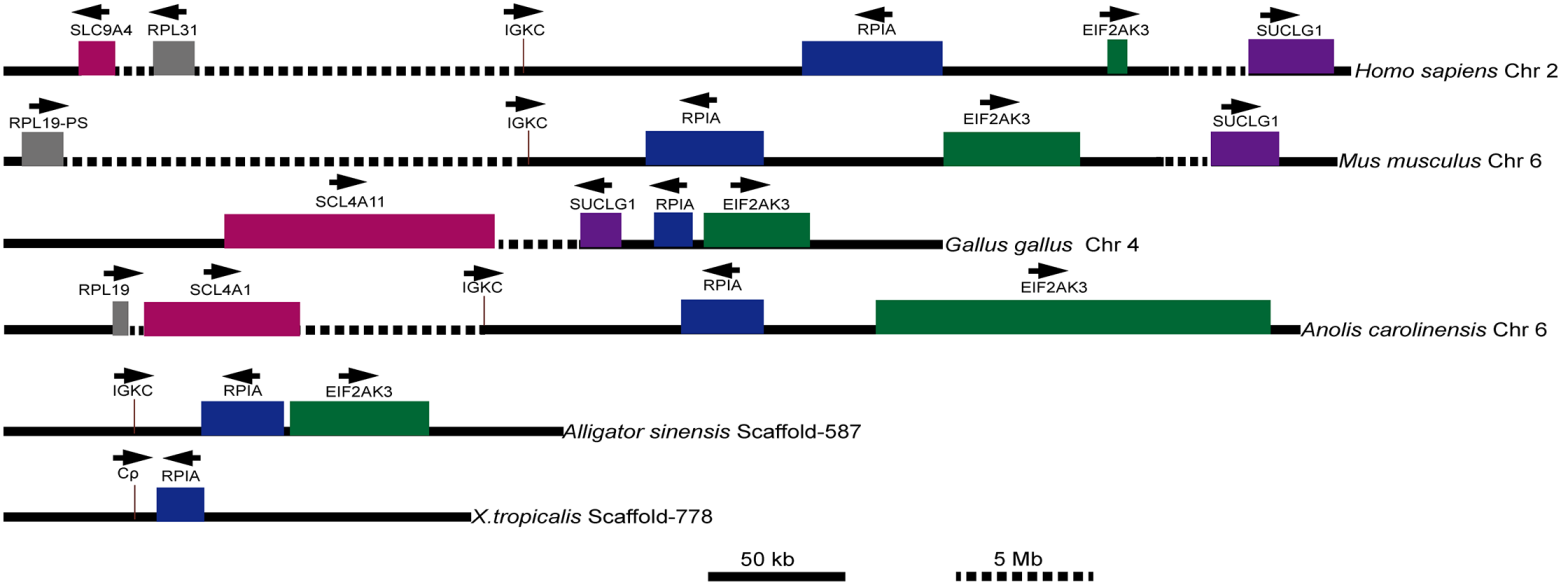
Chromosomal locations of the κ genes in different species and ρ genes in *X*. *tropicalis*. Arrows indicate the transcriptional orientation of the genes. Chr: chromosome; IGκC: immunoglobulin κ chain constant region gene; RPIA: ribose-5-phosphate isomerase A; EIF2AK3: eukaryotic translation initiation factor 2-α kinase 3; SUCLG1: succinate-CoA ligase, GDP-forming, α subunit; SLC4A11: solute carrier family 4, sodium borate transporter, member 11; SLC9A4; solute carrier family 9, sodium borate transporter, member 4; RPL31: ribosomal protein L31; RPL19-PS: ribosomal protein L19, pseudogene 8; RPL19: ribosomal protein L19. SLC4A1: solute carrier family 4, sodium borate transporter, member 1. The figure was modified from Ref. [31].

Similarly, to determine the identified κ genes in the *Alligator sinensis* belonging to the κ lineage, we performed a syntenic analysis of the κ genes using the data available for tetrapods, including *Homo sapiens*, *Mus musculus*, *Gallus gallus*, *Anolis carolinensis* and *X*. *tropicalis*. We used the available long genomic contig (NW_005843366.1) containing the Igκ locus of the *Alligator sinensis* to compare with the chromosomal location relative to the flanking genes of the κ gene in other species. The Igκ loci in all analyzed species, except the *Gallus gallus*, were flanked on the 5’ side by *RPIA* (ribose-5-phosphate isomerase A) and *EIF2AK3* (eukaryotic translation initiation factor 2-α kinase 3) encoding genes (Fig 8), revealing that the Igκ locus of the *Alligator sinensis* was syntenic to the *Homo sapiens*, *Mus musculus*, *Anolis carolinensis* and *X*. *tropicalis*. We also searched for relevant genes upstream of the Igκ locus in the analyzed species and found some gene families that were located far from the Igκ locus, including *SCL* (solute carrier family 4, sodium borate transporter) and *RP* (ribosomal protein). In the analyzed species, either one or two of these gene families were located in the same chromosome with the Igκ locus, except for the *Alligator sinensis* and *X*. *tropicalis*, which lack a complete genomic sequence. Similar to the Igλ locus, we found intrachromosomal gene conversion, as in *Homo sapiens*, *Anolis carolinensis* and *Gallus gallus*, and chromosome recombination leading to lost genes, as in *Mus musculus*. The preservation of the precise order of genes near the Igκ locus on the chromosome suggested that the Igκ of the *Homo sapiens*, *Mus musculus*, *Anolis carolinensis* and *Alligator sinensis* and the ρ of *X*. *tropicalis* was passed down from a common ancestor. However, we did not find any light chain gene located together with the *RPIA* and *EIF2AK3*, but *SUCLG1* (succinate-CoA ligase, GDP-forming, α subunit) was located on the 5’ side of *RPIA* and *EIF2AK3* in the *Gallus gallus*. *SUCLG1* was located downstream from the same chromosome and far from *RPIA* and *EIF2AK3* in the *Homo sapiens* (~4.0 Mb) and *Mus musculus* (~2.4 Mb), suggesting intrachromosomal gene conversion, such as Igλ in the *Gallus gallus*. During this process, the *Gallus gallus* Igκ locus was lost. In *Anolis carolinensis*, *SUCLG1* is located on chromosome 5 rather than on chromosome 6, on which the Igκ locus is located. In *X*. *tropicalis*, gene *EIF2AK3* was not identified with confidence. We also could not identify the gene *SUCLG1* in the *Alligator sinensis*.

### IgL loci functionality and V-J junction diversity in *Alligator sinensis*

Using 5’RACE, we cloned and sequenced 402 amplified cDNA fragments from the blood of *Alligator sinensis* which was the same *Alligator sinensis* to construct genomic BAC library, generating 181 clones that exhibited unique V-J junctions. The sequences were somewhat different from the corresponding genome sequence of the EquCab2 assembly. Among these 181 clones, 56 clones contained a C_λ_1, 44 clones contained a C_λ_2, 32 clones contained a C_λ_3, 3 clones contained a C_λ_4, and 37 clones contained another C_λ_ chain that slightly differed from the identified C_λ_4 gene and shared at least 97.3% sequence identity with C_λ_4, suggesting the existence of an allelic variant of C_λ_4. In addition, two new C_λ_ genes were found in clones LV6-51 and LV61, which were distinct and shared at least 92.7% sequence identity with C_λ_1, C_λ_2, C_λ_3, C_λ_4 and C_λ_5. However, in the rest of the C region, clones exhibited chimeras: clone LV2-11 and clone LV6-91 are C_λ_2-C_λ_1 chimeras; clone LV2-8 and clone LV-14 are C_λ_3-C_λ_2 and C_λ_4-C_λ_1 chimeras, respectively; clones LV11, LV2-38 and LV56 are C_λ_3-C_λ_1 chimeras (S7 Appendix). All chimeras most likely indicated PCR artifacts. The results of the usage of C_λ_ genes and the genomic organization of the Igλ chain gene locus suggested the existence of additional C_λ_ genes in the Igλ locus of *Alligator sinensis*. Furthermore, we could not amplify J_λ_6-C_λ_5 in the *Alligator sinensis* due to its low expression level.

As expected, J_λ_1, J_λ_2, J_λ_3 and J_λ_4 were co-expressed with their respective C_λ_ genes in most cases. However, in some cases, J_λ_ segments were not co-expressed with their respective C_λ_ genes, such as one J_λ_2-C_λ_1 in clone LV25, one J_λ_3-C_λ_2 in clone LV109, one J_λ_1-C_λ_3 in clone LV5-51 and one J_λ_4-C_λ_3 in clone LV6-82, which were generated by template jumping during PCR amplification. Furthermore, two additional C_λ_ genes were not found in the genome and were co-expressed with J_λ_1 and J_λ_2 in clones LV6-51 and LV61, respectively. By alignment, the amino acid sequence identities of the two C_λ_ genes were 97.6% and 98.8% with C_λ_1 and C_λ_2, respectively, suggesting that the two C_λ_ genes in clone LV6-51 and LV61 might be two allelic genes with C_λ_1 and C_λ_2 genes. All three clones containing C_λ_4 and the other 37 clones, which contain an allelic variant of C_λ_4, were co-expressed with J_λ_4, indicating the existence of a C_λ_4 allelic gene. Moreover, we analyzed the J_λ_ genes in 7 chimeras of C_λ_ genes; clones LV2-11 (C2+C1) and LV6-91 (C2+C1) included J_λ_2, clone LV14 (C4+C1) included J_λ_4, and clones LV2-8 (C3+C2), LV11 (C3+C1), LV2-38 (C3+C1) and LV56 (C3+C1) contained J_λ_3. All of these products most likely represented PCR artifacts or were generated by template jumping during PCR amplification. We did not find J_λ_5, J_λ_6, J_λ_7 or any other J_λ_ in the unique 181 clones because of their low expression. We did not find any other C_λ_ genes in our study, although an isolated J_λ_7 was located in the present genomic sequence. It is possible that more C_λ_ genes were not found because of the incomplete genomic data for the *Alligator sinensis*.

**Fig 9:**
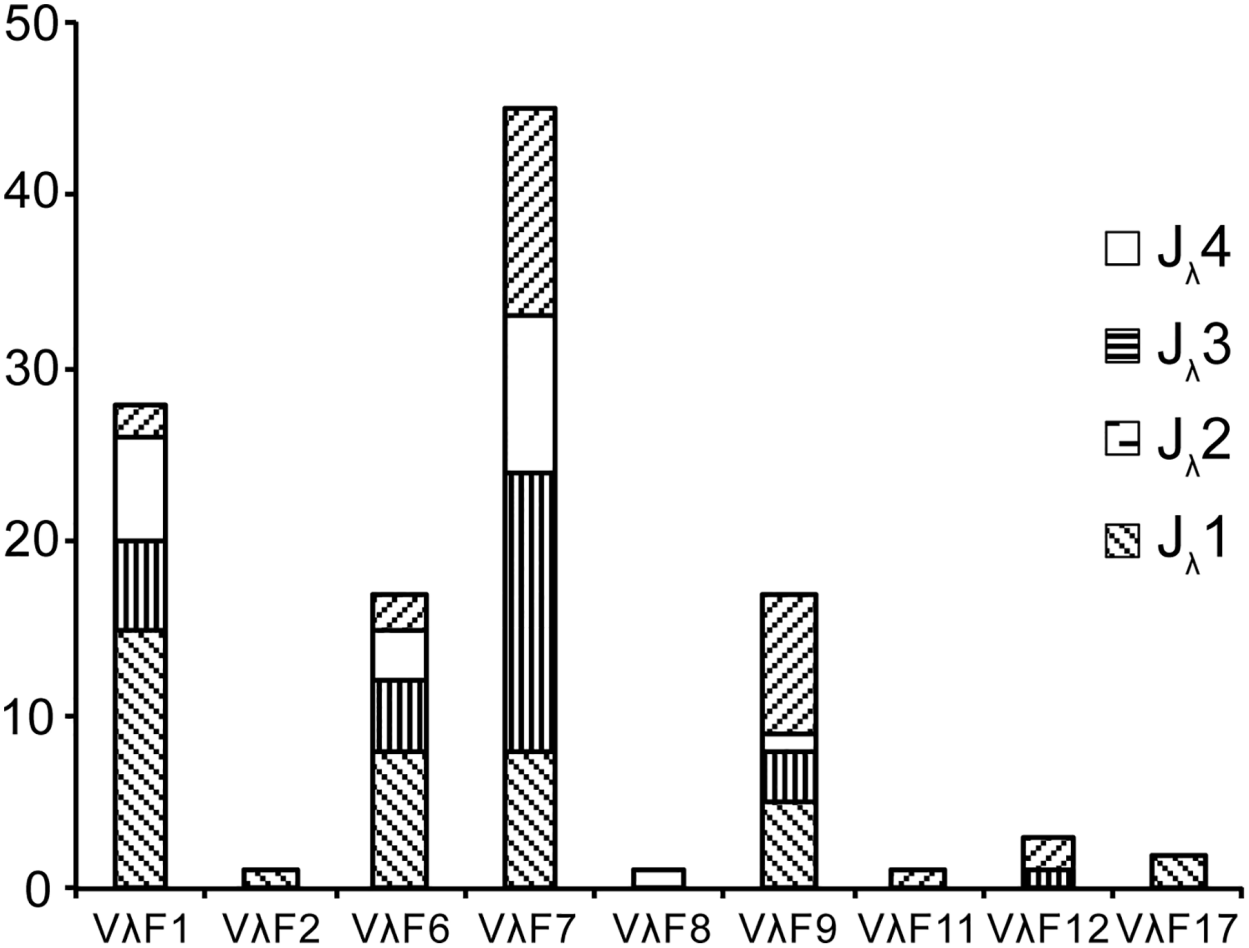
Usage frequency of V_λ_ and J_λ_ genes in the *Alligator sinensis*. The number behind the V_λ_ indicates the number of the family.

Of the 181 cDNA clones described above, 115 had an identifiable V gene, which provided 63 uniquely recombined V-J junctions (S8 Appendix), and were chosen for analysis and revealed a biased usage pattern of V_λ_ (Fig 9). The results showed that V_λ_ segments family 7 was the most frequently used, which accounted for roughly one-third of the expressed V_λ_ repertoire (45/115). Family 1, family 6 and family 9 were more frequently used segments (Fig 9). V_λ_ segments from families 2, 8, 11, 12 and 17 were less frequently used. The V_λ_ segments of other families were not observed in the cDNA clones of the *Alligator sinensis*. In these 63 uniquely recombined V-J junctions, 30% of the clones (35/115) had insertion of N and P nucleotides, generally one to two nucleotides, but there were some exceptions. For example, clone LV6-73 had seven N and P nucleotides in its junction; clone LV5-13 and clone LV5-34 had six and five N and P nucleotides in their junctions, respectively; clones LV6-51 and LV6-8 had four N and P nucleotides in their junctions; and clone LV2-8 had three N and P nucleotides in its junction. On average, the length of the N + P nucleotides in these clones was 0.6 ± 1.2 nucleotides. More than 85% of the clones (98/115) had exonuclease removals at the 3’ end of V_λ_. Compared with V_λ_, fewer nucleotides were removed at the 5’ end of J_λ_ (67/115) by the exonuclease activity (V_λ_ 3.1 ± 2.2 *vs*. J_λ_ 1.6 ± 1.8). The average length of the CDR3 in these λ gene clones was 10.6 ± 0.9 (S8 Appendix). The results above demonstrated the abundant diversity of the V_λ_ genes in the *Alligator sinensis*.

We cloned and sequenced 237 cDNA fragments from the *Alligator sinensis* using 5’ RACE to analyze the use of J_κ_ and V_κ_ segments in the expressed κ chain, among which KV-4 has a stop codon in the leading peptide. After the removal of redundant clones, 124 clones that showed unique V-J junctions were obtained for analysis. All six functional J_κ_ segments were used in these clones: 51 clones contained J_κ_1; 22 clones contained J_κ_2; 18 and 21 clones contained J_κ_3 and J_κ_4, respectively; 10 clones contained J_κ_6; and J_κ_5 was only employed in clone KV-47. In addition, another J_κ_ that was not found in the genome occurred only once in clone KV2-67, suggesting the existence of another J_κ_ in the genome or an allelic variant of J_κ_. The results revealed a preferential J_κ_ segment with J_κ_1 as the first preferential usage. The usage frequencies of J_κ_5 and J_κ_6 were lower, with J_κ_5 being the lowest.

**Fig 10:**
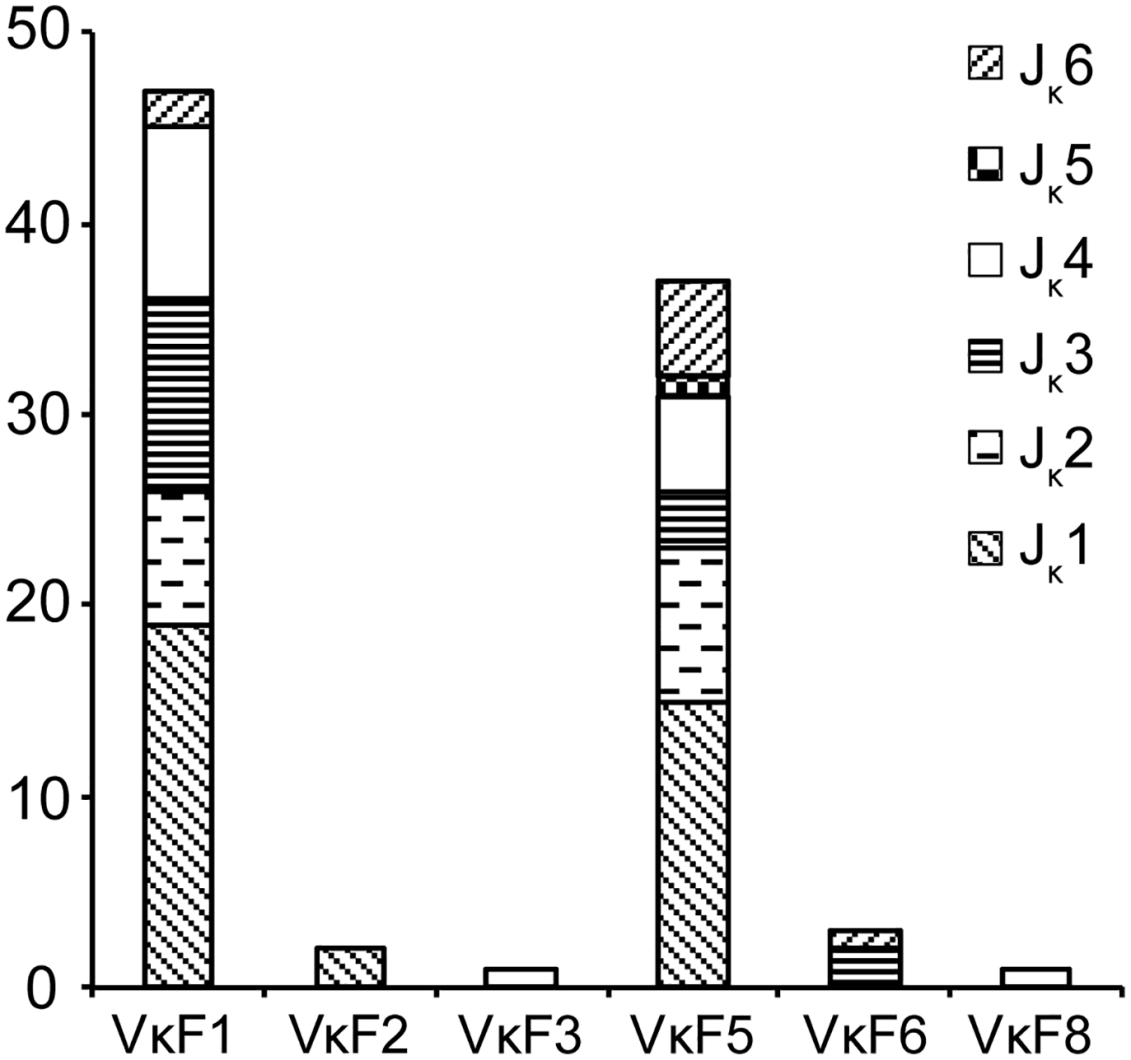
Usage frequency of V_κ_ and J_κ_ genes in the *Alligator sinensis*. The number behind the V_κ_ indicates the number of families.

We chose 91 clones from the above mentioned 124 clones that had identifiable V_κ_ genes for analysis, revealing a preferential V_κ_ usage pattern (Fig 10). The results showed that V_κ_ segments family 1 and family 5 demonstrated obvious advantages, which accounted for 51% and 40% of the expressed V_κ_ repertoire, respectively. V_κ_ segments from families 2, 3, 6 and 8 were less frequently used. The V_κ_ of other families were not observed in the cDNA clones of the *Alligator sinensis* probably because these families contained only one or two members and their expression levels were low. These 91 clones represented 59 uniquely recombined V-J junctions (S9 Appendix). More functional V_κ_ genes that were not found in the genome were expressed in 33 clones, suggesting more V_κ_ genes in the *Alligator sinensis* that have not been identified because of gaps in contigs and incomplete genomic data. The majority of V-J junctions in uniquely recombined κ chain clones lack N and P nucleotide additions. In 59 uniquely rearranged clones, 10 clones show putative N or P nucleotides, and the number of N and P nucleotides is 1 or 2, with an average of 0.16 ± 0.47 bp per clone. The exonuclease removals at the 3’ end of V_κ_ and the 5’ end of J_κ_ were 2.1 ± 1.5 and 1.3 ± 1.7 nucleotides. The average length of the CDR3 was 8.8 ± 0.5 nucleotides, and 89% the expressed κ V-J junctions might be formed by microhomology (S9 Appendix).

## Discussion

Reptilian is comprised of Aves and non-avian reptilia (Crocodylia, Testudines and Squamata) [63, 64]. Immunoglobulin genes have been studied in non-avian reptilia of Testudines species [50, 51] and Squamata species [45, 46, 65–67]. Crocodilians are thought to be the closest relatives of birds, and they are believed to have strong immune systems [52–55]. Recently, the IgH gene of crocodilians was identified [56, 57]. An interesting feature of the crocodilian IgH constant loci is the presence of a number of duplicated genes encoding five Ig classes [57]. In addition, an investigation of the crocodilian α genes suggested that reptiles and birds share a common ancestral organization [56, 57]. To better understand the immune system of crocodilians, to provide a more complete data of crocodilians Igs, and to obtain more information about immunoglobulin evolution in mammals, birds and reptiles, we identified the *Alligator sinensis* IgL gene repertoire based on the genome sequence and *Alligator sinensis* genomic BAC library.

Previous studies suggested that different IgL genes of jawed vertebrates were classified into four isotype groups: λ, κ, σ and σ-cart. To date, all four isotypes are present only in cartilaginous fishes: type I (NS5), type II (NS3), type III (NS5) and σ [13]. Type III is clearly κ, type II is more similar to λ [15, 16], type I is classified as σ-cart [13], and σ is orthologous to the σ isotype in amphibians [13]. Three IgL isotypes exist in amphibians, including λ, κ, and σ [27–31], whereas most other tetrapods, including reptiles, have two IgL isotypes (λ and κ) [5, 7, 32–36, 44]. Birds and snakes have only the λ isotype [39, 42, 45]. The different IgL isotypes are located in different genomic regions. The genomic organizations of these regions are also different [13]. In the κ locus, multiple J_κ_ genes, which are present in different numbers in different species, are present in a cluster and are generally followed by a single C_κ_ [5]. Because the κ isotype is present in cartilaginous and bony fishes, with a clear phylogenetic relationship, and in tetrapods, with the exception of *Gallus gallus*, it is believed to be the oldest and most evolutionarily conserved isotype [13]. Unlike Igκ, the λ gene locus often contains several pairs of J_λ_-C_λ_, which are also present in different numbers in different species, located downstream from the V segments [34]. Previous studies found that multiple J_λ_-C_λ_ were duplicated after speciation [7, 31].

In our recent study, two IgL loci λ and κ were identified in another reptile, the *Alligator sinensis*, using an available genomic database and sequencing of the *Alligator sinensis* genomic BACs, which contain IgL genes. In addition, using the *X*. *tropicalis* C_σ_ as a template [31], we performed a BLAST search against the *Alligator sinensis* whole-genome shotgun sequence assembly. No similar sequence was identified (data not shown). The results are consistent with those for *Anolis carolinensis*, revealing only λ and κ isotypes in reptiles. We sketched the map of the genomic organization of the Igλ and Igκ gene loci of the *Alligator sinensis* (Fig 1; S1 and S5 Figs). As in other species, each C_λ_ gene is preceded by a single J_λ_ gene segment (Fig 1A and S1 Fig), whereas a single C_κ_ gene follows a cluster of J_κ_ gene segments (Fig 1B and S5 Fig). To analyze the structure of the RSS elements flanking the *IGLV* and *IGLJ* genes, the rule of the heptamer-12 bp spacer-nonamer and the nonamer-23 bp spacer-heptamer, which is a universal rule of *IGLV* and *IGLJ* gene in all species, is demonstrated. The results reveal that the genomic organization of Igλ in the *Alligator sinensis* is similar to that in *X*. *tropicalis*, lizards, birds and mammals, whereas Igκ is similar to that in *X*. *tropicalis*, lizards and mammals because the κ gene has been lost in birds. We found six C_λ_ genes and seven J_λ_ genes from the genomic DNA sequence, and the C_λ_5 gene and J_λ_5–7 were not found to be expressed, likely because of their low expression levels. Generally, J_λ_-C_λ_ pairs are located in the genome. In our study, an isolated J_λ_7 was located on the 3’ end of the Igλ locus without following a corresponding C_λ_ gene. This result suggested that more C_λ_ genes might be located in the Igλ locus in the *Alligator sinensis*, which was supported by the Southern blotting results.

Our study also found multiple germline V_λ_ and V_κ_ in the *Alligator sinensis*. A total of 155 V_λ_ and 118 V_κ_ gene segments were identified, which contain 69 V_λ_ pseudogenes and 56 V_κ_ pseudogenes, respectively. All V_λ_ genes are oriented in the same transcriptional orientation as the C_λ_ gene and are upstream of the (J_λ_-C_λ_)_n_ or (J_κ_)_n_. The multiple functional V genes can increase the antibody diversity and enhance the immune response of antigen recognition and binding. The ratio of functional V_λ_ and V_κ_ varies significantly in different species [5, 32–36]. It has been proposed that the number of V gene segments may be connected to the preferential use of light chain isotypes at the protein level [68]. The results of the present study indicated that V_λ_ germline genes are more dominant than V_κ_ (86 functional V_λ_ genes *vs*. 56 functional V_κ_ genes) in the *Alligator sinensis*. It is possible that the λ isotype in *Alligator sinensis* serum antibodies is more abundant than the κ isotype. Additionally, there is a large number of pseudogenes in the V_λ_ and V_κ_ loci. We question whether these pseudogenes are functional as those in birds for use as donors of uniquely combined functional V genes in gene conversion [43]. These pseudogenes were likely involved in generating Ig diversity. The diversification of IgLs in the *Alligator sinensis* is similar to that in most tetrapods but is different from that in the *Gallus gallus*. A total of 142 potentially functional V_λ_ genes (V_λ_ and V_κ_) are classified into 31 families in the *Alligator sinensis*: 19 families in V_λ_ and 12 families in V_κ_ (Figs 3 and 5; S3, S4, S7 and S8 Figs, S3 and S6 Appendixs). For other species, 177 functional V_λ_ genes (V_λ_ and V_κ_) are classified into 23 families in *Mus musculus* (http://www.imgt.org/IMGTrepertoire/), 148 functional V_λ_ genes (V_λ_ and V_κ_) are classified into 23 families in *Homo sapiens* (http://www.imgt.org/IMGTrepertoire/), 51 functional V_L_ genes (V_λ_ and V_κ_) are classified into 11 families in *Anolis carolinensis* [7], and only one V_λ_ gene (or one family) is present in *Gallus gallus* [42]. The diversity of the IgL chain is generated by V-J recombination, somatic hypermutation, and the polymorphism of the V_L_ genes, including the number of V_L_ genes and families (classifying family according to the similarity of sequence). Our results reveal that the *Alligator sinensis* possesses at least 142 functional V_L_ genes (possibly more) and 31 V_L_ gene families, although the number of V_L_ genes in the *Alligator sinensis* is not the most plentiful in the tetrapods. However, the number of V_L_ gene families is the greatest. The phylogenetic analyses show that many V_λ_ gene families in the *Alligator sinensis* are orthologous with other species, but the remaining V_λ_ gene families are characteristic of the *Alligator sinensis*. The *Alligator sinensis* also possesses a large number (68) of *DH* gene segments and multiple μ genes in the IgH locus, suggesting that the *DH* segments may contribute significantly to antibody diversity in crocodilians and that IgM subclasses can be expressed through class-switch recombination in the IgH gene locus [56]. These results reveal the vast diversity of Ig in the *Alligator sinensis*, suggesting that crocodilians have a strong immune system.

We compared IgL chains between two reptiles: the *Alligator sinensis* and *Anolis carolinensis*. We found more abundant V_L_ genes in the *Alligator sinensis* than in *Anolis carolinensis*, including functional V_L_ genes and pseudogenes. The analysis of the expressed V_λ_ and V_κ_ in the *Alligator sinensis* showed that a large number of V genes were employed in both λ and κ, suggesting that somatic V-J recombination can contribute to the *Alligator sinensis* antibody diversity, as in *Anolis carolinensis* [7]. Additionally, the occurrence of N or P nucleotide additions at V-J junctions is increased in the *Alligator sinensis* compared to the paucity of N or P nucleotide additions in the V-J junctions in *Anolis carolinensis*, suggesting that crocodilians have more V-J combinatorial diversity than lizards.

We analyzed the preserved co-localization of genes on the Igλ and Igκ loci in different species. First, we identified a syntenic relationship between two conserved gene clusters the *GNZA* and *RTDR1* cluster and the *MRPL40* and *HIRA* cluster with the Igλ gene on the chromosome in the *Alligator sinensis* and other species, including *Homo sapiens*, *Mus musculus*, *Gallus gallus*, *Anolis carolinensis* and *X*. *tropicalis* (Fig 7). All species retained either one or two gene clusters beside the Igλ locus, although two gene clusters reversed their position in *Gallus gallus* and one gene cluster was lost in *Mus musculus*, suggesting that the location of Igλ locus was conserved in tetrapods, including *crocodilians*. The oldest form was found in *X*. *tropicalis* and *Homo sapiens* and possibly in *Alligator sinensis*. We also found a syntenic relationship of the Igκ gene on the chromosome in different species. The results showed that conserved genes *RPIA* and *EIF2AK3* were flanked on the 3’ side of Igκ in all species, except in *Gallus gallus* (Fig 8). The two gene families, *SCL* and *RPL*, were located far upstream of the Igκ locus. The results suggested that likely intrachromosomal gene conversion occurred in *Gallus gallus* and *Homo sapiens* or *Anolis carolinensis* during speciation, leading to *Gallus gallus* Igλ and Igκ loci changes. The flanking genes of Igλ were reversed and were lost, and the positions of SCL and RPL were reversed in *Homo sapiens* and *Anolis carolinensis*. Either *Homo sapiens* or *Anolis carolinensis* retained the oldest Igκ locus in the genome.

The results of the phylogenetic tree based on the C domain revealed that isotypes were grouped first, and then species were grouped (Fig 5; S9 and S10 Figs). The phylogenetic tree of V genes also showed the same result (Fig 6; S11 and S12 Figs), suggesting that IgL isotypes were individually orthologous. The phylogenetic analyses showed that the σ gene was only present in *cartilaginous* fish, bony fish and amphibians and was absent in reptiles, birds and mammals [13, 24, 31, 39]. The κ gene existed in all vertebrates except birds [13, 39–41]. Therefore, the σ gene was lost in other vertebrates after their divergence from *amphibians* [13, 31], and the κ gene was lost in birds [39–41]. Phylogenetic analysis of the *IGLV* gene, including all 19 V_λ_ families and 12 V_κ_ families in the *Alligator sinensis*, *Alligator sinensis* families V_λ_1-V_λ_8 are related to the *Anolis carolinensis* V_λ_1, V_λ_3, *Gallus gallus* and *Anas platyrhynchos* V_λ_, and *X*. *laevis* type III V4(Fig 6; S11 and S12 Figs), which suggested that during the evolution of the λ locus, there was an ancestral locus shared by birds, reptilia and Salientia [7]. *Alligator sinensis* families V_λ_11 is clustered with *X*. *laevis* type III V6; *Alligator sinensis* families V_κ_11 and V_κ_10 are clustered with *Anolis carolinensis* V_κ_; and *Alligator sinensis* family V_κ_7 is clustered with *X*. *laevis* ρ (Fig 6; S11 and S12 Figs), which indicated that reptilia and amphibians shared some V_λ_ and V_κ_ families and originated from descendants of a common ancestor. Crocodilians possess more V_L_ families than frogs, lizards and mammals, and there is more abundant diversity of the V gene in crocodilians. Taken together, the results strongly suggest that we have identified two IgL loci in *Alligator sinensis* that belong to the κ and λ lineages. We present evidence that the σ was lost in early reptilians, avian and mammalians after their divergence from amphibians [13, 31], and the κ gene was absent in birds after their divergence from reptilians, similar to the δ gene [39–41].

This study investigated the genomic organization of *Alligator sinensis* IgL genes. The organizations and structures of IgL genes are similar to those of other jawed vertebrates. The study of the *Alligator sinensis* λ and κ loci revealed a diverse and complex repertoire of IgL in *crocodilians*; the information provides key insights into the evolution of IgL genes in jawed vertebrates.

## Supporting Information

S1 Appendix Multiple sequence alignment of *Alligator sinensis* V_λ_ genes. (DOCX)

S2 Appendix The *Alligator sinensis* V_λ_ gene DNA segment in contigs. (DOCX)

S3 Appendix The alignment of the deduced amino acid sequence of 86 functional V_λ_ genes in the *Alligator sinensis*. (DOCX)

S4 Appendix Multiple sequence alignment of *Alligator sinensis* V_κ_ genes. (DOCX)

S5 Appendix The *Alligator sinensis* V_κ_ gene DNA segment in contigs. (DOCX)

S6 Appendix The alignment of the deduced amino acid sequence of 62 functional V_κ_ genes in the *Alligator sinensis*. (DOCX)

S7 Appendix Sequence of the C region chimeras in the cDNA clones. (DOCX)

S8 Appendix V-J junctions of the λ chain genes. The letter in the middle indicates N/P nucleotides. The column “N+P” indicates the total nucleotide length of the N and P nucleotides, and the column “CDR3” indicates the codon numbers. The column “Deletions in 3’ end of V_λ_” indicates the number of nucleotides deleted by exonuclease activity at the 3’ end of V_λ_, and the column “Deletions in 5’ end of V_λ_” indicates the number of nucleotides deleted by exonuclease activity at the 5’ end of J_λ_. Germline sequences of each V_λ_ gene segment are shown above the cDNA clones in bold, and the CDR3 is also underlined.
(DOCX)

S9 Appendix V-J junctions of the κ chain genes. The letter in the middle indicates N/P nucleotides. The column “N+P” indicates the total nucleotide length of the N and P nucleotides, and the column “CDR3” indicates the codon numbers. The column “Deletions in 3’ end of V_κ_” indicates the number of nucleotides deleted by exonuclease activity at the 3’ end of V_κ_, and the column “Deletions in 5’ end of J_κ_” indicates the number of nucleotides deleted by exonuclease activity at the 5’ end of J_κ_. Germline sequences of each V_κ_ gene segment are shown above the cDNA clones in bold, and the CDR3 is also underlined.
(DOCX)

S1 Fig The genomic organization of the *Alligator sinensis* immunoglobulin λ gene locus. V: variable gene segments; ΨV: pseudo-variable gene segments; ORF: variable gene segments with open reading frames but with defects in splicing sites, RSS and/or regulatory elements, and/or changing the conserved amino acids, which have been suggested to lead to incorrect folding [69]; J: joining gene segments; C: constant region gene; ΨC: pseudo-constant region gene. Gaps between contigs are indicated by a dotted black line, and the sequences from BAC are indicated by a bold line.
(TIF)

S2 Fig Sequences of the *Alligator sinensis* J_λ_ and C_λ_. (A) Nucleotide and amino acid sequences of the seven *Alligator sinensis* J_λ_ segments. (B) Sequence comparison of the six *Alligator sinensis* C_λ_ genes with their counterparts in the *Homo sapiens*, *Mus musculus*, *Gallus gallus*, *Anas platyrhynchos* and *Anolis carolinensis*. In the alignment, dots indicate identical amino acids and A-G over the lines represent potential IgSF strands. The cysteine (C) and tryptophan (W) residues are shaded.
(TIF)

S3 Fig Phylogenetic trees based on 1000 bootstraps for the *Alligator sinensis* V_λ_ gene segments. The phylogenetic tree was constructed using Phylip3.695 [60] and viewed in TREEVIEW [59].
(TIF)

S4 Fig Phylogenetic analysis of the *Alligator sinensis* V_λ_ gene segments. The tree is made by Neighbor-joining P-distance and pairwise deletions using MEGA6.0.
(TIF)

S5 Fig Genomic organization of the *Alligator sinensis* immunoglobulin κ gene locus. V: variable gene segments; ΨV: pseudo-variable gene segments; ORF: variable gene segments with open reading frames but with defects in splicing sites, RSS and/or regulatory elements, and/or changing the conserved amino acids, which have been suggested to lead to incorrect folding [69]; J: joining gene segments; C: constant region gene. Gaps between contigs are indicated by a dotted black line, and the sequences from BAC are indicated by a bold line.
(TIF)

S6 Fig Sequences of the *Alligator sinensis* J_κ_ and C_κ_. (A) Nucleotide and amino acid sequences of the six *Alligator sinensis* J_κ_ segments. (B) Sequence comparison of the *Alligator sinensis* C_κ_ genes with their counterparts in *Homo sapiens*, *Mus musculus*, *Didelphimorphia*, *Ornithorhynchus*, *X*. *laevis*, *X*. *tropicalis* and *Anolis carolinensis*. In the alignment, dots indicate identical amino acids and A-G over the lines represent the potential IgSF strands. The cysteine (C) and tryptophan (W) residues are shaded.
(TIF)

S7 Fig Phylogenetic trees based on 1000 bootstraps for the *Alligator sinensis* V_κ_ gene segments. The phylogenetic tree was constructed using Phylip3.695 [60] and viewed in TREEVIEW [59].
(TIF)

S8 Fig Phylogenetic analysis of the *Alligator sinensis* V_κ_ gene segments. The tree is made by Neighbor-joining P-distance and pairwise deletions using MEGA6.0.
(TIF)

S9 Fig Phylogenetic trees based on 1000 bootstraps for the IgL chain C genes in jawed vertebrates. The phylogenetic tree was constructed using Phylip3.695 [60] and viewed in TREEVIEW [59].
(TIF)

S10 Fig Phylogenetic analysis of the IgL chain C genes in jawed vertebrates. The phylogenetic tree was constructed using C domains, and by Neighbor-joining P-distance and pairwise deletions using MEGA6.0.
(TIF)

S11 Fig Phylogenetic trees based on 1000 bootstraps for the IgL chain V genes in jawed vertebrates. The phylogenetic tree was constructed using V domains. Each V subgroup is represented with one sequence per species chosen at random among the functional genes. The scale shown as a bar represents the genetic distance (number of nucleotide changes in the given scale). The credibility value for each node is shown. The phylogenetic tree was constructed using Phylip3.695 [60] and viewed in TREEVIEW [59].
(TIF)

S12 Fig Phylogenetic analysis of the IgL chain V genes in jawed vertebrates. The phylogenetic tree was constructed using V domains, and by Neighbor-joining P-distance and pairwise deletions using MEGA6.0.
(TIF)

S1 Table Primers used for screening BACs. (DOCX)

S2 Table Summary of the *Alligator sinensis* germline V_λ_ in contigs. (DOCX)

S3 Table Summary of the *Alligator sinensis* germline V_κ_ in contigs. (DOCX)
