# Supplementary material for: A Comprehensive Analysis of the Phylogeny, Genomic Organization and Expression of Immunoglobulin Light Chain Genes in Alligator sinensis, an Endangered Reptile Species
Source: PLoS One. 2016 Feb 22;11(2):e0147704. doi: 10.1371/journal.pone.0147704 (PMC4762898; doi:10.1371/journal.pone.0147704)
Supplement: S1 Appendix — (DOCX) [file pone.0147704.s001.docx]

**Multiple sequence alignment of *Alligator sinensis* V_λ_ genes**

* 20 * 40 * 60 * 80 * 100 * 120 * 140 * 160

VL1 : GCCAACTCACAGCCTGCGCTCACTCAGCCGCCCGCAGAGTCA---GTGTCCCCAGGAAACACTGTGAAACTCTCCTGCACCATGAGCAGTGGGACCAGCATCAGTGACTATGCTGTGTACTGGTACCAGCAGAAACCTGGGACCCCCACTCGGTACCTGCTG

VL2 : TCCCTTGCCGAATACGTGCTGACACAGCCGCCCGAAGTGTCT---GTGTCTCCAGGACAAACTGCTCAGCTCACGTGCAGGGGAGAAAAGT---------TTGATAAATACTACGTGTACTGGTACCAGCAGAAATCTGGCAGTGTCCCCAAGC---TTGTG

VL3 : GCCAGATCACAGTCTGTGCTCACTCAGCCGCCCACAGAGTCA---GTGTCCTTAGGAAACACTGTGAAACTCTCCTGCACCGTGAGCAGTGGGACCAGCATCAGTGACTACAATGTGTACTGGTACCAGCAGAAACCTGGGACCCCCCCTCGGTACCTGCTG

VL4 : TCCCTTGCTGAGCATGTGCTGGTACAGCCGCCCGCAGTGTCT---GTGTCTCCAGGACAAACTGCTCAGATCACGTGCAGCGGAGAAATGC---------TTACAAAGAACTATGCACAATGGTACCAGCACAAACCTGGCAGCGTCCCCAAGC---TTGTG

VL5 : GCCAGATCACAGTCTGTGCTCACTCAGCCGCCCACAGAGTCA---GTGTCCTCAGGAAACACTGTGAAACTCTCCTGCGCCATGACCAGTGGGACCAGCATCAGTGGCTACAGTGTGTACTGGTACCAGCAGAAACCTGGGACCCCGCCTCGGTACCTGCTG

VL6 : GCCAGATCACAGTCTGTGCTCACTCAGCCGCCCGCAGAGTCA---GTGTCCCCAGGAAACACTGTGAAACTCTCCTGCGCCATGAGCAGTGGGAACAGCATCAGTGTCTACTATGTGAATTGGTACCAGCAGAAACCTGGGACCCCCCCTCGGCAGCTGCTG

VL7 : TCCCTTGCCGAGTACGTGCTAACGCAGCCGCCCGCAGTGTCT---GTGTCTCCAGGACAAACTGCTCACATCACGTGCAGTGGAGAGAAGC---------TTAACAAGAAGTATGCACAATGGTACCAGCAGAAACCTGGCAGCGTCCCCCGAC---TTCTT

VL8 : TCCCTTGCTGAGCACGTGCTGGTACAGCCGCCCGCAGTGTCT---GTGTCTCCAGGACAAACTGCTCAGATCACGTGCAGCGGAGAAATGC---------TTACAAAGAGGTATGCACAATGGTACCAGCACAAACCTGGCAGCGTCCCCAAGC---TTCTG

VL9 : GCCAGATCACAGTCTCTGCTCACTCAGCCGCCCGCAGAGTCA---GTGTCCCCAGGAAACACTGTGAAACTCTCCTGCGCCGTGACCAGTGGGACCAGCATCAGTGGCTACAGTGTGCTCTGGTACCAGCAGAAACCTGGGACCACCCCTCGGTACCTGCTG

VL10: GCCAGATCACAGTCTCTGCTCACGCAGCCTCCTTCAGAGTCA---GTGTCCCCAGGAAACACGGTGAAACTTTCCTACACCATGAGCAGTGAGGACAGCATCAGTGGCTCTTTTACACACTGGTACCAGCAGAAACCTGGGACCCCTCCTCGATACCTGCTG

VL11: TCCCTTGCCCAGTACGTGGTGACGCAGCCGCCTTCAGTGTCT---GTGTCTCCTGGAGAAACCACTCGGCTCACCTGCTCCGGGAACAGCA---------TTAGTGGCAAGTATGTGCAGTGGTACCAGCAGAAACCTGGCACTGCCCCTCTAC---TCATT

VL12: TCCCTTGCCCAGTACGTGGTGACGCAGCCGCCTTCAGTGTCT---GTGTCTCCAGGACAGACTGCTCGGCTCACCTGCTCCGGGAACAACA---------TTGGTAGGAAGAGTGTGCACTGGTACCAGCAGAAACCCAGCAGTGCCCCTCTAC---TCATT

VL13: TCCAGCTCCCAGTCTGTGGTGACGCAGCCGCCCGCAGAGTCA---GTGTCCCCGGGAAACACTGTGAAACTCTCCTGTGCCATGAGCAGTGGGACCAGCATCAGTGGCTACAATGTGTACTGGTTTCAGCAGAAACCTGGGACCCCCCCTCGGTACCTGCTG

VL14: TCCCTTGCCCAGTATGTGGTGACACAGCCGCCTTCAGTGTCT---GTGTCTCCAGGACAAACTGCTCGGCTCACCTGCTCCGGGAACAACA---------TTGGTAGCAAGAGTGTGCACTGGTACCAGCAGAAACCTGGCACTGCCCCTCTAC---TCATT

VL15: TCCAGCTCCCAGTCTGTGGTGACGCAGCCGCCCGCAGAGTCA---GTGTCCCCGGGAAACACTGTGAAACTCTCCTGCACCATGAGCAGTGGGACCAGCATCAGTGGCTACTATGTGAACTGGTTTCAGCAGAAACCTGGGACCCCCCCTCGGTACCTGCTG

VL16: TCCCTTGCCCAGTATGTGGTGACACAGCCGCCTTCAGTGTCT---GTGTCTCCAGGACAGACTGCTCGGCTCACCTGCTCCGGGAACAACA---------TTGGTAGCAAGAGTGTGCACTGGTACCAGCAGAAACCCGGCAGTGCCCCTCTAC---TCATT

VL17: TCCCTTGCCCAGTACGTGATGACACAGCCGCCTTCAGTGTCT---GTGTCTCCAGGACAAACTGCTCGGCTCACCTGCTCCGGGAACAACA---------TTGGTAGCAAGAGTGTGCACTGGTACCAGCAGAAACCTGGCAGTGCCCCTCTAC---TCATT

VL18: TTGTTTGCCCAGAACGTGGTAACGCAGCCACCCGCAGTGTCT---GTGTCTCCAGGACAAACTGCTCGGCTCACCTGCTCTGGGACT------------------GGCAGCTATGTCCACTGGTACCAACAGAAACCTGGCAGTGCCCCCCTAC---GCATT

VL19: TCCAGCTCCCAGTCTGTGGTGACGCAGCCGCCCGCAGAGTCA---GTGTCCCCGGGAAACACTGTGAAACTCTCCTGCACCATGAGCAGTGGGACCAGCATCAGTGGCTACAGAGTGTACTGGTTTCAGCAGAAACCTGGGACCCCCCCTCGGCAACTGCTG

VL20: TTGTTTGCCCAGAACGTGGTGACGCAGCCACCCGCAGTGTCT---GTGTCTCCAGGACAAACTGCTCGGCTCACCTGCTCTGGGCCT------------------GGCAGCTACATGCACTGGTACCAGCAGAAACCCGGCAGTGCCCCCCTAC---TCATT

VL21: TCCCTTGCCCAGTACGTGCTAACTCAACCGCCTTCAGTGTCT---GTGTCTCCAGGACAAACCGCTCCGCTCACCTGCTCCGGGACTAACA---------TTGGTAGCTACAATGTGCAATGGTACCAGCAGAAACCTGGCAGTGCCCCTCGAC---TCATT

VL22: TTGTTTGCCCAGAACGTGGTAACGCAGCCACCCGCAGTGTCT---GTGTCTCCAGGACAAACTGCTCGGCTCACCTGCTCTGGGACT------------------GGCAGCTATGTCCACTGGTACCAACAGAAACCTGGCAGTGCCCCCCTAC---TCATT

VL23: TCCAGCTCCCAGTCTGTGGTGACACAGCCGCCCGCAGAGTCA---GTGTCTTCGGGAAACACTGTGAAACTCTCCTGCGCCATGAGCAGTGGGACCAGCATCAGTGGCTACTATGTGTACTGGTTTCAGCAGAAACCTGGGACCCCCCCTCGGCACCTGCTG

VL24: TCCCTTGCCCAGTATGTGGTGACGCAGCCGCCTTCAGTGTCT---GTGTCTCCTGGAGAAACCACTCGGCTGACTTGCTCTGGGAACAACA---------TTGGTAGCAAGAGTGTGCACTGGTACCAGCAGAAACCTGGCACTGCCCCTCTAC---TCATT

VL25: TTGTTTGCCCAGAACGTGGTAACGCAGCCACCCGCAGTGTCT---GTGTCTCCAGGACAAACTGCTCGGCTCACCTGCTCTGGGCCT------------------GGCAGCTATGTCTACTGGTACCAACAGAAACCTGGCAGTGCCCCCCTAC---TCATT

VL26: TCTCTTGCCCAGTACGTGCTGACTCAGCCGCCTGCAGCGTCC---GTCTCCCCGGGACAGACTGCCCGGCTCACCTGCACAGGGACCAGTT---------TTAGGAGCTACTATGTACAGTGGTTCCAGCAGAAACCCGGCAGTGCCCCCCTGC---AGCTG

VL27: TCTCTTGCCCAGTATGTGCTGACTCAGCCGCCCGCAGCATCT---GTCTCCCCAGGACAGACTGCCCGGCTCACCTGCACAGGGACCAGTC---------TTGGGACCTACCATGTACAGTGGTTCCAGCAGAAACCCGGCAGTGCCCCCCTGC---AGCTG

VL28: TCTCTTGCCCAGGATGTGCTGAGACAACCGCCTTCAGTGTCT---GTGTCTCCAGGACAAACTGCTAAACTCACCTGCACAGGAGAGGACT---------TTGATAGCAATGAAGTGAGCTGGTACCAACAGAAACCTGGCAGTGCCCCTCTGC---TCCTC

VL29: TCTCTTGCCCAGGATGTGCTGAGACAACCGCCTTCAGTGTCT---GTGTCTCCAGGACAAACTGCTAAACTCACCTGCACAGGAGAGGACT---------TTGATAGCAATGCAGTGAGCTGGTACCAACAGAAACCTGGCAGTGCCCCTCTGC---TCCTC

VL30: TCTCTTGCCCAGTATGTGATGACTCAGCTGCCTTCAGTGTCT---GTCTCTCCAGGACAGACTGCCATGATCACCTGCATGGGAAGAAATG---------TGGATAGCAACAGCATGCAGTGGTTCCAACAGAAACCTGGGAGTGCCAATCTGC---TGCTT

VL31: TCTCTTGCCCAGGATGTGCTGAGACAACCGCCTTCAGTGTCT---GTGTCTCCAGGACAAACTGCTAAACTCACCTGCACAGGAGAGGACT---------TTGATAGCAAGATAATGAGCTGGTACCAACAGAAACCTGGCAGTGCCCCTCTGC---TCCTC

VL32: TATCTTGCCCAGTACATGCCAACTCAGCCACCCATAGTATCT---GTGTCTCCAGGACAAACTGCTCAGATCACCTGCACAGGGAATAATG---------TTGGTGGCCACAATGTGCACTGGTACCAACAGAAGCCTGGCAGTGTCCCCCTGC---TGATC

VL33: GCTGGTGCCCAGTTCGTGCTGACTCAGCCGCCCTCGGTGTCG---GCGTCTCCCGGAGGAACAGTTACCATCACCTGTGCCAGGAGCAGCGGCA---GCATCAGTGACAGCTGGAACTCCTGGTACCAGCAGAAGCCTGGCAGGGCTCCGGTGA---TGGTT

VL34: TGTCTTGCTCAGACCGTGCTGACTCAGCCACCTTTGGTGTCC---ATATTTCCTGGACAAACTGCTACGCTCACCTGCACAGGAAATAGCA---------TCAGTAGCCATGGACCAGATTGGTACCAACAGAAACCTGGGAGTGTCCCCTTGC---TCGTT

VL35: TCTCTTGCTGAGAGCATGACAACTCAGCCGCCTTCGGTGTCC---GTCTCTCCCGGACAAACTGCTACACTCACCTGCACAGCAAATACCA---------TTAGTGGTTTCGGAGCAAGTTGGTACCAACAGAAACCTGGGAGTGCCCCCCTGA---TCATT

VL36: TCCCTGGCCCAGTACGTGATGACACAGCCACCTTCAGTGTCC---ATCTCACCAGGACAAACTGCTAAACTGACCTGCACAGGGAGAAATG---------TTGGTAACTA---TGTGAGTTGGCACCAACAGCAACCTGGCAGTGCCCCTGTGC---TGTTT

VL37: TCCCTGGCCCAGTACGTGATGACACAGCCACCTTCAGTGTCC---ATCTCACCAGGACAAACTGCTAAACTGACCTGCACAGGGAGAAATG---------TTGGTAACTA---TGTGAGTTGGCACCAACAGCAACCTGGCAGTGCCCCTGTGC---TGTTT

VL38: TGTCTTGCTCAGACCGCGCCGACTCAGCTGCCTTCGGTGTCC---GTCTCTCCCGGACAAACTGCTACGCTCACCTGCACGGGCAGTAGCA---------TTGGTAGCTACGGAGCGGGTTGGTACCAACAGAAACCTGGGAGTGCCCCCCTGC---CCATT

VL39: TGTCTTGCTCAGACCGCGCCGACTCAGCCGCCTTCGGTGTCC---GTCTCTCCCGGACAAACTGCTACGCTCACCTGCACGGGCAGTAGCA---------TTGGTAGCTACGGAGCGACTTGGTACCAACAGAAACCTGGGAGTGCCCCCCTGA---CCGTT

VL40: TGTCTTGCTCAGACCGCGCCGACTCAGCCGCCTTCGGTGTCC---GTCTCTCCCGGACAAACTGCTACGCTCACCTGCACGGGGAGTAGCA---------TTGGTATCGTTGGAGCGAGTTGGTACCAACAGAAACCTGGGAGTGCCCCCCTGC---TCGTT

VL41: TCTCTTGCCCAGTACGTGATGACTCAGCCACCTTCAGTGTCC---GTCTCTACAGCGGAAACTGCTAAAATTACCTGCACAGGAAGAAACG---------TTGGTAGCAAGCATGTACACTGGTTTCAACATAAACCTGGCAGTGCCCCTGTGC---GGATC

VL42: TGTCTTGCCCAGTATGTGATGACTCAGCCACCTTCAGTGTCC---ATCCCTTCAGGGGAAACTGCCAAACTCACCTGCACAGGAAGAAATG---------TTGCTAGCCATGGTGTGCAGTGGTTTCAACAGAAGCCTGGCAGTGCCCCTGTCC---AGATT

VL43: TCACTTGCCCAGTACGTGATGACTCAGTCACCCTCATTGTCC---GTCTCTCCAGGCGAAACTGCTAAACTCACCTGCACGGGAAGAAATA---------TTGGCAGCAACTATGTGCAGTGGTATCAACAGAAATCTGGCAGTGCCCCTGTGC---TGATT

VL44: TCCTTTGCCCAATATGTGATGACTCAGCCACCTTCAGTGTCC---GTCTCTCCAGGGGAAACTGCTAAACTCACCTGCACAGGAAGAAACG---------TTGGTCGCTACAGTCTGCAGTGGTTTCAACAAAAACCTGGCAGTGCCCCCCTGC---AAATT

VL45: GCCAGCTCACAGCCTGCACTGACTCAGCCACCCTCAGAGTCA---GTGTCCCCAGGAAACACTGTCAAACTCTCCTGCACCATGAGCAGTGGGACCAGCATCAGTGATTATGTTATAAGTTGGTACCAGCACAAAGCTGGGAGTGGCCCTCGATACCTGCTG

VL46: TCCCTTGCCCAGTACGTGCTGACTCAGCCGCCTTCAGTGTCT---GTGTCTCCAGGACAAACCGCTCAGCTCACCTGCTCCGGGGACAATG---------TTGCTAGCAAGAATGTGTATTGGTATCAGCATAAAGTCAGCAGCTCCCCTGTAC---TTGTG

VL47: TCCCTCTCCCAGTACACGCTGACTCAGCCTTCCTCAGAGTCT---GTGTCACTAAGAAACACGGTGAAGCTCTCCTGCACCATCAGCAGTGGATACAC------ACATGGAGCTGTCTACTGGTTGCAGCAGAAAGGCGGGAACAGCCCAAGATACCTTCTG

VL48: GTCTGGTTGCAGCCTGTGGTGACCCAGGAGCCCTTGGCATCATCAGTGTCCCCAGGGGAGACTGTCACTCTGTCCTGCAGCCTGAGCACCGGAGCCATCACCAGCAGCAACCATCCTTCCTGGTACCAGCAGAAACCTGGCTCTCCCCCTCGGT---TGCTT

VL49: TCCCTTGCCCAGTACGTGCTGACTCAGCCGCCTTCAGTGTCT---GTGTCTCCAGGACAAACCGCTCAGCTCACCTGCTCCGGGGACAATG---------TTGCTAGCAAGAATGTGTATTGGTATCAGCATAAAGTCAGCAGCTCCCCTCTAC---TTGTG

VL50: TCACTCTCCCAGTTCACCCTGACTCAGCCACCCTCAGAGTCC---GTGTCCCTGGGAAACACAGTTAAACTCTCCTGCACCATCAGCAGTGGAAAAAC------ATTGGGAGGTGTTGCCTGGTACCAGCAAAAAAATGGGAACAGCCCAAGATACTTGTTG

VL51: TGTCTTGCTCAGACCGCGCCGACTCAGCCGCCTTCGGTGTCC---GTCTCTCCCGGACAAACTGCTACGCTCACCTGCACGGGCAGTAGCA---------TTGGTAGCTATGGAGCAGCTTGGTACCAACAGAAACCTGGGAGTGCCCCCCTGA---CCGTT

VL52: TCCCTGCCCCAGTACGTGATGACGCAGTCACCTTCGGTGTCC---ATCTCTCCGGGACAAACTGCTAAACTGACCTGCACAGGAAGAAATG---------TGGGTAGCTACAGTGTGAGTTGGGACCAACAGAAACCTGGCAGCGTTCCCCTGC---TGCTT

VL53: TCACTTGCCCAGTACGTGATGACACAGTCGCCCTCAGTGTCT---GTCTCTCCAGGGGAAACTGCTAAACTCATCTGCACGGGAAGAAATA---------TTGACAGCAACTATGTGCAGTGGTATCAACAGAAATCTGGCAGTGCCCCTGTGC---TGATT

VL54: TGTCTTGCTCAGACCGCGCCGACTCAGCCGCCTTCGGTGTCC---GTCTCTCCCGGACAAACTGCTACGCTCACCTGCACGGGGAGTAGCA---------TTGGTAGCTACGGAGCGAGTTGGTACCAACAGAAACCTGGGAGTGCCCCCCTGA---CCGTT

VL55: TCCCTGGCCCAGTACGTGATGACGCAGTCACCTTTGGTGTCC---GTCTCTCCAGGACAAACTGCTAAACTGACCTGCACAGGAAGAAATG---------TGGGTAGCTACAGTGTGAGTTGGGATCAACAGGAACCTGGCAGCGTTCCCCTGC---TGCTT

VL56: GTCTGGTCCCAGCCTGTGTTGACCCAGGAGCCCTCGGCATCTTCAGTGTCCCCAGGAGGCACTGTCACTCTGTCCTGCAGCCTGAGCACCGGAGCCATCACCACCAGCAACTATCCTGCCTGGTTCAGGCAGAAACCTGGCTCTGCCCCTCAGC---AGCTT

VL57: TCCCACTCCCAGCCGACGCTCACCCAGGCGCCCTCAGAGTCT---GTGTCCCTGGGAAACACCATTAAACTCTCCTGCACGCTGAGCAGCCAGCACAG------TAACTATGTTATCTCCTGGTACCAACAACAACCAGGCCAAACCCTACGAT---TTCTA

VL58: TCCCTCTCACAGCCCACACTGACTCAACAGCCCTCAAAGTCC---GTGACCCTTGGCAACACTGTCAGTCTCTCTTGTACACTGAGCAGTGAGCACAG------TAACTACTATGTCCACTGGTACCAACAAAAACAGGGCCAGGCCCCACAAT---TTCTC

VL59: TCATTTTCTCAAATTACACTGACTCAGCCATCCTCGGAGTCT---GTGTCCCTGGGAAACACCATTAAACTCTCCTGCACACTCAGCAGCGGAGGTAC------ATTTGGAGCTGTCTACTGGTTGCAGCAGAAAGGTGGGAACAGCCCAAGATACCTTCTG

VL60: TCCCTGTCGCAGCCTACGCTGACCCAGCCGCCCTCTGAGTCT---GTGTCCCTGGGAAACACTGTCAGTCTGTCCTGCACGCTGAGCAGTCAGTACAG------TAACTACTATGTTGCTTGGTACCAACAAAGATCGAGCCAGGCCCCACGAT---TTCTC

VL61: GTCTGGTCACAGCCTGTGGTGACTCAGGAGCCTGCGATGTCC---GTGTCCCCAGGAGGGACTGTCACTCTGTCCTGCAGCCTGAGCACCGGAGCCATCACCTCCAGCAACTATCCTGGCTGGCTCCAGCAGAAACCTGGCTCTCCCCCTCGGT---TGCTT

VL62: GTCTGGTCACAGCCTGTGGTGACTCAGGAGCCTGCGGTGTC---GGTGTCCCCAGGAGGGACTGTCACTGTGTCCTGCAGCCTGAGCACTGGAGCCATGACCAGCAATAACTATCGTGGGTGGTACCAGAAGAAACCTGGCTCTGCCCCTCAGT---TGTTA

VL63: GTCAGGTCTCAGTCTGTGGTGACTCAGGAGCCATCCCTGTCA---GTGTCCCCAGGAGGGACTGTCACTCTGTCCTGCAGCCTGAGCACCGGAGCCATCACCACCAGCAACTATCCTGGCTGGTACCAGCAGAAACCTGGCTCTCCCCCTCGGC---AGCTT

VL64: GCTGGTGCCCAGTTCGTGCTGACTCAGCCGCCCTCGGTGTCG---GCATCTCCCGGAGGAACAGTTACCATCACCTGTGCCAGGAGCAGCGGCA---GCTTCAGTGACAGCTGGAACTCCTGGTACCAGCAGAAGCCTGGCAGGGCTCCGGTGA---TGGTT

VL65: GTCTGGTCACAGCCTGTGGTGACTCAGGAGCCTGCGGTGTCC---GTGTCCCCAGAAGGGACTGTCACTCTGTCCTGCAGCCTGAGCACCGGAGCCATCACCTCCAGCAACTACCCTGGCTGGCTCCAGCAGAAACCTGGCTCTCCCCCTCGGC---AGCTT

VL66: GTCTGGTCACAGCCTGTGGTGACTCAGGAGCCTGCGGTGTCCTCAGTGTCCCCAGGAGGGACTGTCACTCTGTCCTGCAGCCTGAGCCCTGGAGCCATCAGCACTGGTTACTACCCTGCCTGGTACCAGCAGAAACCTGGCTCTCCCCCTCAGA---TGATT

VL67: GCTGGTGCCCAGTTCGTGCTGACTCAGCCGCCCTCGGTGTCG---GCATCTCCCGGAGGAACAGTTACCATCACCTGTGCCAGGAGCAGCGGCA---GCATCAGTGACAGCTGGAACTCCTGGTACCAGCAGAAGCCTGGCAGGGCTCCGGTGA---TGGTT

VL68: GTCTGGTCACAGCCTGTGGTGACTCAGGACCCTGTGATGTCC---GTGTCCCCAGAAGAGACTGTCACTCTGTCCTGCAGCCTGAGCACCGGAGCCATCACCTCCAGCAACTACCCTGGCTGGCTCCAGCAGAAACCTGGCTCTCCCCCTCGGT---TGCTT

VL69: GTCTGGTCACAGCCTGTGGTGACTCAGGAGCCTGCGCTGTCG---GTGGCCCCAGGAGGGACTGTCACTCTGTCCTGCAGCCTGAGCACCGGAGCCATCACCACCAACAACTACCCTGGCTGGTACCAGAAGAAACCTGGCTCTCCCCCTCGGC---AGCTT

VL70: TCCAGTTCCCAGCAGCTGCAGCCCCTGACGCCGTCAGAGCAG---GTGTCTCCCGGAGGGACTGTGACCATCGCGTGCAGCCTGAGCAGTGGAGCAGTTGCTGATAACAGCTACATACACTGGCTGCAACAGAAACCGGGCCAGGCTCCTCGGC---TGCTG

VL71: TCCAGTTCCCAGCAGCTGCAGTCGCTGAAGCCGTCAGAGCAG---GTGTCTCCCGGAGGGACTGTGACCATCACGTGCAGCCTGAGCAGTGGAGCAGTTGGTGATGGCAACTACCCACAGTGGGTGCAACAGAAACCGGGCCAGGCTCCTCAGC---TGCTG

VL72: GTCTGGTCGCAGCCTGTGGTGACTCAGGAGCCTGCGGTGTCC---GTGTCCCCAGGAGGGACTGTCACTCTGTCCTGCAGCCTGAGCACCGGAGCCATCACCTCCAGCAACTATCCTGGTTGGTACCAGCAGAAACCTGGCTCTCCCCCTCGGT---TGCTT

VL73: GTCTGGTCACAGCCTGTGGTGACTCAGGAGCCTGCGCTGTCC---GTGTCCCCAGGAGGGACTGTCACTCTGTCCTGCAGCCTGAGCACCGGAGCCATCACCAGCAGGAACTATCCTGCCTGGTTCCAGCAGAAACCTGGCTCTCCCCCTCGGC---AGCTT

VL74: GTCCGGTCGCAGCCTGTGGTGACTCAGGAGCCTTCAGTGTCTTCGGTGTCCCCAGGAGGGACTGTCACTCTGTCCTGCAGCCTGAGCACCGGAGCCATCACCACTGGCAACTATCCCTCCTGGTACCAGCAGAAACCTGGCTCTGCCCCTCAGC---TGCTT

VL75: GTCTGGTCACAGCCTGTGGTGACTCAGGAGCCTGTGATGTCC---GTGTCCCCAGGAGGGACTGTCACTCTGTCCTGCAGCCTGAGCACCGGAGCCATCACCACCAGCAACTATCCTGCCTGGCTCCAGCAGAAACCTGGCTCTCCCCCTCGGC---AGCTT

VL76: GTCTGGTCGCAGCCTGTGGTGACTCAGGAGCCTTCAGTGTCTTCGGTGTCCCCAGGAGGGACTGTCACTCTGTCCTGCAGCCTGAGCACCGGAGCCATCACCACTGGCAACTATCCCTCCTGGTACCAGCAGAAACCTGGCTCTGCCCCTCAGC---TGCTT

VL77: GTCTGGCCCCGTCCTGTGGTGACTCAGGAGCCTGCGGTGTCCTCAGTGTCCCCAGGAGGGACTGTCACTCTGTCCTGCAGCCTGAGCACCGGAGCCATCACCAGCAGCAACTACCCTGGCTGGCTCCAGCAGAAACCTGGCTCTGCCCCTCGGC---AGCTT

VL78: TCCAGTTCCCAGCAGCTGCAGTCGCTGAAGCCGTCAGAGCAG---GTGTCTCCAGGAGGGACTGTGACCATCGCGTGCAGCCTGAGCAGTGGAGCAGTTGGTGATGGCAACTACCCACAGTGGGTGCAACAGAAACCGGGCCATGTTCCTCGGC---TGCTG

VL79: GTCTGGTCACAGCCTGTGGTGACTCAGGAGCCTGCGGTGTCC---GTGTCCCTAAGAGAGATTGTCACTCTGTCCTGTAGCCTGAGCACCGGAGCCATCACCAGCACCAACTATCCTCACTGGTTCCAGCAGAAACCTGGCTCTGCCCCTCGAC---AGCTT

VL80: GTCTGGTCACAGCCTGTGGTGACTCAGGAGCCTGCGGTGTCC---GTGTCCCCAGGAGGGACTGTCACTCTGTCCTGCAGCCTGAGCACCGGAGCCATCACCTCCAGCAACTACCCTGGCTGGCTCCAGCAGAAACCTGGCTCTGCCCCTCGGC---TGCTT

VL81: TCCACCTACCAGTTCACTTTGACTCAGCCATCCAGCGCGTCA---GTAGCTCCAGGACAAACTGTCAAACTGGCATGCGTTACAAGCAGTGGAAGCAGCATCACTGATCGTGTTGTACGCTGGTACCAGCAGAAGCCAGGGGAGAAGCCAAGATATCTGCTG

VL82: CCCAGTTTCCAGCAACTCCTTTCCGTACAGAGCTCTGCGTCA---GTGTCTCTTGGAGGGACCGTAACTCTCTCCTGCAGCCAGAGCAGTGGAGCCATCACCACTGTGAGCCATGCACACTGGATTCAGCAGAGACTGGGGCATGCGCCTCGTC---GACTG

VL83: AGCAGCACAAAGGCAAAACTAACCCAGCCGAGCTCTGTGCTG---GTGTCCCCAGGAGGCACTGCTAGCCTGAGCTGCACCACGAGTGA--GGA----CATCACTGGCTACACCATATCTTGGCTTCAGCAGAAATTTCAGAATCCTCCCAAGTACCTGCTG

VL84: TCTGGTGCCCAGAAGGTGGTGACACAGCAGGCTTTGGTATCA---GGGTCTCTGGGGCAGTCTATGAAACTCTCTTGCAGTGTAAGTGGAGGCTACAGTATCAGTAGCTATGGAATAGGGTGGTTCCAGCAGACCTCTGGGAACCCTCCCAGATCCCTGCTC

VL85: TGCTATGCTCAG------GTGACTCAGCCACCCACAGCATCC---GTGTCTCCAGGAGGGACGGTCCGACTCTCCTGCACGTTAGGGGGCAGTTACACTGTCAGTAGCAACCGTGTGCTTTGGTTACAGCAGAAGCCGGGGAATGCCCCCAGGTTCCTCCTG

VL86: TGCTATGCTCAG------GTGACTCAGCCACCCACAGCATCC---GTGTCTCCAGGAGGGACGGTCCGACTCTCCTGCACGTTAGGGGGCAGTTACACTGTCAGTAGCAACCGTGTGCTTTGGTTACAGCAGAAGCCGGGGAATGCCCCCAGGTTCCTCCTG

* 180 * 200 * 220 * 240 * 260 * 280 * 300

VL1 : TATTACAAGTCGGATTCTGACAAGCACCAGGGCTCCGGGGTCCCCGCTCGCTTCTCTGGCTCCAAAGACACGTCCAGTAACACCGGCTACTTGACCATCGCCGGGGTCCTCACAGAGGACGAGGCTGACTATTACTGT-- : 297

VL2 : ATGTACAAGGATAAAGAGA---------GGCCCTCTGATGTCCCGGACCGGTTCTCTGGAGCCA------GCTCTGGTGACACAGCCACCTTGACCATCACAGGAGTCCAAGCCCAGGACGAGGCGAACTATTACTGC-- : 270

VL3 : AGATACAAGTCCAATTCTGACAAGCACCAGGGCTCCGGGGTCCCCGCTCGCTTCTCTGGCTCCAAAGACACGTCCAGTAACACTGGCTACCTGACCATCGCCGGGGTCCTCACAGAGGACGAGGCTGACTATTACTGT-- : 297

VL4 : ATGTACAAGGATACAGAAA---------GGCCCTCCGATGTCCCTGACCGATTCTCTGCGGCCA------GCTCAGGTACCACACTAACACTGACCATCACTGGAGTCCAAGCAGAGGATGAGGCTGATTATTACTGTCA : 272

VL5 : TATTACAAGTCAGATTCTGACAAGCACCAGAGCTCCGGGGTCCCCGCTCGCTTCTCTGGCTCCAAAGATACGTCCAGTAACACCGGCTACCTGACCATCGCCAGGGTCCTCGCAGAGGACGAGGCTGACTATTACTGT-- : 297

VL6 : TATTACAAGTCGGATTCTGACAAGTACCAGGGCTCCGGGGTCCCCGCTCGCTTCTCTGCTTCCAAAGACACGTCCAGTAACACTGGCTACCTGACCATCGCCGGGGTCCTCGCAGAGGACGAGGCTGACTATTACTGT-- : 297

VL7 : ATATACAAGGATAAAGAGA---------GACCCTCCGATGTCCCTGACCAATTCTCTGGGGCCA------GCTCCGGTACCACAGCCACGCTGACCATCACTGGAGTCCAAGCACAGGACGAGGCTGATTATTACTGT-- : 270

VL8 : ATGTACAAGGATACAGAAA---------GGCCCTCCGATGTCCCTGACCGATTCTCTGCGGCCA------GCTCAGGTACCACACTAACACTGACCATCACTGGAGTCCAAGCAGAGGATGAGGCTGATTATTACTGT-- : 270

VL9 : TATTACAAGTCGGATTCTGACAAGCACCAGGGCTCCGGGGTCCCCGCTCGCTTCTCTGGCTCCAAAGACACGTCCAGTAACACCGGCTACTTGACCATCGCCGGGGTCCTCGCAGAGGACGAGGCTGACTATTACTGT-- : 297

VL10: ACATACAAGTCAGAGTCTCAAAAGTTCCCCAGCTCTGGGCTCCCCGCTCGATTCTCTGGCTCCAAAGGCACGTCCAGGAATGCTGCATATCTAACCATCGCTGGGGCCCTGGCAGAGGATGAGGCTGTTTATTACTGT-- : 297

VL11: ATATATGAGAATAGCAAAA---------GACCCTCCGGCATCCCTGACCGATTCTCTGGTGCCA------AATCTGGTGACACGGCCACGTTGACCATCACTGGAGTCCAAGCCCAGGACGAAGCAGATTATTACTGC-- : 270

VL12: ATATATAGTGATAGCAACA---------GACCCTCCGGCATCCCTGACCGATTCTCTGGTGCCA------ACTCCGGCAACACGGCCACATTGACCATCACTGGAGTCCAAGCTCAGGATGAGGCTGACTATTACTGC-- : 270

VL13: TATTACAAGTCGGATTCTGAGAAGCACCAGGGCTCCGGGGTCCCCGCTCGCTTCTCTGGCTCCAAAGACACATCCAGTAACACCGGCTACCTGACCATCGCCGGTGCCCTTGCGGAGGACGAGGCTGACTATTACTGC-- : 297

VL14: ATATATAGTGATAGCAAGA---------GACCCTCCGGCATCCCTGACCGATTCTCTGGTGCCA------ACTCCGGCAACACGGCCACATTGACCATCACTGGAGTTCAAGCCCAGGACGAAGCTGATTATTATTGTG- : 271

VL15: TATTACAAGTCGGATTCTGACAAGCACCAGGGCTCCGGGGTCCCCGCTCGCTTCTCTGGCTCCAAAGACACGTCCAGTAACACCTGCTACCTGACCATCGCCGGTGCCCTTGCGGAGGACGAGGCTGACTATTACTGT-- : 297

VL16: ATGTATAGTGATAGCAAGA---------GACCCTCCGGCATCCCTGACCGATTCTCTGGTGCCA------ACTCCGGCAACACGGCCACATTGACCATCACTGGAGTTCAAGCCCAGGACGAAGCTGATTATTATTGT-- : 270

VL17: ATATATAACGATGATAGCA---------GACCCTCTGGCATCCCTGACCGATTCTCTGGTGCCA------ACTCTGGCAACACAGCCACGTTGACCGTCACTGGAGTCCAAGCCCAGGACGAGGCTGAATATTACTGC-- : 270

VL18: ATATATGATGATACTAAGA---------GACCCTCTGGCATCTCTGACCGATTCTCTGGCTCCA------AATCTGGAAGCACGGCCACGCTGACCATCACTGGAGTCCAGGCCCAGGACGAGGCTGATTATTACTGC-- : 261

VL19: TATTACAAGTCGGATTCTGACAAGCACCAGGGCTCCGGGGTCCCCGCTCGCTTCTCTGGCTCCAAAGACACATCCAGTAACACCGGCTACCTGACCATCGCCGGGGTCCTTGCGGAGGATCAGGCTGACTATTACTGC-- : 297

VL20: ATATATGATAATACTAAGA---------GACCCTCCGGCATCTCTGACCGATTCTCTGGCTCCA------AATCTGGAAACACAGCCACGTTGACCATCACTGGAGTCCAGGCCCAGGACGAGGCTGACTATTACTGC-- : 261

VL21: ATATATAACGATGATAGCA---------GACCCTCTGGCATCCCCGACCGATTCTCTGGTGCCA------ACTCTGGCAACATGGCCACATTGACCATCACTGGAGTCCAAGCCCAGGACGAGGCTGAATATTACTGC-- : 270

VL22: ATATATGGTAATACTAACA---------GACCCTCCGGCATCTCTGACCGATTCTCTGGCTCCA------AATCTGGGAGCACGGCCACGCTGACCATCACTGGTGTCCAGGCCCAGGACGAGGCTGACTATCACTGC-- : 261

VL23: AGATACAAGTCGGATTCTAACAAGCACCAGGGCTCCGGGGTCCCCGCTCGCTTCTCTGCTTCCAAAGACACGTCCAGTAACACCTGCTACCTGACCATTGCCGCTGCCCTTGCAGAAGACGAGGCTGACTATTACTGT-- : 297

VL24: ATATATAATGATAGCAAGA---------GACCCTCCGGCATCCCTGACCGATTCTCTGGTGCCA------ACTCCGGCAACACGGCCACATTGACCATCACTGGAGTCCAAGCTCAGGATGAGGCTGACTATTACCGC-- : 270

VL25: ATATATGGTAATACTAACA---------GACCCTCCGGCATCTCTGACCGATTCTCTGGCTCCA------AATCTGGAAGCACGGCCACGCTGACCATCACTGGTGTCCAGGCCCAGGACGAGGCTGACTATTACTGC-- : 261

VL26: ATCTATCAGGACAGCAAGA---------GACCCTCCGGCATCCCTGACCGATTCTCTGGTGCCA------AATCTGGTGACACGGCCACGTTGACCATCACTGGAGTCCAGGCCCAGGATGAGGCTGATTATTACTGC-- : 270

VL27: ATCTATCAGAACAGCAAGA---------GACCCTCCGGCATCCCTGACCGATTCTCTGGTGCCA------ACTCTGGCAACACGGCCACGTTGACCATCACTGGAGTCCAGGCCCAGGATGAGGCTCATTATTACTGC-- : 270

VL28: ATATATGCTGATGATAGCA---------GAGCTGATGAAATCCCAGAGCGATTCTCTGGCGCCA------AGTCTGGGAACACGGCCACACTGACCATCACTGGAGTCCAAGCCCAGGACGAGGCTGATTATTACTGT-- : 270

VL29: ATATATGCTGATGATAGCA---------GAGCTGATGAAATCCCAGAGCGATTCTCTGGTGCCA------AGTCTGGGAACACGGCCACACTGACCATCACTGGAGTCCAAGCCCAGGACGAGGCTGATTATTACTGT-- : 270

VL30: GTATACTACGATGACAGAA---------AAGCTGATGGAATCCCTGACCAATTTTCTGGTTCCA------AGTCTGGGAGCACGGCCACGTTGACCATCACTGGACTCCAAGCCCAGGATGAAGCTGACTTTTATTGC-- : 270

* 180 * 200 * 220 * 240 * 260 * 280 * 300

VL31: ATATATATTGATGATAGCA---------GAGCTGATGAAATCCCAGAGCGATTCTCTGGTGCCA------AGTCTGGGAACATGGCCACACTGACCATCACTGGAGTCCAAGCTCAGGACGAGGCTGATTATTACTGT-- : 270

VL32: ATATATGACAATAGCAAGA---------GACCCTCTGGCATAGCCGATCGATTCTTTGGTTCAA------AATCCGGAAATACGGCCACATTGACCATCAGTGGAGTCCAAGCCCAGGATGAGGCTTATTACTACTGC-- : 270

VL33: ATATATGGGGATACTGAAA---------GGCCCTCGGGGATCCCCAGCCGATTCTCAGGGTCCATTGATAAATCTGCCAACACGGTCACGTTAACCATCAGTGGAGCGCAGCCGGACGATGAGGCTGATTATTACTGT-- : 282

VL34: GTGGACAGAAGTAATAACA---------GAGCCTCCGGTATCTCAGACCGGTTCTCTGGTTCCA------AGTCCGGTAACACGGCCACATTGACCATCACTGGAGTCCAAGCCCAGGATGAGGCTGATTATTATTGT-- : 270

VL35: ATGGGCAACGACAATAAGA---------GACCCTCCGGTATCTCGGACCGGTTCTCTGGTTCCA------AGTCCGAGAACACGGCCACATTGACCATCACTGGAGTCCAAACCGAGGACGAGGCTGATTATTACTGC-- : 270

VL36: ATATATGAGAATACCAAGC---------GACCCTCCAGCATCCCTGACCGATTCTCTGGTGCCA------GATCTGGGAACACGGCCACATTGACCATCACTGGAGTCCAGGCCCAGGACGAGGCTGATTATTACTGT-- : 267

VL37: ATATATGAGAATACCAAGC---------GACCCTCCAGCATCCCTGACCGATTCTCTGGTGCCA------GATCTGGGAACACGGCCACATTGACCATCACTGGAGTCCAGGCCCAGGACGAGGCTGATTATTACTGT-- : 267

VL38: ATGAAATATGATAATACCA---------GACCCTCCGGTATCTCAGACCGGTTCTCTGGTTCCA------AGTCCGGTAACACGGCCACATTGACCATCACTGGAGTCCAAGCTGAGGATGAGGCTGATTATTATTGC-- : 270

VL39: ATGTACAATAATAATAACA---------GACCCTCCGGTATCTCGGACCGGTTCTCTGGGTCCA------AGTCCGGTAACACGGCCACATTGACCATCACTGGAGTCCAAGCCCAGGACGAGGCTGATTATTACTGCAT : 272

VL40: ATGGACAGTAATAATAAGA---------GACCCTCTGGTATCTCGGACCGGTTCTCTGGGTCCA------AGTCCGGTAACACGGCCACATTGACCATCACTGGAGTCCAAGTCCAGGACGAGGCTGATTATTACTGC-- : 270

VL41: ATATATAGTGACAGCAACA---------GACCCTCCGGCATCTCTGACTGATTTTCTGGCTTCA------AATCGGGGAGCACAGCCACGTTGACCGTCACTGGAGTCCAAGCCCAGGATGAGGCTGATTATTACTGC-- : 270

VL42: ATATACGAAAATAGCAAAA---------GACCCTCTGGCATTGCTGACCGGTTCTCTGGCTCCA------AATCAGAGAGCACGGCCACATTGACCATCACTGACATCCAAGCCCAGGACGAGGCTGACTATTACTGC-- : 270

VL43: ATATATGATAATAGCAACA---------GACCCTCTGGTATTGCTAACCAATTCTCTGGCTCCA------AATCAGGGAGCACGGCCACATTGACCATCACTGGAGCCCAAGCCCAGGACGAGGCTGATTATTACTGT-- : 270

VL44: ATATATAGCAATAGCAACA---------GACCCTCCAGCATCCCTGACCAATTCTCTGCTTCCA------TATCTGGAAACACGGCCACATTGAGCATCACTGGTGTCCAAGCCCAGGATGAGGCTGATTATTACTGC-- : 270

VL45: GCATACAAATCCGATTCTGACAAGCGCCAGGGCCCTGGGGTCCCCGCTCGCTTCTCTGCCTTCAAAGACACATCCATTAACACCTGCTATCTAACCATCACTGGTGCCCCGGCAGAGGATGAGGCTAATTATTACTGT-- : 297

VL46: ATGTACAATAGTAGGAGCA---------GACCCTCCGGCATCCCTGACCGATTCTCTGGTGCCA------GCTCTGGGAACATGGCCACGTTGACCATCACTGGCGTCCAAGCCCAGGACGAGGCGGTTTATTACTGT-- : 270

VL47: TGGTACAATACAGACTCTAGCAAGCACCAGGGCTCCGGGGTCCCGAGTCGCTTCTCTGGTTCCAAAGACACCTCTAATAAAATTGGCTACTTAACCATCACCAGTGTCCAGGCAGAGGATGAGGCTGATTATTACTGT-- : 291

VL48: ATATACAGCAACAACAGCA---------GGCCCTCGGGGATCCCCACCCGGTTCTCTGGGTCCA------TATCTGGCCAGAAGGCCACTTTGACCATCACTGGGGTCCAAGCCGAAGATGAGGCCGACTACTACTGT-- : 282

VL49: ATGTACAATAGTAGGAGCA---------GACCCTCCGGCATCCCTGACCGATTCTCTGGTGTCA------GCTCTGGGAACACGGCCGTATTGACTATCATCGGTGTCCAAGCCCAGGACGAGGCGGTTTATTACTGT-- : 270

VL50: ATGTTCAATACGGACTCTGACAAGCACCAGGGCTCCGGGGTCCCCAGTCGCTTCTCTGGTTCCAAAGACACCTCGAATAAAATTGGCTACTTAACCATCACCAACGCCCAGGCAGAGGATGAGGCTGATTATTATTGC-- : 291

VL51: ATGTACAGCAATAATAACA---------GACCCTCCGGAATCTCGGACCGGTTCTCTGGTTCCA------AGTCCGGTAACACGGCCACATTGACCATCACTGGAGTCCAAGCCCAGGACGAGGCTGATTATTACTGC-- : 270

VL52: ATATACAGCGACAGCATCC---------GAGCCTCAGGCATCTCTGACCAACTCTCTGGCTCTA------GATCTGGGAACACGGCCACATTGACCATCACTGGAGTCCAAGCCCAGGATGAGGCTGACTATTACTAC-- : 270

VL53: ATATACGAAAACAGCAAAA---------GACCCTCTGGCATCGCTGACCGGTTCTCTGGCTCCA------AATCAGGGAGCACGGCCACATTGACCATCACTGGAGCCCAAGCCCAGGATGAGGCTGATTATTACTGC-- : 270

VL54: ATGTACAGCACTAATAACA---------GACCCTCCGGTATCTCGGACCGGTTCTCTGGTTCCA------AGTCAGGTAACACGGCCACATTGACCATCACTGGAGTCCAAGCCCAGGACGAGGCTGATTATTACTGC-- : 270

VL55: ATATACAGCGACAGCATCC---------GAGCCTCAGGCATCTCTGACCGACTCTTTGACTCCA------AATCTGGGAACACGGCCACATTGACCATCACTGGAGTCCAAGCCCAGGACAAGGCTGACTATTACTAC-- : 270

VL56: GTGTATTACACCAGCAACA---------GGCCCTCCAGGATCCCCACCCGGTTCAATGGGTCCA------TATCTGGCCAGAAGGCCATCTTAACTATCATGGGAGTCCAAGCCGAGGATGAGGCTGACTACTACTGTGC : 284

VL57: TGGTACAGTTCAGGTACCA---------AAGGAGATGGAGTCTCAGATCGATTCACTGTCTCCAG-----C-TCCGGTGCCATTCGCTATCTAACCATCACCAACGCCCGAGCAGACGATGAGGCGACTTACTGGTGT-- : 273

VL58: TGGCACAGCGAAGGGATCA---------AAGGGAATGGAGTCCCAGATCGATTCAGCGTTTCCAA-----C-TCCAGCGCCATACGCTATTTAACCATCACCAATGTCCCTGGAGAAGATGAGGCCATTTACTGGTGTGG : 275

VL59: TGGTACAATACAGACTCTAGCAAGCACCAGGGCTCCGGGGTCCCGAGTCGCTTCTCTGGTTCCAAAGACACCTCTAATAAAATTGGCTACTTAACCATCACCAGTGTCCAGGCAGAGGATGAGGCTGATTATTATTGT-- : 291

VL60: TGGTACAGCAAAGGGACCA---------AAGGGGATGGAGTCCCAGATCGATTCACTGTTTCAAA-----C-TCCAGTGCCATTCGCTCTCTGACCATCACCAATGTCCAAGCTGATGATGAAGCCACTTACTGGTGTGG : 275

VL61: ATATACAACACCAACAGCA---------GGCCCTCGGAGATCCCCGCTCGGTTCTCGGGGTCCA------TATCTGGTCAGAAGGCCACCTTGACCATCACGGGGGTCCAAGCTGACGATGATGCAGATTATTACTGT-- : 279

VL62: ATGTCTAACAACAACACCA---------GGCCCTCCGGGATCCCCACCCGGTTCTCAGGGTCCA------TTTCCGGTCAGAAGGCCGTATTAACCATCACTGGGGCCCAGCTCGAGGATGACGCTGATTATTACTGT-- : 279

VL63: ATATACAGCACCAACAGCA---------GGCCCTCCGGGATCCCCACCCGGTTCTCGGGGTCCA------TATCTGGTCAGAAGGCCGTATTAACCATCACGGGGGTCCAAGCAGAGGATGAGTCCGACTACTACTGTCT : 281

VL64: ATATATGAGGATACTAAAA---------GGCCCTCGGGGATCCCCAGCCGATTCTCAGGGTCCATTGATGAATCTGCCAACACGGCCACGTTAACCATCAGTGGAGCGCAGCCGGACGACGAGGCTGATTATTACTGTTT : 284

VL65: ATATACCGCACCAACAACA---------GGCCCTCGGGGATCCCCACCCGGTTCTCAGGGTCCA------TATCTGGTCAGAAGGCCGCCTTGACCATCACGGGGGTCCAAGCCGAGGATGAGGCTGATTACTACTGTAT : 281

VL66: ATGTACTACGCCAGCAGCA---------GGCCCTCGGGGATCCCCACCCGGTTCTCGGGGTCCA------TTTCTGGTCAGAAGGCTGCCTTGACCATCACGGGGATTGAAGTTGAGGATGATGCAGATTATTACTGT-- : 282

VL67: ATATATGAGGATACTGAAA---------GGCCCTCGGGGATCCCCAGTCGATTCTCAGGGTCCATTGATAAATCTGCCAACACGGCCACGTTAACCATCAGTGGAGCGCAGCCGGACGACGAGGCTGATTATTACTGTTT : 284

VL68: ATATATGACACCATGACCA---------GGCCCTCTGGGCTCCCCACCCGGTTCTCGGGGTCCA------TCTCTGGTCAGAAGGCCGCTTTAACCATCACACGGGTCCAAGCCGAGGATGACGCCGACTACTACTGTGT : 281

VL69: ATATACAGCACCAACAGCA---------GGCCCTCGGGGATCCCCACCCGGTTCTCGGGGTCCA------TACCTGGTCAAAATGCCGCCTTGACCATCACAGAGGCTCAAGCCGAGGATGAGGCTGACTACTACTGTAT : 281

VL70: ATATACAGCACCAGTACCC---------GGCCCTCTGGTGTGCCAGCACGCTTCTCTGGGTCCC------GATCAGGGAACGCCATGTCCTTGACCATCACTGGGGCCCAGCCCGAGGATGATGCTGATTATTACTGT-- : 279

VL71: ATGTACAGCACCAGTACCC---------GGCCCTCTGGTGTGCCAGCGCGCTTCTCTGGGTCCC------GATCAGGGAGCACCATGTCCTTGACCATCACTGGGGCCCAGCCCGAGGATGACGCTGATTATTACTGTTC : 281

VL72: ATATACAACACCAACAGCA---------GGCCCTCAGAGATCCCCACCCGGTTCTCAGGGTCCA------TATCTGGTCAGAAGGCCACCTTGACCATCACGGGGGTCCAAGCCGAGGATGAGGCTGACTACTACTGTAT : 281

VL73: ATATACCTCACCAGCAGCA---------GGCCCTCGGGGATCCCCACCCGGTTCTCGGGGGACA------TTTCTGGTCAGAACAACACCTTAACCATTATGGCAGTCCAAGCCGAGGATGAGGCTAACTACTTCTGTGG : 281

VL74: ACTTATGAAAGTAATAAGA---------GACCCTCTGGGGTCCCCACCCGGTTCTCCGGGTCCA------TCTCTGGTCAGAAGGCCGCCTTAACCATCACCGGGGCCCAAGCTGAGGACGAGGCCGACTACTACTGT-- : 282

VL75: ATATACAGCACCAGCAGCA---------GGCCCTCGGGGATCCCCACCCGGTTCTCTGGGTCCA------TATCTGGTCAGAAGGCCGTCTTGACCATCACGGGGGTCGAAGCCGAGGATGAGGCTGACTACTACTGT-- : 279

VL76: ATTTATGAAAGTAATAAGA---------GACCCTCTGGGGTCCCCACCCGGTTCTCCGGGTCCA------TCTCTGGTCAGAAGGCCGCCTTAACCATCACCGGGGCCCAAGCTGAGGACGAGGCCGACTACTACTGT-- : 282

VL77: ATTTATGAAATTAGTAAGA---------AACCCTCTGGGGTCCCCACCCGGTTCTCGGGGTCCA------TTTCTGGCCAGAAGGCCACCTTGACCATCACTGGGGCCCAGCCTGAGGATGACGCTGATTATTACTGT-- : 282

VL78: ATATACAGCACCAGTACCC---------AGCCCTCTGGTGTGCCAGCGCGCTTCTCTGGGTCCC------GATCAGGGAGCACCATGTCCTTGACCATCACTGGGGCCCAGCCCGAGGATGACGCTGATTATTACTGT-- : 279

VL79: ATATATAACACCGACAGTA---------GGCCCTCGGGGATCCCCACCCGGTTCTCGGGGTCCA------TATCTGGTCAGAAGGCCGTATTAACCATCAAGGGAGTCCAAGCCGAGGATGAGGCTGACTACCACTGTAT : 281

VL80: ATTTATGAAATTAGTAAGA---------AACCCTCTGGGATCCCCACCCGGTTCTCGGGGTCCA------TATCTGGCCAGAAGGCCGCCTTGACCATCGCGGGGGTCCAAGCCGAGGATGAGGCTGACTACTACTGTGC : 281

VL81: TATTACAGGGATGAATCTACAAACCACAAAGGCTCTGGGGTCCCCAATCGGTTTTCAGCTTCTAAAAGCACTTCTACGAACACCTGCTTCTTAACCATTGCCCAGGTCCAAGCAGAGGATGAGGCTGAGTACTACTGT-- : 297

VL82: ATATATGGTGCCAGTGATA---------GAGGACCTGGAGTCCCAGAGAGGTTCACAGCTTCCA------TCTCTGCTAGCACAATGTCCTTGACCATTGCTGGGGCCCAAGCAGAAGATGAGGGTGCTTATTATTGT-- : 279

VL83: TATTATAAAGATGAAGCTAAACAAGGCAAAGTCTCTGGGGTCCCCACTCGTTTCTCTGCTTCTAAAGACACCTCCAGCAGCACCTGCTATTTAACCATTTCGGGGGCCTTGGCAGAAGATGATGCTGTTTATTACTGT-- : 291

VL84: TACTACTACAGTGTTTCGAGAACAGGCAGAGGCTCTGGGGTCCCAGACCGGCTCTCAGGCTCTGCCTCTGG------CAGCGTTGGTTATTTAACCATCTCTGGGCTCCAAGCAGAGGATGATGCTGATTATTATTGT-- : 291

VL85: TATTTTTTCACTGAGTCTAATAAAGGCATGGGCTCCGGGGTTCCCAGCCGGTTCTCTGGCTCTCGCTCTGGTTCAGACAAGGAAGGCTATTTAACAATCTCTGGGGCCGTGGAAGAGGATGATGCTGATTATTACTGTGC : 293

VL86: TATTTTTTCACTGAGTCTAATAAAGGCATGGGCTCCGGGGTTCCCAGCCGGTTCTCTGGCTCTCGCTCTGGTTCAGACAAGGAAGGCTATTTAACAATCTCTGGGGCCGTGGAAGAGGATGATGCTGATTATTACTGTGC : 293
