# Supplementary material for: A Comprehensive Analysis of the Phylogeny, Genomic Organization and Expression of Immunoglobulin Light Chain Genes in Alligator sinensis, an Endangered Reptile Species
Source: PLoS One. 2016 Feb 22;11(2):e0147704. doi: 10.1371/journal.pone.0147704 (PMC4762898; doi:10.1371/journal.pone.0147704)
Supplement: S2 Appendix — (DOCX) [file pone.0147704.s002.docx]

**The *Alligator sinensis* V_λ_ gene DNA segment in contigs**

| \| **Name** \|  \|  \| \| --- \| --- \| --- \| | **V-region position in contig** | **RSS** | **Defects in V_λ_ pseudogenes and ORFs** |
| --- | --- | --- | --- | --- | --- | --- |
| V1 | BAC Y127H24 (48957-49279) | CACAGTGACACAGCCACAGGGGAAGTGAGACAAAAACC |  |
| V2 | BAC Y127H24 (46479-46780) | CACAGTGACACAGGGCAGTGGGGAAGTGAGACACAAACT |  |
| ΨV1 | BAC Y127H24 (43758-44064) |  | One in frame stop codon in FR3, and absence of RSS. |
| ΨV2 | BAC Y127H24 (39092-39353) | CACAGTGACACAGGGCAATGGGGAAGTGAGACACAATTT | Absence of AG splicing signal, and unusual nonamer of RSS |
| ΨV3 | BAC Y127H24 (37644-37880) |  | One in frame stop codon in FR3, and no RSS. |
| ΨV4 | BAC Y127H24 (33870-34110) | TACAGTGACACAGGGCAATGGGGAAGTGAGACACAAACT | No YYC in FR3, no splicing signal in leader peptide, one in frame stop codon in FR3. |
| V3 | BAC Y127H24 (31866-32188) | CACAGTGACACAGCCACAGGGGAAGTGAGACAAAAACC |  |
| ΨV5 | BAC Y127H24 (30743-30914) |  | Deletion of FR1 and CDR1, and absence of RSS. |
| ΨV6 | BAC Y127H24 (26808-27068) |  | Two in frame stop codons in FR3, and absence of RSS. |
| V4 | BAC Y127H24 (25968-26280) | CACAGTGCCACAGGGCAATGGGGAAGTGAGAGACAAACT |  |
| ΨV7 | BAC Y127H24 (24488-24756) | CACAGTGGCACTGTGCAATGGGGACGTCAGACCCAACCC | Two in frame stop codons in FR1, and four in frame stop codons in FR3. |
| V5 | BAC Y127H24 (15959-16266) | CACAGTGATGCTTGTCACAGTGACACAGCCACAGGGGAA |  |
| ΨV8 | BAC Y147P18 (97464-97737) | CACAGGGGAACAGTGCAATGGGGACATGAAACATAACCC | Two in frame stop codons in CDR1 and CDR2 respectively. |
| V6 | BAC Y147P18 (90402-90724) | CACAGTGACACAGCCACAGGGGAAGTGAGACAAAAACC |  |
| ΨV9 | BAC Y147P18 (88809-89076) | CACAGGGAAGTCAGACCAACCCCTCCTCTGCCCTCAAAG | Two in frame stop codons in FR2 and FR3 respectively, and unusual nonamer of RSS |
| V7 | BAC Y147P18 (81400-81698) | CACAGTGACACCGGGCAATGGGGAAGTGAGACACAAACT |  |
| ΨV10 | BAC Y147P18 (74350-74621) |  | Absence of splicing signal and RSS. |
| V8 | BAC Y147P18 (72891-73204) | CACAGTGCCACAGGGCAATGGGGAAGTGAGACACAAACT |  |
| ΨV11 | BAC Y147P18 (69992-70283) | CACAGTGGCACTGTGCAATGGGGACGTGAGACCCAGGCT | Two in frame stop codons in FR1, one in frame stop codon in CDR2. |
| V9 | BAC Y147P18 (68426-68748) | CACAGTGACACAGCCACAGGGGGAAGTGAGACAAAAACC |  |
| ΨV12 | BAC Y147P18 (65883-66131) |  | Two in frame stop codons in FR2 and FR3 respectively, and absence of RSS. |
| ΨV13 | BAC Y147P18 (61062-61381) |  | Two in frame stop codons in CDR1 and CDR2 respectively, and absence of RSS. |
| ΨV14 | BAC Y147P18 (56998-57285) | CACAGTGACATGATGCAATGGGGAAGTGAGATACAGATC | One in frame stop codon in CDR2, and unusual nonamer of RSS. |
| V10 | BAC Y147P18 (51588-51895) | CATAACGACGCTCAACACAGCAGTTCAGTGAGACAAAAA |  |
| V11 | BAC Y147P18 (48935-49230) | CACAGTCACACAGTGCAATGGGGAAGTGAGACACAAACG |  |
| V12 | BAC Y147P18 (47217-47518) | CACAGTGATACAGGGCAATGTGGAAGTGAGACACAAACA |  |
| V13 | BAC Y147P18 (41294-41616) | CACAGTGATCTAGTCAGAGGGGAAGTGAGACAAAAATC |  |
| V14 | BAC Y47P24 (112451-112746) | CCCAGTGACACAGGGCAATGGAGAAGTGAGATAGAAACC |  |
| V15 | BAC Y47P24 (100219-100541) | CACAGTGATCTAGTCAGAGGGGAAGTGAGACAAAAACC |  |
| ΨV15 | BAC Y47P24 (96018-96325) | CACAGCACATCAGTCCACTGGAGAATTGAGACAAAAACC | One in frame stop codon in FR2. |
| V16 | BAC Y47P24 (92751-93046) | CCCAGTGACACAGGGCAATGGAGAAGTGAGATAGAAACC |  |
| ΨV16 | BAC Y47P24 (89110-89303) |  | No YYC in FR3, and no RSS. |
| V17 | BAC Y47P24 (87474-87775) | CACAGTGACACAGGGCAGTGTGGAAGTGAGACACAAACA |  |
| V18 | BAC Y47P24 (83537-83829) | CACGGTGACACAGTGCAATGAAGAAGTGAGACATAAACC |  |
| V19 | BAC Y47P24 (79316-79638) | CACAGTGATCTAGTCAGAGGGAAAGTGAGACAAAAACC |  |
| ΨV17 | BAC Y47P24 (72644-72938) | CCCAGTGACACAGAGCAATGGACAAGTGAGACAGAAAGC | No signal peptide. |
| ΨV18 | BAC Y47P24 (68988-69243) |  | One in frame stop codon in FR1 and no RSS. |
| ΨV19 | BAC Y47P24 (67213-67407) | CACAGTGACACAAAGCAGTGTGGAAATGAGACACAAACA | Absence of FR1 and CDR1, and one in frame stop codon in CDR3 . |
| V20 | BAC Y47P24 (65121-65413) | CACGGTGATACACTGCAATGGAGAAGTGAGACATAAACC |  |
| ΨV20 | BAC Y47P24 (60877-61069) |  | No YYC in FR3, one in frame stop codon in FR2, and no RSS. |
| ΨV21 | BAC Y47P24 (59889-60051) |  | No YYC in FR3 and absence of RSS. |
| V21 | BAC Y47P24 (59241-59542) | CACAGTGACACAGGGCAGTGTGGAAGTGCGACACAAACA |  |
| V22 | BAC Y47P24 (55378-55670) | CACGGTGACACAGTGCAATGAAGAAGTGAGACATAAACC |  |
| V23 | BAC Y47P24 (44478-44800) | CACAGTGATATAGTGAGAGGGGAAGTGAGACAAAAACG |  |
| V ORF1 | BAC Y47P24 (41344-41665) | CACAGCACATCAGTCCACTGGAAAAGTGAGACAAAAACC |  |
| V24 | BAC Y47P24 (38816-39111) | CACAGTCACACAGTGCAACGGGGAAGTGAGACACAAACG |  |
| ΨV22 | BAC Y47P24 (33605-33812) |  | No YYC in FR3 and absence of RSS. |
| ΨV23 | BAC Y47P24 (29068-29341) |  | Two in frame stop codons in FR2 and FR3 respectively, and absence of RSS. |
| V25 | BAC Y47P24 (98864-99156) | CACGGTGATACACTGCAATGGAGAAGTGAGACATAAACC |  |
| ΨV24 | BAC Y47P24 (94145-94411) |  | Two in frame stop codons in FR1 and FR3 respectively, and absence of RSS. |
| ΨV25 | BAC Y213O3 (89083-89405) | CACAGTGATACAGTCAGAGGAGAAGTGAGACAAAAACC | No leader peptide. |
| V26 | BAC Y213O3 (85191-85492) | CACAGTGACACAGTCAGATGGGAACTGAGACTCAAACC |  |
| ΨV26 | BAC Y213O3 (82341-82663) |  | No leader peptide and no RSS |
| V27 | BAC Y213O3 (80002-80303) | CACAGTGACACAGTCAGATGGGAACTGAGACTCAAACC |  |
| V28 | BAC Y213O3 (73569-73871) | CACAGTGACACATTGCAATGGGGAAGTGAGACACAAACC |  |
| ΨV27 | BAC Y213O3 (67900-68110) |  | Two in frame stop codons in FR2 and FR3 respectively, no YYC in FR3, and no RSS. |
| V29 | BAC Y213O3 (65666-65967) | CACAGTGACACATTGCAATGGGGAAGTGAGACACAAACC |  |
| V30 | BAC Y213O3 (61685-61986) | CACAGTGATGCAATGTGACAGGGAAGTGAGAAACAAACT |  |
| V31 | BAC Y213O3 (60244-60546) | CACAGTGACACAGTGTGATGTGGAAGTGAGAGATAAACC |  |
| V32 | BAC Y213O3 (52184-52485) | CACAGTGACACAGTGTAATGGGGAAGTGTGACACAAACC |  |
| V33 | BAC Y213O3 (49729-50024) | CACAGTGATTCAGACACATGAGGAACTGAGACAAAAACC |  |
| ΨV28 | BAC Y213O3 (47837-48080) |  | One in frame stop codon in FR3, and absence of RSS. |
| V34 | BAC Y213O3 (41583-41881) | CACAGTGACACAGTGCAATGGGGAAGTGCGACACAAACC |  |
| ΨV29 | BAC Y213O3 (30875-31176) | CACAGTGACACAATGCAATCAAGGTTGCCTTTAAGCTGC | One in frame stop codon in FR2, and unusual nonamer of RSS. |
| V35 | BAC Y213O3 (23998-24299) | CACAATGACACAGTGCAATGGGGAAGTGCAACACAAACC |  |
| ΨV30 | BAC Y213O3 (21723-21970) |  | Two in frame stop codons in FR2 and FR3 respectively, and no RSS. |
| ΨV31 | BAC Y213O3 (17107-17391) | CATAGTGACACAAGACAATGGGGAAGGGAGACACAAAGC | One in frame stop codon in FR3. |
| V36 | BAC Y213O3 (13241-13539) | CACAGTGACACAGAGCAATGGGGAAGTGAGACACAAACC |  |
| ΨV32 | BAC Y213O3 (6863-7164) | CACAGTGATACAGAGCAATGGGGAAGAGAGGCCGAAACC | One in frame stop codon in FR3. |
| V37 | BAC Y213O3 (4704-5005) | CACAGTGACACAGTGCAATGGGGAAGTGCGACACAAACC |  |
| V38 | KE698600.1 (1037-1335) | CACGATGACACAGTGCAATGGGGAAGTGTGACATGAACC |  |
| ΨV33 | AVPB01102472.1 (806-1110) |  | Two in frame stop codons in FR1 and FR3 respectively, and no RSS. |
| ΨV34 | KE698001.1 (24700-24900) |  | One in frame stop codon in FR2, and absence of RSS. |
| ΨV35 | KE698001.1 (3931-4205) | CACAGTGACAATGCAATGGGGAAGTGAGACACAAACCTC | One in frame stop codon in FR3, and unusual nonamer of RSS. |
| ΨV36 | KE698031.1 (20572-29873) | CACAGCGATACAGAGCAATGGGGAAGAGAGGCCCAAACC | One in frame stop codon in FR3, and a shorter leader peptide. |
| ΨV37 | KE698031.1 (19271-19550) |  | One in frame stop codon in FR3, and absence of and RSS. |
| ΨV38 | KE698031.1 (16006-16210) | CACAGTGCCACAGGGCAATGGGGAAGTGAGTCAAACATG | Deletion of FR1 and CDR1, absence of leader peptide, and unusual nonamer of RSS. |
| ΨV39 | KE698031.1 (3754-3461) |  | Absence of RSS. |
| V39 | KE697531.1 (116047-116348) | CACAATGACACAGTGCAATGGGGAAGTGCGACACAAACC |  |
| V40 | KE697531.1 (111287-111588) | CACAATGACACAGTGCAATGGGGAAGTGAGACAGAAACT |  |
| V41 | KE697531.1 (97387-97688) | CACAGTTATACAATGCAATGGGGATGTGAGACAAAAACC |  |
| ΨV40 | KE697531.1 (91029-91314) | CATAGTGACACAGGCAATGGGGAAGTGAGACACAAACC | One in frame stop codon in FR1, and absence of RSS. |
| V42 | KE697531.1 (82700-83001) | CACGGTGACACTGGGCAATGGGGAAGTGAGACACAAACC |  |
| V43 | KE697531.1 (73258-73559) | CACGGTGACACTCATTGACCATCACTGGAGCCCAAGCCC |  |
| V44 | KE697531.1 (64749-65050) | CACAGTGACACTGGGCAATGGGGAAGTGAGACACAAACC |  |
| ΨV41 | KE697531.1 (59778-600333) |  | Four in frame stop codons in FR3, no YYC in FR3, and absence of RSS. |
| V45 | KE697531.1 (55540-55862) | CACGGTGATGCAGGCTGATGGGGAAGTGAGACACAAACC |  |
| ΨV42 | KE697531.1 (49011-49221) |  | Two in frame stop codons in FR2 and CDR2 respectively, two in frame stop codons in FR3, no YYC in FR3, and absence of RSS. |
| ΨV43 | KE697531.1 (46450-46736) | CACAGTGATCCAAGTAGAAGGGGAAGCCAGGGAAAAACT | Two in frame stop codons in FR2 and CDR2 respectively, four in frame stop codons in FR3, absence of unusual nonamer of RSS. |
| V46 | KE697531.1 (43655-43956) | CACAGTGATACAGTCCGATGGGGAAGTACAACATAAACG |  |
| V47 | KE697531.1 (39437-39753) | CACAGTGACAGCTTTTGATGCAGATCCTGAACAAAAACT |  |
| ΨV44 | KE697531.1 (35082-35355) | TACAGTGGAAAGTGTCACAGTGCTGCGGATGGATGCAAA | One in frame stop codon in FR3, absence of leader peptide and unusual nonamer of RSS. |
| ΨV45 | KE697531.1 (31809-32082) |  | One in frame stop codon in FR3, absence of RSS. |
| V48 | KE697531.1 (25883-26196) | CATGGTGCTACACCTTGATGGGTAAGTGAAGCAAGAACC |  |
| ΨV46 | KE697531.1 (16868-17153) |  | Two in frame stop codons in CDR2 and FR3 respectively, absence of RSS. |
| V49 | KE697531.1 (7872-8173) | CACAGTGATACAGTCCGATGGGGAAGTACAACATAAACG |  |
| V50 | KE697531.1 (2712-3025) | CACAGTGACACAGTTAGTTGCAGACCTGAATAAAACCTT |  |
| V51 | KE697626.1 (83033-83334) | CACAGTGACACAGTGCAATGGGGAAGTGCGACACAAACC |  |
| V52 | KE697626.1 (79188-79489) | CACAGTGCCACAGGGCAGTGGGGAAATGAGCCACAAACA |  |
| V53 | KE697626.1 (68367-68668) | CACAGTGACACAGGGGAATGTGGATGGGAGACAGAAACC |  |
| ΨV47 | KE697626.1 (66136-66319) |  | Deletion of FR1 and CDR1, Two in frame stop codons in FR2 and FR3, and no RSS. |
| ΨV48 | KE697626.1 (64849-65114) |  | Absence of splicing signal, no YYC in FR3, and no RSS. |
| V54 | KE697626.1 (58401-58702) | CACAATGACGCAGTGCAATGGGGAAGTGCAACACAAACC |  |
| ΨV49 | KE697626.1 (54445-54746) | CACAGTGCCACAGGGCAATGGGGAAGTGAGACACAAACA | Absence of leader peptide. |
| ΨV50 | KE697626.1 (37632-37808) |  | Deletion of FR1, and no RSS. |
| V partial 1 | KE697626.1 (31053-31362) | CACAGTGCAATGGGGAAGTGCGACACAAACCTCCTGTTA |  |
| ΨV51 | KE697626.1 (14507-14685) |  |  |
| V55 | KE697626.1 (13653-13954) | CACAGTGCCACAGGGCAATGGGGAAGTGAGTAACAAACG |  |
| ΨV52 | KE697626.1 (12035-12336) | CACAGTGACACAGGCAATGGGGAAGTGAGACACAAACA | One in frame stop codon in FR2. |
| ΨV53 | KE697626.1 (2908-3150) |  | Absence of leader peptide and RSS. |
| V56 | KE695978.1 (3618382-3618694) | CACAGGGCTGCAGCTCGATGGGGAAGTGAGGCAAAAACC |  |
| V57 | KE695978.1 (3585940-3586250) | CACAGTGCCACAGTCAAATGGGGTACTGAGACAAAAACC |  |
| V58 | KE695978.1 (3578479-3578791) | CACTGTGACACAGTCAGATGACGAACTGAGACAAAAACC |  |
| ΨV54 | KE695978.1 (3540820-3540972) | CACAGCGACAAGAGACAATGGGGATGAGAAACACAAACC | Deletion of FR1 and CDR1, and one in frame stop codon in FR3. |
| V59 | KE695978.1 (3487795-3488111) | CACAGTGACAGCTTTTGATGCAGATCCTGAACAAAAACT |  |
| V60 | KE695978.1 (3473946-3474258) | CACCATGACACAGTCAGATGGGAACTGAGACAAAAACC |  |
| V61 | KE695978.1 (3456650-3456854) | CACAGTGATACAGTCAGATGGGGAAGTGTGACTAAAACT |  |
| V62 | KE695978.1 (3451859-3452163) | CACAGTGATACAGTCCAATGGGGAAGTGCGACAAAAATC |  |
| ΨV55 | KE695978.1 (3444246-3444435) | AACAGTGATACAGTCTGCTGGGAAAGCGCAACAAAACCT | Deletion of FR1 and CDR1, and one in frame stop codon in FR3. |
| V63 | KE695978.1 (3439319-3439628) | CACAGTGATAAACCCTGATGAGGAACTGAGACATAAACC |  |
| V64 | KE695978.1 (3432336-3432651) | CACAGTGCTACAGACACAAGGGGAACTGAGACAAAAACC |  |
| ΨV56 | KE695978.1 (3428214-3428504) | CACAGCGATAGAGCCTGATTGGAAACTAAGACATAAACC | Two in frame stop codons in FR3. |
| ΨV57 | KE695978.1 (3421578-3421799) |  | One in frame stop codon in FR2, no YYC in FR3, and absence of RSS. |
| V65 | KE695978.1 (3419314-3419620) | CACAGTGATAGAGCCCGATGGGGAACTGAGACATAAACC |  |
| V ORF2 | KE695978.1 (3413809-3414108) | CACAGGGACAGAGCCAAATGGGGAATTACAACCACTCCG |  |
| ΨV58 | KE695978.1 (3412617-3412926) | CACAGTGATAAACCCCAATGAGGAACTGAGACATAAACC | One in frame stop codon in the last five bases. |
| V66 | KE695978.1 (3409565-3409872) | CACAGTGATACAGTCAGATGGGGAAGTGTGACTGAAACT |  |
| V67 | KE695978.1 (3396293-3396602) | CACAGTGCTACAGACACAAGGGGAACTGAGACAAAAACC |  |
| V68 | KE695978.1 (3393683-3393992) | GACGGTGATAGAGCCCGATGAGGAACTGAGACATAAACC |  |
| V69 | KE695978.1 (3390068-3390374) | CACAGTGATAGAGCCCCATGGGGAACTGAGACATAAACC |  |
| ΨV59 | KE695978.1 (3383481-3383655) |  | Deletion of FR1 and CDR1, and one in frame stop codon in FR2, and absence of RSS. |
| ΨV60 | KE695978.1 (3377107-3377299) | CCCTGTGATATAGTCCAATGGGGAAGTGGGGAAAAAACT | Deletion of FR1 and CDR1, one in frame stop codon in FR2, three in frame stop codons in FR3, and unusual nonamer of RSS. |
| ΨV61 | KE695978.1 (3372712-3373039) | CAGAGTAGAAGAGCTGCACGAAGTGGAGGAACAAAAACT | Deletion of FR1 and CDR1, and no leader peptide. |
| V70 | KE695978.1 (3362199-3362503) | CACAGCAATGCTTTCAGATGGGGAAGTGAGACAAAAACC |  |
| ΨV62 | KE695978.1 (3358734-3359047) |  | One in frame stop codon in FR3, and absence of RSS. |
| V71 | KE695978.1 (3348873-3349176) | CACAGTGATACAGTCTTATGGGGAAGTGCAACAAAAACT |  |
| V72 | KE695978.1 (3332639-3332948) | CACAGTGATAGAGATGAATGGGGAACTGAGACATAAACT |  |
| V73 | KE695978.1 (3328147-3328459) | CACCGTGATAGCGCCCTATGAGTAACTGAGACAGAAACC |  |
| V74 | KE695978.1 (3324957-3325264) | CACAGTGACACAGTGATATGGGGAACTGCAACAAAAACT |  |
| V75 | KE695978.1 (3316906-3317222) | CACTGTGTTTCATTTTGATGAGGAACTGAGACACAAACC |  |
| V76 | KE695978.1 (3312908-3313215) | CACAGTGATAGAGCCCGATGGGGAACTGAGACATAAACC |  |
| V77 | KE695978.1 (3300771-3301087) | CACTGTGTTTCATTTTGATGAGGAACTGAGACACAAACC |  |
| ΨV63 | KE695978.1 (3291646-3291945) | ACACAAATGTAGAGCGTGACGGGAACGGAGCCATAAACC | Two in frame stop codons in FR3, and unusual heptamer of RSS. |
| ΨV64 | KE695978.1 (3289893-3290105) |  | Deletion of FR1 and CDR1, and no RSS |
| V78 | KE695978.1 (3286923-3287230) | CACAGCTGTACAGTCAGATGGGGAAGTGGGACAAAAACT |  |
| ΨV65 | KE695978.1 (3283977-3284171) | CACAGGGCTACAGACACAAGGGGAACTGAGTCAAAAACC | Deletion of FR1 and CDR1. |
| V79 | KE695978.1 (3282450-3282754) | CACAGTGATATACCCCAATGGGGAAGTGCAACAAAAGCT |  |
| V80 | KE695978.1 (3268340-3268646) | CACAGTGATACAGCCCGATGGGGAACTGAGATATTAACC |  |
| ΨV66 | KE695978.1 (3262957-3263241) | CCCAGTGATAGAGCCTGGTGGGGAACTGAATCATAACCT | Two in frame stop codons in CDR2, two in frame stop codons in FR3, and one in frame stop codon in the last six bases. |
| V81 | KE695978.1 (3260963-3261272) | CACAGTGGTAGAGTCGAATGGGGAACTGAGGCATAAACC |  |
| ΨV67 | KE695978.1 (3253050-3253302) | ACACTGGCAGCAGTAATGCATGGTGCACAGTCATAAACC | Two in frame stop codons in FR1 and FR3 respectively, no YYC in FR3, and unusual heptamer of RSS. |
| V82 | KE695978.1 (3249790-3250117) | CACAGTAGAAGAGCTGCATGAAGTAGAGGAACAAAAACT |  |
| V83 | KE695978.1 (3240213-3240517) | CACAATGGTACGCTTAGATGGGGAACTGAGAAAAAAGCA |  |
| V84 | KE695978.1 (3217170-3217486) | CACTGTACTACAGCTGTATGGGGAACTGTAACCAAAACT |  |
| V85 | KE695978.1 (3214431-3214747) | CCCAGTGGTTTGATCAGAGTAGGAACTGAAGCAAAAACC |  |
| V86 | KE695978.1 (3212220-3212535) | CACAGTGCTGCAGGCCTGCAGGGAAGTGGGACAAGAACC |  |
| ΨV68 | KE695978.1 (3209800-3209974) | CTCAGTGTCATGTGCCATCTACTATCTGTAGAAATGTGG | Deletion of FR1 and CDR1, four in frame stop codons in FR3, and unusual nonamer of RSS. |
| ΨV69 | KE695978.1 (3202501-3202811) |  | Absence of splicing signal and of RSS |
