# Supplementary material for: A Comprehensive Analysis of the Phylogeny, Genomic Organization and Expression of Immunoglobulin Light Chain Genes in Alligator sinensis, an Endangered Reptile Species
Source: PLoS One. 2016 Feb 22;11(2):e0147704. doi: 10.1371/journal.pone.0147704 (PMC4762898; doi:10.1371/journal.pone.0147704)
Supplement: S3 Appendix — (DOCX) [file pone.0147704.s003.docx]

**The alignment of the deduced amino acid sequence of 86 functional V_λ_ genes in the *Alligator sinensis***

**Gene Names**   **V_λ_ Sequences**

**Ig_λ_V family1** (13 members)

<------------FR1------------><-CDR1--><-------FR2 -------->CDR<----------------FR3----------------->

VL11 SLAQYVVTQPP-SVSVSPGETTRLTCSGNS--ISG-KYVQWYQQKPGTAPL-LIIY---ENSKRPSGIPDRFSG--AKSGDTATLTITGVQAQDEADYYC

VL12 ...........-.........A.......N--.GR-.S.H......S....-....---S..N...........--.N......................

VL14 ...........-.........A.......N--.GS-.S.H...........-....---S..............--.N......................

VL16 ...........-.........A.......N--.GS-.S.H...........-....---S..............--.N......................

VL17 ...........-.........A.......N--.GS-.S.H...........-....---N.DS...........--.N..................E...

VL18 LF..N......-A........A......T-----.-S..H...........-R...---D........S.....--S...S...................

VL20 LF..N......-A........A......P-----.-S..H...........-....---D........S.....--S.......................

VL21 ...........-.........AP.....TN--.GS-YN............R-....---N.DS...........--.N...M..............E...

VL22 LF..N......-A........A......T-----.-S..H...........-....---G..N.....S.....--S...S.................H.

VL24 ...........-.................N--.GS-.S.H...........-....---N..............--.N.....................R

VL25 LF..N......-A........A......P-----.-S..Y...........-....---G..N.....S.....--S...S...................

VL26 ...........-AA.......A......T.--FRS-Y..............-Q...---...............--........................

VL27 ...........-AA.......A......T.--.GT-YH.............-Q...---...............--.N..................H...

**Ig_λ_V family2** (4 members)

<------------FR1------------><-CDR1--><-------FR2 -------->CDR<----------------FR3----------------->

VL2 ...........-E........AQ...RGEK--FDK-Y..Y.....S..V..-....---K.KE...D.......--AS......................

VL4 ....H..V...-A........AQ.....EM--..K-N.A....H....V.K-....---K..E...D......A--.S..T.L.................

VL7 ...........-A........AH.....EK--.NK-..A.........V.R-....---K.KE...D...Q...--.S..T...................

VL8 ....H..V...-A........AQ.....EM--..K-..A....H....V.K-....---K..E...D......A--.S..T.L.................

**Ig_λ_V family3** (2 members)

<------------FR1------------><-CDR1--><-------FR2 -------->CDR<----------------FR3----------------->

VL46 ...........-.........AQ....GDN--.A.-KN.Y...H.VS.S.V-....---NSR............--AS...M..............V...

VL49 ...........-.........AQ......N--.AS-.N.Y...H.VS.S..-....---NSRS...........--VS.....V...I........V...

**Ig_λ_V family4** (1 member)

VL32 Y.....P....-I........AQ....GNN--.GG-HN.H........V.L-....---D..K.....A...F.--....................Y...

**Ig_λ_V family5** (8 members)

<------------FR1------------><-CDR1--><-------FR2 -------->CDR<----------------FR3----------------->

VL36 SLAQYVMTQPP-SVSISPGQTAKLTCTGRN--VG--NYVSWHQQQPGSAPV-LFIY---ENTKRPSSIPDRFSG--ARSGNTATLTITGVQAQDEADYYC

VL41 ...........-.....TA..A......RN--.GS-.H.H...H.......-R...---S..N.....S.-...--F...S...................

VL42 C..........-....PS...A......RN--.AS-HG.............-Q...---.........A.....--S..ES.......D...........

VL43 .........S.-.........A......RN--.GS-N........S.....-....---D..N.....A.Q...--S...S........A..........

VL44 .F.........-.........A......RN--.GR-YS.............-Q...---S..N...S...Q..A--SI......................

VL52 ..P......S.-.........A......RN--.GS-YS.S.D......V..-....---S..I.A...S.QL..--S......................Y

VL53 .........S.-.........A..I...RN--.DS-N........S.....-....---.........A.....--S...S........A..........

VL55 .........S.-L........A......RN--.GS-YS.S.D..E...V..-....---S..I.A...S..LFD--S.................K....Y

**Ig_λ_V family6** (4 members)

<------------FR1------------><-CDR1--><-------FR2 -------->CDR<----------------FR3----------------->

VL28 ....D..R...-.........A......ED--FDS-NE.S...........-....---A.DS.ADE..E....--........................

VL29 ....D..R...-.........A......ED--FDS-NA.S...........-....---A.DS.ADE..E....--........................

VL30 .........L.-.........AM...M.RN--.DS-NS...........N.-....---Y.D..AD....Q...--S...S...................

VL31 ....D..R...-.........A......ED--FDS-.I.S...........-....---I.DS.ADE..E....--.....M..................

**Ig_λ_V family7** (8 members)

<------------FR1------------><-CDR1--><-------FR2 -------->CDR<----------------FR3----------------->

VL34 C...T......-L...F....AT.......--..S-HGPD........V..-...D---RSNN.A...S.....--S.......................

VL35 ....S.T....-.........AT....A..--...-FGAS...........-...G---N.N......S.....--S..E...........T........

VL37 C...TAP....-.........AT.....S.--.GS-NGAN...........-T...---Y.NN.....S.....--S.......................

VL38 C...TAP..L.-.........AT.....S.--.GS-YGAG...........-P..K---Y.NT.....S.....--S.......................

VL39 C...TAP....-.........AT.....S.--.GS-YGAT...........-T...---N.NN.....S.....--S.......................

VL40 C...TAP....-.........AT.....S.--.GI-VGAS...........-...D---S.N......S.....--S..............V........

VL51 C...TAP....-.........AT.....S.--.GS-YGAA...........-T...---S.NN.....S.....--S.......................

VL54 C...TAP....-.........AT.....S.--.GS-YGAS...........-T...---STNN.....S.....--S.......................

**Ig_λ_V family8** (3 members)

<------------FR1------------><-CDR1--><-------FR2 -------->CDR<----------------FR3----------------->

VL33 AG.........-...A...G.VT...ARS.GS..D-S.NS.......R...-....---G..E......S....SID..A..V......A.PD.......

VL64 AG.........-...A...G.VT...ARS.GSF.D-S.NS.......R...-....---..........S....SIDE.A.........A.PD.......

VL67 AG.........-...A...G.VT...ARS.GS..D-S.NS.......R...-....---...E......S....SID..A.........A.PD.......

**Ig_λ_V family9** (17 members)

<------------FR1------------><-CDR1--><-------FR2 -------->CDR<----------------FR3----------------->

VL48 VWL.P....E.LAS.......VT....LS.GA..SSNHPS........P.R-....---S.NS......T....--SI..QK..................

VL56 VWS.P....E.SAS.....G.VT....LS.GA..TSN.PA..R.......Q-Q...---YT.N...R..T..N.--SI..QK.I...M............

VL61 VWS.P....E.-A......G.VT....LS.GA..SSN.PG.L......P.R-....---NTNS...E..A....--SI..QK..........D.D.....

VL62 VWS.P....E.AVS-....G.VT....LS.GA..SNN.RG...K......Q-...S---N.NT......T....--SI..QK.V.....A.L..D.....

VL63 VRS.S....E.-.......G.VT....LS.GA..TSN.PG........P.R-Q...---STNS......T....--SI..QK.V...........S....

VL65 VWS.P....E.-A.....EG.VT....LS.GA..SSN.PG.L......P.R-Q...---RTNN......T....--SI..QK.A................

VL66 VWS.P....E.AVS.....G.VT....LSPGA..TGY.PA........P.Q-....---YA.S......T....--SI..QK.A.......V..D.....

VL68 VWS.P....D.-V.....E..VT....LS.GA..SSN.PG.L......P.R-....---DTMT......T....--SI..QK.A....R.....D.....

VL69 VWS.P....E.-A...A..G.VT....LS.GA..TNN.PG...K....P.R-Q...---STNS......T....--SIP.QN.A....EA..........

VL72 VWS.P....E.-A......G.VT....LS.GA..SSN.PG........P.R-....---NTNS...E..T....--SI..QK..................

VL73 VWS.P....E.-A......G.VT....LS.GA..SRN.PA........P.R-Q...---LT.S......T....--DI..QNN....MA...........

VL74 VRS.P....E.SVS.....G.VT....LS.GA..TGN.PS..........Q-..T.---.SN.......T....--SI..QK.A.....A..........

VL75 VWS.P....E.-V......G.VT....LS.GA..TSN.PA.L......P.R-Q...---ST.S......T....--SI..QK.V................

VL76 VWS.P....E.SVS.....G.VT....LS.GA..TGN.PS..........Q-....---.SN.......T....--SI..QK.A.....A..........

VL77 VWPRP....E.AVS.....G.VT....LS.GA..SSN.PG.L........R-Q...---.I........T....--SI..QK.......A.P..D.....

VL78 .SS.Q.Q.LK.-.EQ....G.VT.A..LS.GA.GDGN.P..V.....HV.R-....---ST.TQ.....A....--S...S.M......A.P..D.....

VL80 VWS.P....E.AVS-....G.VT....LS.GA..SSN.PG.L........R-....---.I........T....--SI..QK.A...A............

**Ig_λ_V family10** (1 member)

<------------FR1------------><-CDR1--><-------FR2 -------->CDR<----------------FR3----------------->

VL85 --CYAQ.....-.A.....G.V.....LGGSYTVSSNR.L.L.....N..RF..Y.FTES.KGMG....S....SRSG.DKEGY.....AVE..D.....

**Ig_λ_V family11** (1 member)

<------------FR1------------><-CDR1--><-------FR2 -------->CDR<----------------FR3----------------->

VL86 .G..K....QA-L..G.L...........G.YSI...G.G....TS.NP..S..Y.Y.V.R.G.G......L...A.G.--VGY..........D.....

**Ig_λ_V family12** (3 members)

<------------FR1------------><-CDR1--><-------FR2 -------->CDR<----------------FR3----------------->

VL70 .SS.Q.QPLT.-.EQ....G.VT.A..LS.GA.ADNS..H.L.....Q..R-....---ST.T......A....--S....AM......A.P..D.....

VL71 .SS.Q.Q.LK.-.EQ....G.VT....LS.GA.GDGN.P..V.....Q..Q-....---ST.T......A....--S...S.M......A.P..D.....

VL79 VWS.P....E.-A....LR.IVT....LS.GA..STN.PH..........R-Q...---NTDS......T....--SI..QK.V...K..........H.

**Ig_λ_V family13** (1 member)

<------------FR1------------><-CDR1--><-------FR2 -------->CDR<----------------FR3----------------->

VL83 .STKAK....S-..L....G.AS....TS--EDITGYT.S.L...FQNP.KY..Y.KDEAKQG.V....T...ASKDT.SS.CY.....AL...D.V...

**Ig_λ_V family14** (3 members)

<------------FR1------------><-CDR1--><-------FR2 -------->CDR<----------------FR3----------------->

VL57 .HS.PT...A.-.E...L.N.I.....LS.--QHSNYV.S....Q..QTLR-F.W.---SSGT.GD..S....VS--S..AIRY....NAR.D...T...

VL58 ..S.PT...Q.-.K...L.N.VS....LS.--EHSNY..H.....Q.Q..Q-F.WH---SEGI.GN.......VS--N.SAIRY....N.PG....I...

VL60 ..S.PT.....-.E...L.N.VS....LS.--QYSNY..A.....SSQ..R-F.W.---SKGT.GD.......VS--N.SAIR.....N...D...T...

**Gene Names V_λ_ Sequences**

**Ig_λ_V family15** (3 members)

<------------FR1------------><-CDR1--><-------FR2 -------->CDR<----------------FR3----------------->

VL47 ..S..T....S-.E...LRN.V.....IS.--GYTHGA.Y.L...G.NS.RY..W.NTDSSKHQG....S....SKDT.NKIGY....S...........

VL50 ..S..T.....-.E...L.N.V.....IS.--GKTLGG.A.....N.NS.RY....NTDS.KHQG....S....SKDT.NKIGY....NA..........

VL59 .FS.IT....S-.E...L.N.I.....LS.--GGTFGA.Y.L...G.NS.RY..W.NTDSSKHQG....S....SKDT.NKIGY....S...........

**Ig_λ_V family16** (1 member)

<------------FR1------------><-CDR1--><-------FR2 -------->CDR<----------------FR3----------------->

VL82 PSF.Q...VQS-.A...L.G.VT....QS.GA..TVSHAH.I...L.H..R-R...---GA.D.GP...E...A--SI.AS.M....A.A.....GA...

**Ig_λ_V family17** (11 members)

<------------FR1------------><-CDR1--><-------FR2 -------->CDR<----------------FR3----------------->

VL1 ANS.PA.....-AE.....N.V.....MS.GTSISDYA.Y........PTRY..Y.KSDS.KHQG....A....SKDT.S..GY...A..LT........

VL3 ARS.S......-.E...L.N.V.....VS.GTSISDYN.Y........P.RY..R.KSNS.KHQG....A....SKDT.S..GY...A..LT........

VL5 ARS.S......-.E...S.N.V....AMT.GTSISGYS.Y........P.RY..Y.KSDS.KHQS....A....SKDT.S..GY...AR.L.........

VL6 ARS.S......-AE.....N.V....AMS.GNSISVY..N........P.RQ..Y.KSDS.KYQG....A...ASKDT.S..GY...A..L.........

VL9 ARS.S......-AE.....N.V....AVT.GTSISGYS.L........T.RY..Y.KSDS.KHQG....A....SKDT.S..GY...A..L.........

VL10 ARS.S......-.E.....N.V...Y.MS.EDSISGS.TH........P.RY..T.KSESQKFPS....A....SKGT.R.A.Y...A.AL.....V...

VL13 .SS.S......-AE.....N.V....AMS.GTSISGYN.Y........P.RY..Y.KSDSEKHQG....A....SKDT.S..GY...A.AL.........

VL15 .SS.S......-AE.....N.V.....MS.GTSISGY..N........P.RY..Y.KSDS.KHQG....A....SKDT.S..CY...A.AL.........

VL19 .SS.S......-AE.....N.V.....MS.GTSISGYR.Y........P.RQ..Y.KSDS.KHQG....A....SKDT.S..GY...A..L.........

VL23 .SS.S......-AE...S.N.V....AMS.GTSISGY..Y........P.RH..R.KSDS.KHQG....A...ASKDT.S..CY...AAAL.........

VL45 ASS.PA.....-.E.....N.V.....MS.GTSISDYV.S...H.A..G.RY..A.KSDS.K.QGP...A...AFKDT.I..CY.....AP.........

**Ig_λ_V family18** (1 member)

<------------FR1------------><-CDR1--><-------FR2 -------->CDR<----------------FR3----------------->

VL84 .G..K....QA-L..G.L...M.....VSGGYSISSYG.G....TS.NP.RS..Y.YSVSR.G.G......L..SASG.--VGY..........D.....

**Ig_λ_V family19** (1 member)

<------------FR1------------><-CDR1--><-------FR2 -------->CDR<----------------FR3----------------->

VL81 .TY..T....S-.A..A....V..A.VTS.GSSITD.V.R.......EK.RY..Y.RDESTNH.G........ASKST.T..CF...AQ.......E...
