# Supplementary material for: A Comprehensive Analysis of the Phylogeny, Genomic Organization and Expression of Immunoglobulin Light Chain Genes in Alligator sinensis, an Endangered Reptile Species
Source: PLoS One. 2016 Feb 22;11(2):e0147704. doi: 10.1371/journal.pone.0147704 (PMC4762898; doi:10.1371/journal.pone.0147704)
Supplement: S4 Appendix — (DOCX) [file pone.0147704.s004.docx]

**Multiple sequence alignment of *Alligator sinensis* V_κ_ genes**

* 20 * 40 * 60 * 80 * 100 * 120 * 140 * 160

VK1 : TCCTCCGGGCAGATTGTGCTGACTCAGACCCCAGAGTCCCTGGCGGTGTCTCCAGGAGATCAAGTGACCATCAAGTGCAAAGCCAGC---TCCAGCCTTACTTATAGTAGCTCTTGGGGTAGC------------TATCAGCAATTAGCCTGGTACCAGCAG

VK2 : TCAAGTGGAGACCGTGTGATGACCCAGGCTCCAGAGTCCCTGGCCATGTCCCCTAGAGACAGAGTCACCATCAAGTGCAAATGCAGC---TCCAGCCTTACTTAC------TCGGGCA------------------GAGAAGAATTAGCCTGGTACCAGCAA

VK3 : TCCTCCGGGCAGATTGTGCTGACTCAGACCCCAGAGTCCCTGGCGGTCTCTCCAGGAGATCAAGTGACCGTCAAGTGCAAAGCCAGC---TCCAGCCTTACTTATACCTTCTCTGATAGCAACACCCCTAAATATTATCATCACTTAGCCTGGTACCAGCAG

VK4 : TCAAGTGGAGACCGTGTGATGACCCAGACTCCAGAGTCCCTGGCACCATCCCTGGGAGACACAGTCACCATCAAATGCAAATGCAGC---TCCAGCCTCACATCT------GCAGGAA------------------AGGAGTTCCTAGGCTGGTACCAGCAG

VK5 : TCCTCCGGGCAGATTGTGCTGACTCAGACCCCAGAGTCCCTGGCGGTGTCTCCAGGAGATCGAGTGACCATCAAGTGCAAAGCCAGC---TCCAGCCTTACTTATAGTAGCTCTGGGAGTACC------------TATCAGCTCTTACACTGGTACCAACAG

VK6 : TCAAGTGGGGCCATTGTGATGACCCAGACTCCACAATCCTTGGCTCTGTCTCCTGGAGACACGGCCACCATCAAGTGCAAATCCAGC---TCCAGTCTTACTTAC------TCAGGCA------------------GAGAAGAATTAGCCTGGTACCAACAA

VK7 : TCCTCCGGGCAGATTGTGCTGACTCAGACCCCAGAGTCCCTGACGGTGTCTCCAGGAGATCGAGTGACCATGAAGTGCAAAGCCAGC---TCCAGCCTTACTACTAGGATCATTAGTAGTAGC------------TATGAGCAATTAGCCTGGTACCAGCAG

VK8 : TCAAGTGGGGCCATTGTGATGACCCAGACTCCGCAATCCTTGGCTCTGTCTCCTCGAGACACGGCCACCATCAAGTGCAAATCCAGC---TCCAGTCTTACTTAC------TCAGGCA------------------GAGAAGAATTAGCCTGGTACCAACAG

VK9 : TCCTCCGGGCAGATTGTGCTGACTCAGACCCCAGAGTCCCTGGCGGTGTCTCCAGGAGATCGAGTGACCATCAAGTGCAAAGCCAGC---TCCAGCCTTACTTATAGTAGCTCTGGGAGAACC------------TATCAGGGCTTAGCCTGGTACCAACAG

VK10: TCAAGTGGAGACCGTGTGATGACCCAGACTCCAGAGTCCCTGGCCGTGTTCCCTGGAGACACCGCCACCATCAAGTGCAAATGCAGC---TCCAGCCTCACATCC------GCAGGAA------------------AGGAGTTCCTAGCCTGGTACCAGCAG

VK11: TCCTCGGGGCAGATTGTGCTGACTCAGACCCCAGAGTGCCTGGCGGTGTCTCCAGGAGATCGAGTGACCATCAAGTGCAAAGCCAGC---TCCAGCCTTACTCATATTAGATCTGAAATTAGC------------TATCAGGAATTAGCCTGGTACCAGCAG

VK12: TCCTCCGGGCAGATTGTGCTGAGACAGACCCCAGAGTCCCTGGCGGTGTCTCCAGGAGATCGAGTGACCGTCAAGTGCAAAGCCAGC---TCCAGCCTTACTTA------CTCAGGAA------------------ATGAATACTTAGCCTGGTACCAGCAG

VK13: TCCTCCGGGCAGGTTGTGGTGACTCAGACCCCAGAGTCCCTGGCGGTGTCTCCAGGAGATCAAGTGACCATCAAGTGCAAAGCCAGC---TCCAGCTTTACTTATAGTAGCT------------------------ATCAGGTCTTAGCCTGGTACCAGCAG

VK14: TCCTCCGGGCAGATTGTGCTGACTCAGACCCCAGAGTCCCTGGCGGTGTCTCCAGGAGATCGAGTGACCATCAAGTGCAAAGCCAGC---TCCAGCCTTACTTATAGTAGCTCTGGGAGTACC------------TATCAGCTCTTACACTGGTACCAGCAG

VK15: TCCTCCGGGCAGATTGTGGTGACTCAGACCCCAGAGTCCCTGGCGGTGTCTCCAGGAGATCGAGTGACCATCAAGTGCAAAGCCAGT---TCCAGCCTTGCTTCTGG------------TAGT------------TATCAGCTCTTAGCCTGGTACCAGCAG

VK16: TCCTCCGGGCAGATTGTGCTGACTCAGACCCCAGAGTCCCTGGCGGTGTCTCCAGGAGATCGAGTGACCATCAAGTGCAAAGCCAGC---TCCAGCCTTACTGATAGTCGCT------------------------ATCAATACTTAGCCTGGTACCAGCAG

VK17: TCCTCCGGGCAGATTGTGCTGACTCAGACCCCAGAGTCCCTGGCGGTGTCTCCAGGAGATCGAGTGACCATCAAGTGCAAAGCCAGC---TCCAGCCTTACTTATAGTAGCTCTGGGAGTACC------------TATCAGCTGTTAGCCTGGTACCAACAG

VK18: TCCAGTGGACAAGCTGTGCTGACTCAGACTCCAGCATCCCTGTCCCTGTTTCCAGGAGACAGGGTCACCATCAACTGCAGAGCCAGT---TCCAGCATTAAAGCCA------CATCAGAAGTTAG---------------CTACTTAAACTGGTACCAACAG

VK19: TCCAGCGGAGACATCCTCATCACTCAGACTCCAAAGTCCGTGGCAGTGTCTCCAGGGGACACAGTCACCATTAAGTGCAAAGCCAGT---TCCAGTGTTAG-----------CAGCAG----------------------CAATATGCACTTCTACCAGCAG

VK20: TCCAGTGGGGATGTCGTTGTGACTCAGACCCCCGCATCCCTGTCGGTGTCTCCAGGAGACACAGCCACCATCAACTGCAACACCGGTTCATACAGTACTAG-----------TAGCA----------------------------TAGCCTGGTACCAACAG

VK21: TCCAGCGGGGACATTGTGATGAACCAGACTCCAGAGTCCCTGGCAGTGTCGCCAGGAGACACCGCCACCATCACCTGCAAAGCCGTTACATACAGTGGTAG-----------CAACG----------------------------TAGCCTGGTTCCAACAG

VK22: TCCAATGGGGACGTTGTGCTGACTCAGACTCCAGAGTCCCTGACGGTGTCTCCTCAAGACACAGTCACCATCACCTGCAATGCCGGTTCATACACTAGTAG-----------TAGCA----------------------------TAGCCTGGTACCAACAG

VK23: TCCAGCGGGAACATTGTGATGAACCAGACTCCAGAGTCTCTGGCAGTGTCGCCAGGAGATACTGCCACCATCACCTGCAAAGCCGTTACATACAGTGGTAG-----------CAACA----------------------------TAGCCTGGTACCAACAG

VK24: TCCAATGGGGACGTTGTGCTGATTCAGACTCCAGAGTCCCTGACGGTGGCTCCTCAAGACACAGTCACCATCACCTGCAATGCCGGTTCATACACTAGTAG-----------TAGCA----------------------------TAGCCTGGTACCAACAG

VK25: TCCAGCGGCGACATTGTTGTGACTCAGACTCCAGAGTCCCTGGCTGTGTCCCCAGGAGACACAGTCACCATCAAGTGCAAAACCAGC---TCCAGTGTTAG-----------CAGCAG----------------------CA---TGGCCTTCTACCAGCAG

VK26: TCCAGTGGGGACATACTGCTGATGCAGATGCCAGAGTCCCTGGCAGTGTCTCCAGGAGAAACGATCACCATCATCTGCAAAGCCATTACATCTAGTGGCAG-----------CTACA----------------------------TACACTGGTACCAACAG

VK27: TCCAGCGGGGACATTGTTGTGACTCAGACTCCAGAGTCCCTGGCAGTGTCCCCAGGAGACACCGTCACCATCAAGTGCAAAACCAGC---TCTAGTGTTAG-----------CAGCTA----------------------CA---TGGCCTTGTACCAGCAG

VK28: TCCAGTGGTGACAATCTTGTGACTCAGACTCCAGAGTCCCTAGCTGTGTCTCCAGGAGACACAGTCACCATCAAGTGCAAAGCCAGC---TCCAGTGTTAT-----------TAGCAA----------------------CA---TGGGTTTGTACCAGCAA

VK29: TCCAGTGTGGACATCCTCATCAATCAGACTCCAGAGTCCCTGGTTGTGTCCCCAGGAGAAACAGTCACCATCAAGTGCAAAACCAGC---TCCAGTGGCAG-----------TGGCAG----------------------C---ATGCACTTGTACCAGCAG

VK30: TCCAGCGGGGACATTCTCGTGACTCAGACTCCAGAGTCCCTGGCCGTGTCCCCAGGGGACACAGTCACCATCAAGTACAAAACCATT---TCCAACATATT-----------TAGCCG----------------------CA---TGGCCTTGTACCAGCAG

VK31: GTCTGTGGAGACATTGCTCTGACTCAGACTCCAGAGTCCCTGGCAGTGTCTGCAGGGGACACCGTCACCATCAAGTGCAAAGCCAGCGAATACAGTGGTAG-----------CAGCAG----------------------CA---TGGCCTGGTACCAACAG

VK32: TCCAGTGGGGACTTCCTCATCACTCAGACCCCAGAGTCCCTGGCCGTGTCCCCAGGAGGCACAGTCACCATCAAGTGCAAAGCCAGT---TCCAGTGTTAG-----------CAGCTA----------------------C---ATGCACTTGTACCAGATG

VK33: TCCAGCGGAGACATTGTGATGACTCAGACTCCAGCATCCCTGGCAGTGTCTCCAGGAGACACCGTCACCATCAGCTGCAAAGCCAGC---TCCAGCACAGG-----------CAATGC---------------------TC----TCTCCTGGTACCAACAG

VK34: TCCAGCGGGGACATTGCTGTGACTCAGACTCCAGAGTCCCTGGCAGTGACTCCCGGGGGGACAGTCACCATCAAGTGCAAAGCCAGT---TCCAGTGTTGG-----------CAGCAA----------------------CA---TGGCCTTGTACCAGCAG

VK35: TCCCTGGGTGAAGTTGTTCTGACTCAGACTCCAGAAACCCTGGCCGCGTCCCCTGGAGACACAGTCACCATCAAGTGCAAGGCCAGT---ACCAGCATTAGCAAC------------------------------------TACATAGGCTGGTACCTCCAG

VK36: TCCAGCGGGGACATCGTCATCACTCAGACTCCAGAGTCCCTGGCAGTCTCTCCAGGGGACACAGTCACCATCAAGTGCAAAACCAGT---TCCAGCGTTAG-----------CAGCAA----------------------C---ATGAACTTGCACCAGCAG

VK37: TCCAGCGGGGACATTGTTGTGACTCAGACTCCAGAGTCCCTGGCAGTGTCCCCTGGAGAGACAGTCACCATCAAGTGCAAAACCAGC---TCCGGTGTTGG-----------CAACAA----------------------CA---TGCACTTCTACCAGCAG

VK38: TCCAGCGGGGACATCGTCATCACTCAGACTCCAGAGTCCCTGGCAGTGTCTCCAGGGGACACAGTCACCATCAAGTGCAAAACCAGC---TCCAGCGTTAG-----------CAGCAA----------------------C---ATGGCCTTGTACCAGCAG

VK39: TCCAGCGGGGACAGCCTTGTGACTCAGACTCCAGAGTCCCTGGCTGTGTCTCCAGGAGACACCGTCACCATCCAGTGCAAAACCAGT---TACAGTACAAG-----------AAACAA----------------------CA---TGGCCTTGTACCAGCAG

VK40: TCCAGCGGAGACATCCTTGTGACCCAGACTCCAGAAGCTGTGGTTGTGTCCCCGGGAGATACAGTCACCATCAAGTGCAAAACCAGC---TCCAGTGTTAG-----------CAACTG----------------------GA---TGAACTTCTACCAGCAG

VK41: TCCAGCAGGGACATTGTTGTGACTCAGACTCCAGAGTCCCTGGCCGTGTCCCCAGGAGACACAGTCACCATCAAGTGCAAAACCAGC---TCCAGTGTTAG-----------CAGCTA----------------------CA---TGGCCTTGTACCAGCAG

VK42: TCCAGCGGAGACATCCTTGTGACCCAGACTCCAGAGTCCCTGGCAGTGTCCCCGGGAGACACAGTCACCATCAAGTGCAAAACCAGC---TCCAGTGTTAG-----------CAAGTG----------------------GA---TGAACTTCTACCAGAAG

VK43: TCCAGCGGAGACATCCTCGTGACCCAGACTCCAGAATCTGTGGCAGTGTCCCCCGGAGACACAGTCACCATCAAGTGCACAGCCAGC---TCCAGTGTTAA-----------CAACTA----------------------CA---TGAGCTTTTACCAGCAG

VK44: TCAAGCGGGCAGGTCGTGATGACTCAGACTCCAGAGTCCCTGTCTGTGGTCCCGGGACAGACAGTCACTATCAACTGCAAGGCCAGT---TCCAGCATTGG-----------CTCC-------------------------TACGCACACTGGTACCAGAAG

VK45: TCCAGCGGGGACATTGTTGTGACTCAGACTCCAGAGTCCCTGGCCGTGTCCACAGGAGAGACAGTCACCATCAAGTGCAAAACCAGC---TCCAGTGTTAG-----------CAGCAG----------------------CA---TGGCCTTCTACCAGCAG

VK46: TCCTCCGGGCAGATTGTGCTGACTCAGACCCCAGAGTCCCTGGCGGTGTCTCCAGGAGATCAAGTGACCATCAAGTGCAAAGCCAGC---TCCAGCCTTACTTATAGTAGCTCTTGGGGTAGC------------TATCAGCAATTAGCCTGGTACCAGCAG

VK47: TCCTCTGGGCAAGTCGTGCTAACTCAGCCTCCAGAGTCCCTGTCGGACCCTGTGGGAGGAAGAGTGTCTGTCACCTGCAAAGCCAGT---GCCCGTATTAGCAAT------------------------------------TGCCTGAACCGGTACCACCAG

VK48: TCGACCGGGGACATTGTTGTGACTCAGACCCCAGAGTCCCTGGCAGTGTCCCCAGGAGACACAGTCACCATCCAGTGCAAAACCAGC---TCCAGTATCAG-----------CAGCAG----------------------CAGAATGAACTTGCACCAGCAG

VK49: TCCAGTGGAGACATCCTCATCACTCAAACTCCAGAGTCCCTGGCAGTGTCTCCAGGGGACACAGTCAGCATCAAGTGCAAAACCAGC---TCCAGTGTTAG-----------CAGCAG----------------------CAGGATGCACTTCTACCAGCAG

VK50: TCCAGTGGAGACATCCTCATCACTCAGACTCCAGAGTCCCTGGCAGTGTCTCCAGGGGACACAGTCACCATCAAGTGCAAACCCAGC---TCCAGTGTTAG-----------CAGCAG----------------------CTACATGCACTTCTACCAGCAG

VK51: TCCAGCGGGGACATCCTCATCACACAGACTCCAGAGTCCCTGGCCAAGTCCCCAGGAGACACAGTCACCATCAAGTGCACAGCCAGT---TCCAGTGTCAG-----------CAGCTG----------------------G---ATGCACTTGTACCAGCAG

VK52: TCCAGTGGGGACATTATCATCACTCAGTCTCCAGAGTCCCTGGCTGTGTCCCCAGGAGACACCGTCACCATCAAGTGCAAAACCAGC---TCCAGTGTCAG-----------CAGCAG----------------------C---ATGGACTTGTACCAGCAG

VK53: TACTGCGGGAACATCCTTGTGACTCAGACTCCCGAAGCCCTGATAGCATCTCCAGGAGACACAGTCACCATGAAGTGCAAAACCAGC---TCCAGTGCTGG-----------CAGTAG----------------------TA---TGCACTTTTACCAGCAG

VK54: TCCAGCGGGGACATTGTTGTGACTCAGACTCCAGAGTCCCTGGCCGTGTCCCCAGGAGACACAGTCACCATCAAGTGCAAAACCAGC---TCCAGTGTTAG-----------CAGCTG----------------------GA---TGGCCTTGTACCAGCAG

VK55: TCCAGTGGGGACAATCCTGTGACTCAGATGCCAGAATCTGTGGCAGTGGCTCTAGGAGACACTGTCACCATCAAGTGCAGAGTCAGC---TCCAGCATCAG-----------CAGCAA----------------------CA---TGGACCTATTCCACCAG

VK56: CTGAACGGAGACATTGTGGTAACTCAGACCCCTGCTTCCCTGGCAGTGTCCCCAGGGGAGACAGTCACCATTCGGTGCAAACCT------TCCAGCCCCGT-----------CGGCAA----------------------TGAAATGAACTTTTACCTGTTC

VK57: GCCCAGGGGCAGATTGTGGTGATGCAGTCACCAGAGTCTGTTGCTGTATCTGTGGGAGAGACCATCACTATTCAAAGCAGATCCAGC---AGGGGCATTGGAAA------------------------------------AGAAATGAGCTGGTACCAACAA

VK58: TCCTCTGGGCAAATCGTGCTAACTCAGTCTCCAGAGTCCCTGTCAGTCCCTGTGGGAGGAACAGTGTCTATCACCTGCAAAGCCAGT---ACCAGTGTTAGCACT------------------------------------TGCCTGAACTGGTACCAGCAG

VK59: TCCAGTGGGGGCATTTTAGTGACTCAGAGTCCAGAATCCCTAACAGTGTCTCCAGGAGACACAGTCACCATCAAGTGCAAAACCAGT---TCTGGCATTGG-----------CAGGGA----------------------CA---TGAACTTCTACCAGCAG

VK60: TCCAGTGGGGACATCGTTATAACACAGACTCCGGCTTCTGTGACGGCACTTCCAGGAGAGACGGTCACCATCCAGTGCAAGGCC------TCCAGCTCCAT-----------GAGTGA----------------------TGATATGGCCTTATTGCTGTTC

VK61: GCCCAGGGGCAGATTGTGGTGACTCAGTCTCCAGAATCTGTTGCTGTATCTGTGGGAGAGACTGTCACTATTCAATGCAGATCCAGC---AGAAGCATTGGCAC------------------------------------AGAAATGAGCTGGTACCAGCAG

VK62: TCCAGCGGAGACATCCTCATCACTCAGACTCCAGAGTCCCTGGCCGTGTCTCCAGGAGACACAGCCACCATCAAGTGCAAAGCCAGT---TCCAGCATCAG-----------CACCAG----------------------CA---TGCACTTTTATCAGCAG

* 180 * 200 * 220 * 240 * 260 * 280 * 300 * 320

VK1 : AAACCTGGACAAGCTCCAAAGCTCCTCATCTATGGTGGTTCCACACGGCAGTCAGGAGTCCCAGCCTGGTTCAGCGGCAGTGGCTCTGGCACTG---------ATTTCACTCTCACCATCAGTGGTGTTGCAGCTGAAGATGCTGGAGATTATTACTGTCA : 299

VK2 : AAACCTGGGCAAGCTCCTAGGCTCCTCATTACTTTTGCTTCCACACAACAACCGGGAGTCCCGACCTGGTTCAGCGGCAGTGGGTCTGGAATGG---------ATTTCACTCTCACCATCAGCAGTGTAGAAGCTGAAGACGCTGGTGATTATTATTGTCC : 287

VK3 : AAACCTGGGCAAGCTCCAAAGCTCCTCATCTATGGTGGTTCCACCCGGCAGTCGGGAGTCCCAGCCCGGTTCAGCGGCAGTGGCTCTGGCACTG---------ATTTCACTCTCACCATCAGTGGTGTTGCAGCTGAAGATGCTGGAGATTATTACTGTCA : 311

VK4 : AAACCTGGACAAGCTCCTAAACTCATCATCACTTTTGCTTCCACACGGCAACCGGGAGTCCCGGCCCGGTTCAGCGGCAGTGGGTCTGGAACAG---------ATTTCACTCTCACCATCAGCAGTGTACAAGCTGAAGATGCTGGAGATTATTACTGTCA : 287

VK5 : AAACCCGGGCAAGCTCCAAAGCTCCTCATCTATGATGCTTCCACACGGCAGTCAGGAGTCCCAGCCCGGTTCAGCGGCAGTGGCTCTGGCACTG---------ATTTCACTCTCACTATCAGCAGTGTTGAAGCTGAAGATGACGGAGATTATTATTGTCA : 299

VK6 : AAACCTGGGCAAGCTCCAAAGCTCCTCATCTATAACGCTGTCACCCGGCAGTCGGGAGTCCCAGCCCGGTTCAGCGGCAGTGGCTCTGGCACTG---------ATTTCACTCTCACCATCAGCCATATAGAAGCTGAAGATGCTGGAGATTATTACTGTCA : 287

VK7 : AAACCTGGACAAGCTCCAAAGCTCCTCATCTATCTTGGTTCCACACGGCAGTCGGGAGTGCCAGCCAGGTTCAGCGGCAGTGGCTCTGGCACCG---------ATTTCACTCTCACCATCAGCAGTGTTGAAGCTGAAGATGCTGGAGATTATTACTGTCA : 299

VK8 : AAACCTGGACAAGCTCCAGAGCTCCTCATCTATGATGCTGTCACCCGGCAGTCGGGAGTGCCAGCCCGGTTCAGCGGCAGTGGCTCTGGCACTG---------ATTTCACTCTCACCATCAGCCATATTGAAGCTGAAGATGCTGGAGATTATTATTGTCA : 287

VK9 : AAACCTGGGCAAGCGCCTAAGCTCCTCGTCTATGATGCTGTAACCCGGCAATCAGGAGTCCCAGCCCGGTTCAGCGGCAGTGGCTCTGGCACTG---------ATTTCACTCTCACCATCAGCAGTGTTGAAGCTGAAGATGCTGGAGATTATTATTGTCA : 299

VK10: AAACCTGGACAAGCTCCTAAACTCATCATCACTTTTGCTTCTACACGGCAACCGGGAGTCCCGGCCCGCTTCAGCGGCAGTGGGTCTGGAACAG---------ATTTCATTCTCACCATCAGCCGGGTGGAAGCTGAAGATGCTGGACATTATTACTGTCA : 287

VK11: AAACCTGGGCAAGCTCCAAAGCTCCTCATCTACCTTGGTTCCACCCGGCAGTCGGGAGTCCCAGCCCGGTTCAGTGGCAGTGGCTCTGGCACTG---------ATTTCACCCTCACCATCAGCAGTGTTGAAGCTGAAGATGCTGGAGATTATTATTGT-- : 297

VK12: AAACCTGGACAAGCTCCAAAGCTCCTCATCTATGGTGGTTCCACCCGGCAGTCGGGAGTGCCAGCCCGGTTCAGCGGCAGTGGCTCTGGCACTG---------ATTTCACTCTCACCATCAGCAGTGTTGAAGCTGAAGATGCTGGAGATTATTACTGTCA : 287

VK13: AAACCTGGACAAGCTCCAAAGCTCCTCATCTATCGTGCTTCCACACAGCAGTCAGGGCTGCCAGCCCGGTTCAGCGGCAGTGGCTCTGGCACTG---------ATTTCACTCTCACCATCAGCAGTGTTGAAGCTGAAGATGCCGGAGATTATTATTGT-- : 285

VK14: AAACCTGGACAAGCTCCAAAGCTCCTCATCTATGATGCTTCCACACGGCAGTCAGGAGTCCCAGCCCGGTTCAGCGGCAGTGGCTCTGGCACTG---------ATTTCACTCTCACCATCAGCAGTGTTGAAGCTGAAGATGCTGGAGATTATTATTGTCA : 299

VK15: AAACCTGGGCAAGCTCCAAAGCTCCTCATCTATTTTGGTTCCACACGGCAATCAGGAGTCCCAGCCCGGTTCAGCGGCAGTGGCTCTGGCACCG---------ATTTCACTCTCACCATCAGCAGTGTTGAAGCTGAAGATGCTGGAGATTATTACTGTCA : 287

VK16: AAACCTGGACAAGCTCCAAAGTGCCTTATCTATGATGCTGTCACCCGGCAGTCGGGAGTGCCAGCCCGGTTCAGCGGCAGTGGCTCTGGCACCG---------ATTTCACTCTCACCATCAGCAGTGTTGAAGCTGAAGATGCTGGAGATTATTACTGTCA : 287

VK17: AAACCTGGACAAGCTCCAAAGCTCCTTATCTATAACGCTGTCACCCGGCAGTCGGGAGTGCCAGCCCGGTTCAGCGGCAGTGGCTCTGGCACTG---------ATTTCACTCTCACCATCAGCAGTGTTGAAGCTGAAGATGCTGGAGATTATTACTGTCA : 299

VK18: AAACCAGGGCAGGCTCCTAAGCTCCTTATCCGCTATGCCTCCAGTCGTCCCTCTGGGATCCCAGATCGGTTCAGTGGCAGTGGGTCTGGCACCG---------ACTTCACTTTCACTATCAGCCGGGTTGAAGCTGGAGATGCTGCAAATTATTACTGTCA : 290

VK19: AAACCAGGGCAGGCTCCTAAGCTCCTGCTCTATAGCACAAGCAGCCGCCCGTCTGGGATCCCCGCCCGGTTTAGTGGCAGTGGGTCTGGCACCG---------ACTACACTTTCACCATCAGCCACATGGAAGCTGGTGATGCTGGAGATTATTACTGTCA : 278

VK20: AAACCAGGACAAGCTCCTAAGCTCCTTATCTATAGCAAAACCTCCCGCCCCTCCGGGATCCCCGACCGGTTCAGCGGCAGTGGCTCTTCCACTG---------CCTTCACTTTCACCATCAGCCGGGTTGAAGCAGGTGATGCTGGAGATTATTATTGTCA : 275

VK21: AAATCAGGACAACCTCCTAAGCTTCTTATCTACAGCACAAACTCGCGGCCCTCCGGGATCCCCGACCGCTTCAGTGGCAGTAGGTCAGGCAGTG---------ACTACACTCTCACCATCAGCCGGGTGGAAGTAGGCGATGCTGGCGACTATTACTGTC- : 274

VK22: AAATCAGGGAAATCTCCTAAGCTTCTCATCCACAGCACGAGCTCCCGTGCCTCTGGGATCTCAGACCGCTTCAGCGGTGCTAGGTCAGGCAGTG---------ACTACACTCTCACCATCAGCCGGGTTGAAGCAGGTGATGCTGGAGATTATTACTGCCA : 275

VK23: AAATCAGGACAACCTCCTAAGCTTCTTATCTACAGCACAAACTCGCGGCCCTCCGGGATCCCCGACCGGTTCAGTGGCAGTAGGTCGGGCAGTG---------ACTACACTCTCACCATCAGCCGGGTGGAAGTAGGCGATGCTGGCGACTATTACTGTC- : 274

VK24: AAATCAGGGAAATCTCCTAAGCTTCTCATCTACAGCACGAGCTCCCGTGCCTCTGGGATTTCAGACTGTTTCAGCGGTGCTAGGTCGGGCAATG---------ACTACACTCTCAACATCAGCCGGGTTGAAGCAGGTGATGCTGGAGATTATTACTGTCA : 275

VK25: AAACCAGGGCAGGCTCCTAAGCTCCTTCTCTACGGCACGAACAGACGCGCCTCAGGGATCCCCGACCGGTTCAGTGGCAGTGGGTCTGGGACTG---------ACTTCACTTTCACCATCAGCCGGGTGGAACCTGGAGATGCTGCAGATTATTACTGT-- : 273

VK26: AAAAAAGGGCAACCTCCTACACTCCTTATCTCTGGCACAAACTCCCGTGTCTTGGGGATTCCAGACCAGTTCAGCAGCAGTAAATCAGACAGCA---------ACTACACTCTCACAATCAGCCGGGTTGAAGCAGATGATGCTGGAGATTACTATTGTC- : 274

VK27: AAACCAGGGCAGGCTCCTAAGCTCCTTCTCTACAGCACAAACAGCCGCCCCTCCGGGATCCCCGACCGGTTCAGTGGCAGTGGGTCTGGCACCG---------ACTTTACATTCACCATCAGCCGGGTGGAAGCTGGTGATGCTGGAGATTATTACTGTCA : 275

VK28: AGACCAGGGCAGGTTCCTAAGCTTCTTCTCTATGGCACAAACCACCGCCCCTCTGGGATCCCCGACCGGTTCATTGGCAGTGGGTCTGGCACTG---------ACTTCACTTTCACCATCAGCCAGGTTGAAACTGGTGATGCTGGAGCTTATTACTGTCA : 275

VK29: AAACCAGGGCAGGCTCCAAAGCTGCTGCTCTATGGCACAAACAGCCGTCCCTCCGGGGTCCCCGACCGGTTCAGTGGCAGTGGGTCTGGCACCG---------ACTTCACTTTCACCATCAGCCGGGTAGAAGCTGGGGATGCTGGAGATTATTACTGTCA : 275

VK30: AAACCAGGGCAGGCCCCTAAGCTCCTTCTGTATGGCACAAGCAGCCGCCCCTCCGGGATCTTCGACCGGTTCAGCGGCAGTGAGTCTGGCACCG---------ACTTCGCTTTCACCATCAGCCGGGTGGAGGCTGGTGATGCTGGAGACTATTGCTGTCA : 275

VK31: AAATCAGGACAAGCTCCAAAGCTCCTCATCTACGATGTTTCCTCTCGCCCCTCTGGGATCCCTGACCGGTTCAGTGGCAGTGGGTCTGGCACCG---------ACTTCACTCTCACCATCAGCCGGGTGGAAGCTGGTGATGCTGGAGATTATTACTGTCA : 278

VK32: AAACCAGGACAGGCTCCTAAGCTCCTTCTCTACTACACAAAGGTCTGCCCCTCCAAGATCCCTGACCTGTTCAGTGGCACCGTGTCTGGCAGTGATG------ACTACATTTTCACCATCAGCCGGGTTCAAGCTGATGATGCTGGAGATTATTACTGTCA : 278

VK33: AAACCAGGCCAAGCTCCTAAACTTCTTATATATAGCGGCACCAGTCTTGCCTCTGGTGTCCCGTCCCGGTTCAGTGGCCGTCAGACTGGGAGCGGCTACACTGACTACGCCCTCACGATCAGCGGGGTTCAGAGTGAAGATGCTGGAGATTATTACTGTCA : 284

VK34: AAACCAGGGCAGGCTCCTAAGCAACTTTTATATGGAACAAACAACCGCCCCTCCGGGATCCCCGACCGGTTCAGTGGCAGTGGGTCTGGCACCG---------ACTTCACTTTCACCATCAGCCGTGTGGAAGTGGATGATGCAGGAGATTATTACTGTCT : 275

VK35: AAACCAGGAGAAGCCCCAAAGCTCCTGATCTACGATTCTACCAACCGTGTCTCTGGGATCCCCGACAGGTTCAGCGGCAGTGGGTCTGGCACTG---------ATTTCACTCTCACCATCAGCAGGGTTGAAACCAAGGATGCTGGAGATTATTACTGTCA : 275

VK36: AAACCAGGGCAGGCTCCTAAGCTCCTTCTGTATGGCACAAGCAACCGCCCCTCGGGGATCCCCGACCGGGTCAGTGGCAGTGGGTCTGGCACCG---------ACTTCACTTTTACCATCAGCCGGGTGGAAGCTGGGGATGCTGGAGATTATTACTGTCA : 275

VK37: AAACCAGGGCAGGCTCCAAAGCTCCTTGTCTACTACGCAAGCAGGCGCCCCTCCGGGATCCCCGACCGGTTCAGTGGCAGTGGGTCTGGCACTG---------ACTTTACATTCACCATCAGACAGGTGGAAGCTGGAGATGCTGGAGATTATTACTGTCA : 275

VK38: AAACCAGGGCAGGCTCCTAAGTGCCTTCTATATGGCACAAACAGCCGCCCCTCGGGGATCCCTGACCGGTTCAGTGGCAGTGGGTCTGGCACTG---------ACTTCACTTTCACGATCAGCCGGGTGGAAGCTGGGGATGCTGGAGATTATTACTGTCA : 275

VK39: AAACCAGGGCAGGCTCTGAAGCTCCTTCTCTACAGCACAAACAACCGCCCCTCCGGGATCCCCGACCGGGTCAGTGGCAGTGGGTCTGGCACTG---------ACTTCACTTTCACCATCAGCCGGGTGGAAGCTGGGGATGCTGGAGACTATTACTGTCA : 275

VK40: AAACCAGGGCAGGCTCCAAAGCTCCTTGTCTACAAAACAAGCAACCGCCCCTCGGGGATCCCCGACCGGGTCAGTGGCAGTGGGTCTGGCACCG---------ACTACACTTTCACCATCAGCCGGGTGGAAGCTGGGGATGCTGGAGATTATTACTGTCA : 275

VK41: AAACCAGGGCAGGCTCCTAAGCTCCTTCTCTACTACACAAGCAATCGCCCCTCAGGGATCCCCGAGCTGTTCAGTGGCAGTGGGTCTGGCACTG---------ACTTCACTTTCACCATCAGCCGGGTTGAAGCTGGTGATGCTGGAGATTATTACTGCCA : 275

VK42: AAACCAGGGCAGGCTCCTAAAAACCTTCTCTACAAAACAAGCAACCGCCCCTCCGGGATCCCCGACCGGTTCAGTGGCAGTGGGTCTGGCACCG---------ACTACACTTTCACCATCAGCCGGGTGGAAGCTGGAGATGCTGGAGATTATTACTGTCA : 275

VK43: AATCCAGGGCAGGCTCCAAAGCTCCTTGTCTACTACACAAACAGGCGCCCCTCGGGGATCCCCGACTGGTTCAGTGGAAGTGAGTCTGGCACCG---------ACTACACTTTCACCATCAGCCAGGTGGAAGCTGGGGATGCTGGAGATTATTACTGTCA : 275

VK44: AAACCCGGGCAGGCACCCAAGCTTGTTATCTATAATGCCAACAGCCGCCCGTCTGGGATCCCCGACCGGTTCAGTGGCAGTGGGTCTGGCACTG---------ACTTCACGCTCACCATCAGCCGGGTGGAAGCCGATGATGCTGGAGACTATTATTGC-- : 273

VK45: AAACCAGGGCAGGCTCCTAAGCTCCTTCTCTACGGCACGAACAGACGCGCCTCAGGGATCCCCGACCGGTTCAGTGGCAGTGGGTCTGGGACCG---------ACAGCACTTTTACCATTGTCCGAGTGGAAGCTGATGATGCTGGAGATTATTACTGT-- : 273

VK46: AAACCTGGACAAGCTCCAAAGCTCCTCATCTATGGTGGTTCCACACGGCAGTCAGGAGTCCCAGCCTGGTTCAGCGGCAGTGGCTCTGGCACTG---------ATTTCACTCTCACCATCAGTGGTGTTGCAGCTGAAGATGCTGGAGATTATTACTGTCA : 299

VK47: AAACCTGGACAAACTCCCAAGCTCGTGGTCTACTATGCCAGCATCCTCCAGGCCGGGGTCCCTGCCCGGTTCATTGGCAGTGGTTCAGGCACGG---------ATGTCACCTTCACCGTCAGCAATGTTGAAGCCGACGATGCTGCAGATAACTACTGTCA : 275

VK48: AAACCAGGGCAGGCTCCTAAACTTCTTCTCTACGGCACAAACAACCGCCTCTCTGGGGTCCCCAACCGGTTCAGTGGCAGTGGGTCTGGAACTG---------ACTACACTTTTACAATCAGCCGGGTGGAAGCTGGTGATGCCGGAGACTATTACTGTGA : 278

VK49: AAACCAGGGCAGGCTCCTAAGCTCCTTGTCTACTACGCAAGCAGGCGCCCCTCCGGGATCCCCGACCGGTTCAGTGGCAGTGGGTCTGGCACCG---------ACTACACTTTCACCATCAGCCGGGTGGAAGCTGGAGATGCCGGAGATTATTACTGTCA : 278

VK50: AAACCAGGGCAGGCTCCTAAGCTCCTTCTCTACAGCACAAACAGTCGCCCCTCCGGGATCTCTGATCGGTTCAGTGGCAGTGGGTCTGGCACCG---------ACTACACTTTCACCATCAGCCGGGTGGAAGCTGGAGATGCTGGAGATTATTACTGTCA : 278

VK51: AAACCAGGGCAGGCTCCTAAGCTCCTTGTCTACAGAACAAATAACCGCCCCTCCGGGATCCCCGACCGGTTCAGTGGCAGTGGGTCTGGCACCG---------ACTTCACTTTCAGCATCAGCCGGGTGGAAGATGGTGATGCTGGGGATTATTACTGTGG : 275

VK52: AAACCAGGGCAGGCTCCTAAGCTCCTTGTCTACAACACCAACAATCGTGCTTCTGGGATCCCTGACCGGTTCAGTGGCAGTGGGTCTGGCACTG---------ACTTCACTTTCACCATGAGCCGGGTTCAAGCTGATGATGCAGGAGATTATTACTGTTT : 275

VK53: AAACCAGGGCAGGCTCCCAAGCTCCTTGTCTATGACACAAACAGCCGCCCCTCCGGGATCCCCGACCGGTTCAGTGGCAGTGGGTCTGGCACTG---------ACTTCACTTTCACCATCAGGCAGGTGGAAGGTGGTGATGCTGGAGACTATTACTGTCA : 275

VK54: AAACCAGGGCAGGCCCCTAAGCTCCTTCTCTACAGAGCAAGCAACCGCCCCTCAGGGATCCCCGACCGGTTCAGTGGCAGTGGGTCTGGGACTG---------ACTTCACTTTCACCATCAGCCGGGTTGAAGCTGGTGATGCTGGAGATTATTACTGCCA : 275

VK55: AAACCAGGGCAACCTCCTAGGATCCTTCTCTACAAAACAAACAGCCGCCCCTCCGGGATCCCCGACCGGTTCAGTGGCAGTGGGTCTGGCACCG---------ACTTCACCTTTACCATCAGCCGGGTGGAAGCTGAAGATGCTGGAGATTATTACTGTCA : 275

* 180 * 200 * 220 * 240 * 260 * 280 * 300 * 320

VK56: AGAGGAGGAGCAAGACCCCGGCTCATGATCCACCATGCAACCAGCCGCATCCCCGGAGTCCCCGAGAGGTTCAACGGCACACACAGCAGCACTG---------ATGTCACCTTCACCATTCGCGGGGTGCAGATGGAAGACGCAGGCAACTACTACTGCGG : 275

VK57: AACCCTGGACAAGCTCCCAAGCTTCTGGTCTACAGTGG---CAACCATTACACTGGCAACCCCGACCTGTTCAGCCCCAGTGGGTCTGGTACTG---------ACTTCACTCCCACGATAAGTGGGTTTGAAGCTGAGGATACTGGAGATTATTACTGTCA : 272

VK58: AAACCTGGACAAGTTCCCAACCTCATGATCTACTATGCCAGTAGCCTCCAGTCTGGGGTCCCTGCCTGGTTCAGTGGCAGTGGTTCAGGCACGG---------ATTTCACCTTCACCATCAGCAGTGTTGAAGCCGACGATGCTGCAGATTACTACTGTCA : 275

VK59: AAACCAGGACAACCTCCAATGCTTCTTCTCCATGACACAAACAAAGGCCCTTCTGGGATCCTAGCTCAGTTCAGTGGCAGTGGGTCTGGCACTG---------ACTTCACTTTTACCGTCAGCCGGGTGCAAGTTGGAGATTCTGGAGATTATTACTGTCA : 275

VK60: AAATCTGGACAAACTCCCAAGCTCCTCATTCATGATGGAAACAACCGTGTCACTGGGGTCCCCGACCGGTTCAGTGGCAGCTACAGCGGCACCG---------ACTTCACATTCACCATCCGCGGGGTGCAGGTGGAAGATGCTGGTGACTACTACTGTGG : 275

VK61: AAACCTGGACAGGCTCCCAAGCTCCTGATCAACAGCGT---CAGCCGTTACACTGGCATCCCTGACCGGTTCAGCCCCAGTGGGTCTGGCACTG---------ACTTCACTCTCACGATCAGTGGGGTTGCAGCTGAGGATGCTGGAGATTATTACTGTCA : 272

VK62: AAATCAGGGCAGGCTCCTAAGCTCCTTATCAAATATGCTTCCACTCGTATCTCTGGTGTCCCAGACCGGTTCAGCGGCAGTGGGTCTGGGACAG---------ATTTCACATTCACAATCAGTCAGGTTGAAGCAGAAGATGCTGGTGATTACTATTGCCA : 275
