# Supplementary material for: A Comprehensive Analysis of the Phylogeny, Genomic Organization and Expression of Immunoglobulin Light Chain Genes in Alligator sinensis, an Endangered Reptile Species
Source: PLoS One. 2016 Feb 22;11(2):e0147704. doi: 10.1371/journal.pone.0147704 (PMC4762898; doi:10.1371/journal.pone.0147704)
Supplement: S5 Appendix — (DOCX) [file pone.0147704.s005.docx]

**The *Alligator sinensis* V_κ_ gene DNA segment in contigs**

| \| **Name** \|  \|  \| \| --- \| --- \| --- \| | **V-region position in contig** | **RSS** | **Defects in V_κ_ pseudogenes and ORFs** |
| --- | --- | --- | --- | --- | --- | --- |
| ΨV1 | BAC Y329F14 (1616-1908) | CACACAGTTATATAACCCAATATAAAAA | One in frame stop codon in FR1; Two in frame stop codons in FR2, Two in frame stop codons in FR3. |
| ΨV2 | BAC Y146B4 (108761-109065) | CACAGTGCTAGACCAGCACAAAAACCTC | One in frame stop codon in FR2, four in frame stop codons in FR3. |
| ΨV3 | BAC Y146B4 (93653-93959) | CACAGTGCTACAACCCACAGTACAAAAA | One in frame stop codon in FR1, One in frame stop codon in FR2, Two in frame stop codons in FR3. |
| V1 | BAC Y146B4 (82620-82941) | CACAGTGATACAAACCCATACAAAAAGC |  |
| V2 | BAC Y146B4 (65443-65752) | CACGGCGATACAAACCAGTACAAAAACC |  |
| V3 | BAC Y146B4 (53133-53466) | CACAGTGATACAAACCCATACAAAAACC |  |
| ΨV4 | BAC Y146B4 (51181-51359) | CACAAGGATACAGACTGGCACAAAATCC | Deletion of FR1 and CDR1, and one in frame stop codon in FR3. |
| V4 | BAC Y146B4 (49832-50141) | CACAGTGATACAAACCAGTACAAAAACC |  |
| V5 | BAC Y146B4 (44399-44720) | CACAGTGATACAAACCCATACAAAAACC |  |
| ΨV5 | BAC Y146B4 (41063-41353) | AGCCTTCACTCAGACCACAACAAAAACC | One in frame stop codon in FR3. |
| V6 | BAC Y146B4 (32581-32890) | CACAGTGATACAAACCCATACAAAAACC |  |
| ΨV6 | BAC Y146B4 (27769-27946) | CACAGTGATACAACCCAGTACAAAAACC | Deletion of FR1 and CDR1, and three in frame stop codons in FR3. |
| ΨV7 | BAC Y146B4 (26865-27075) | CACAGTGACAGTGGCCAGGTCAGAAACC | One in frame stop codon in FR2. |
| V7 | BAC Y146B4 (23278-23599) | CACAGTGATACAAACCCATACAAAAACC |  |
| ΨV8 | BAC Y77E6 (109905-110195) | AGCCTTCACTCAGACCACAACAAAAACC | No leader peptide. |
| V8 | BAC Y77E6 (102927-103236) | CACAGTGATACAAACCCATACAAAAACC |  |
| ΨV9 | BAC Y77E6 (98001-98315) | CACAGTGATACAACCCAGTACAAAAACC | One in frame stop codon in the leading peptide |
| ΨV10 | BAC Y77E6 (95977-96279) |  | One in frame stop codon in FR2 and deletion of RSS，and seriously mutated FR3. |
| V9 | BAC Y77E6 (92440-92761) | CACAGTGATACAACCCCATACAAAAACC |  |
| ΨV11 | BAC Y77E6 (87046-87214) | AGCCTTCACACAGACCACAACAAAAACC | Deletion of FR1 and CDR1. |
| V10 | BAC Y77E6 (78564-78873) | CACAGTGATACAAACCAGTGCAAAAACC |  |
| V11 | BAC Y77E6 (73607-73930) | CACAGTGATACAACCCAGTACAAAAACC |  |
| V12 | BAC Y77E6 (65749-66070) | CACAATGATACAAACCCATACAAAAACC |  |
| ΨV12 | BAC Y77E6 (60278-60450) |  | Deletion of FR1, CDR1 and RSS，and one in frame stop codon in FR2 |
| V13 | BAC Y77E6 (56421-56730) | CACAGTGATACAAACCCATACAAAAACC |  |
| ΨV13 | BAC Y77E6 (49708-49997) | CTCTCTGATACAGACCCATACAAAAACC | One in frame stop codon in CDR2 and seriously mutated CDR2. |
| V14 | BAC Y77E6 (47086-47390) | CACAGTGATACAATCCCATACAAAAACC |  |
| ΨV14 | BAC Y77E6 (43873-44050) | AGTCTTCACACAGACCACAACAAAAACC | Deletion of FR1 and CDR1. |
| V15 | BAC Y77E6 (40120-40441) | CACAGTGATACAAACCCATACAAAAACC |  |
| ΨV15 | BAC Y77E6 (31925-32240) | CACAGTGATACAAACCCATACAAAAACC | Deletion of FR1, CDR1 and leader peptide. |
| V16 | BAC Y65C14 (96545-96854) | CACAGTGATACAAACCCATACAAAAACC |  |
| ΨV16 | BAC Y65C14 (84118-84290) | TTCAGTGACACAGCCCAGTTCAAAAACC | Deletion of FR1 and CDR1, and one in frame stop codon in FR2. |
| V17 | BAC Y65C14 (80275-80584) | CACAGTGATACAAACCCATACAAAAACC |  |
| V18 | BAC Y65C14 (74983-75304) | CACAGTGATACAAACCCATACAAAAACC |  |
| ΨV17 | BAC Y65C14 (65088-65382) | CACAGTGATTCAGACCCATACAAAAACC | One in frame stop codon in FR3 |
| ΨV18 | BAC Y65C14 (59541-59839) |  | Two in frame stop codons in FR3 and deletion of RSS. |
| V19 | BAC Y65C14 (52590-52902) | CACAGTGATGCAGACCCATACAAAAACC |  |
| ΨV19 | BAC Y65C14 (42863-43162) | CACAGTAATACAGACCAGTTCAAAAACC | No leader peptide. |
| V20 | BAC Y65C14 (30560-30860) | CACAGTGATACAGAGCAGTACAAAAACC |  |
| ΨV20 | BAC Y65C14 (28742-29008) |  | One in frame stop codon in FR2 and deletion of RSS. |
| V21 | BAC Y65C14 (25017-25314) | CACAGTGATACAGACTGGTACAAAAACC |  |
| V22 | BAC Y65C14 (21733-22032) | CACAGTGATACAGACCACGACAAATACC |  |
| V23 | BAC Y65C14 (13050-13347) | CACAGTGATACAGACCCATACAAAAACC |  |
| V24 | BAC Y65C14 (9801-10100) | CACAGTGATACAGGCCATGACAAATACC |  |
| V25 | BAC Y65C14 (5005-5302) | CACAGTGATACAGACTAGTAAAAAAACC |  |
| ΨV21 | BAC Y146M19 (103763-103947) | CACAGCAATGTAAACCAGTACAAAAACC | Deletion of FR1 and CDR1, and seriously mutated CDR2. |
| V26 | BAC Y146M19 (97251-97546) | CACAGTGCTACAGACCAGCACAAAAACC |  |
| V27 | BAC Y146M19 (92386-92685) | CACAGTGATGCAGACCAGAACAAAAACC |  |
| ΨV22 | BAC Y146M19 (90671-90935) | CACAGTGCTACAGCCCCGTACAAAAACC | One in frame stop codon in FR2. |
| V28 | BAC Y146M19 (83662-83959) | CACAGTGCTACAGCCCAGTACAAAAACC |  |
| V29 | BAC Y146M19 (81583-81880) | CACAGTGCTTTGGCCCAATACAAAAACC |  |
| ΨV23 | BAC Y146M19 (68894-69128) |  | Deletion of FR1 and RSS. |
| ΨV24 | BAC Y146M19 (68451-68739) | CACAGTGATGCAGACCAGCACAAAATCC | One in frame stop codon in FR2. |
| V30 | BAC Y146M19 (67261-67558) | CACAGTGCTACAGCCCAATACAAAACCT |  |
| V31 | BAC Y146M19 (55113-55410) | CACAGTGCTACAGCTCATTACAAAAACC |  |
| ΨV25 | BAC Y146M19 (42641-42926) | CACAGTGCTACAGCCCCGTACAAAAACC | One in frame stop codon in CDR3 |
| ΨV26 | BAC Y146M19 (34589-34883) | CACAGTGCTACAGCCCCATGCAAAACCC | No splicing signal in the leading peptide. |
| V32 | BAC Y146M19 (31591-31891) | CACGGTGCTACAGCCCCGTACAAAAACC |  |
| V33 | BAC Y146M19 (29505-29805) | CACAGTGCTACAGCCCTGTACAAAACCC |  |
| ΨV27 | BAC Y146M19 (27339-27590) |  | Two in frame stop codons in CDR2 and FR3 respectively, and deletion of RSS. |
| V34 | BAC Y146M19 (26054-26360) | CACGGTGATACAGCCCCATGCAAAAACC |  |
| V35 | BAC Y146M19 (22954-23251) | CACAGTGCTACAGCCCCGTACAAAAACC |  |
| ΨV28 | BAC Y146M19 (19671-19965) | CACAGTGCTACAGCCTAATACAGAAAAC | Absence of GC splicing signal in the leading peptide. |
| V36 | BAC Y146M19 (15846-16143) | CACAGTGCTACAGACCCGCACAAAAACC |  |
| ΨV29 | BAC Y146M19 (10988-11274) | CACAGTGCTACAGCCCAGTACAAACCCC | One in frame stop codon in CDR1 and three in frame stop codons in FR3. |
| V37 | BAC Y146M19 (7782-8079) | CACAGTGCTACAGCCCCGTACAAAAACC |  |
| ΨV30 | KE697554.1(74422-74695) | CACAGTGCTACAGCCCAATACAAAAACC | No leader peptide and a little shorter. |
| V38 | KE697554.1 (65231-65525) | CACAGTGCTACAGCCCCGTACAAAAACC |  |
| ΨV31 | KE697554.1 (62374-62673) | CCCAGTGCTACTGCCCCATACAAAAGCC | No leader peptide |
| ΨV32 | KE697554.1 (57378-57651) | CACAGTGCTACAGACCCACACAAAAACC | One in frame stop codon in CDR1 and two in frame stop codons in FR3. |
| ΨV33 | KE697554.1 (54618-54915) | CACAGTGATACACTGCAGTACAAAAACC | No leader peptide. |
| ΨV34 | KE697554.1 (52022-52192) |  | Deletion of FR1, CDR1 and RSS. |
| V39 | KE697554.1 (51028-51325) | CACAGTGCTACAGCCCCGTACAAAAACC |  |
| ΨV35 | KE697554.1 (45677-45960) | CACAGCGATACACTGCAGTACAGAAACC | Two in frame stop codons in CDR1 and one in frame stop codons in FR3. |
| V40 | KE697554.1 (42754-43051) | CACAGTGCTACAGCCCCGTACAAAAACC |  |
| .ΨV36 | KE697554.1 (38868-39156) |  | Absence of splicing signal and deletion of RSS. |
| V41 | KE697554.1 (29757-30051) | CACAGTGCTACAGCCCCGTACAAAAACC |  |
| ΨV37 | KE697554.1 (18994-19291) | CACGGTGCTACAGCCCTGTACAAAAACC | Absence of splicing signal |
| V42 | KE697554.1 (8831-9128) | CACAGTGCTACAGCCCAGTACAAAAAGC |  |
| V43 | AVPB01043985.1 (615-910) | CACAGTGCTTCAGCCCCGTACAAAAACC |  |
| V44 | KE698055.1 (16232-16526) | CACAATGCTACAGCCCTGTACAAAAACC |  |
| ΨV38 | KE698335.1 (5311-5608) | CACAGTGCTACAGCCCTGTACAAAAACC | A little longer leader peptide |
| ΨV39 | KE698081.1 (14397-14691) | CACAGTGCTACAGCCCAATACAAAAACC | One in frame stop codons in FR2. |
| V partial 2 | KE698081.1 (2847-3054) |  |  |
| ΨV40 | KE698149.1 (2641-2859) | CACAGTGCTACAGCCCCATACAAAAACC | Two in frame stop codons in FR3 and seriously mutated CDR2. |
| ΨV41 | KE698149.1 (13821-14087) | CACAGTGATACACTGCAGTACAAAAACC | One in frame stop codons in FR1. |
| ΨV42 | AVPB01053098.1 (10490-10787) | CACAGTGCTACAGCCCCGTACAAAAACC | Absence of splicing signal. |
| ΨV43 | AVPB01053098.1 (4426-4619) |  | Absence of splicing signal and RSS, and no YYC |
| V45 | AVPB01053098.1 (2261-2562) | CACAGTGGTACAGACCAGCACAAAAACC |  |
| ΨV44 | KE698098.1 (9617-9902) | CACAGTGATGCACTGCAGTACAAAAACC | One in frame stop codon in FR1. |
| ΨV45 | KE698098.1 (2754-3039) | CAGAGTGCTACAGCCCCAGACAAAAATC | One in frame stop codon in FR3. |
| V46 | AVPB01130521.1 (1913-2211) | CACAGTGCTACAGCCCAATACAAAAACC |  |
| V partial 3 | KE697644.1 (80724-80977) |  |  |
| V47 | KE697644.1 (35094-35391) | TACAGTGACACAGCCCAGCACAAACACC |  |
| ΨV46 | KE697644.1 (3728-3288) |  | No YYC and no ATG in FR3 and leader peptide respectively, and deletion of RSS. |
| V48 | KE698428.1 (5116-5616) | CACAGTGCTACATCCCCATACAAAAACC |  |
| V49 | KE698356.1 (6576-6876) | CACAGTGCTTCATCCCCGTACAAAAACC |  |
| V50 | KE698585.1 (707-1007) | CACAGTGCTACAGCCCCGTACAAAAACC |  |
| V51 | AVPB01143799.1 (680-977) | CACAGTGCTACAGCCCCATGCAAAAACC |  |
| ΨV47 | KE698008.1 (16271-16544) | CACAGTGCTACAGACCCGCACAAAAACC | Two in frame stop codons in FR1. |
| ΨV48 | KE698008.1 (9827-10116) | CACAGTGCTACAGCCCCGTACAAAAACC | Absence of splicing signal AG, and one in frame stop codons in FR3. |
| ΨV49 | KE698008.1 (6585-6777) |  | Only FR1, and no other regions. |
| ΨV50 | AVPB01013186.1 (1304-1586) | CACAGTGCTACAGCCCAATACAAAAACC | One in frame stop codon in FR2. |
| V52 | KE698096.1 (10501-10798) | CACAGTGCCACAGCCCCATACAAAAACC |  |
| V53 | KE698096.1 (4032-4329) | CACAGTGCTACAGCCCCGTACAAAAACC |  |
| ΨV51 | KE695928.1(4398044-4398328) | CACAGTGATACACTGCAGTACAAAAACC | No leader peptide. |
| V54 | KE695928.1(4392985-4393282) | CACAGTGCTACAGCCCAGTACAAAAAGC |  |
| V partial 4 | KE695928.1(4389202-4389480) |  | Because of a gap in the contig |
| ΨV52 | KE695928.1(4384224-4384486) | CAGAGTGCTACAGCCCCAGACAAAAATC | Two in frame stop codons in FR3. |
| ΨV53 | KE695928.1(4373606-4373887) |  | One in frame stop codon in FR3, absence of RSS and seriously mutated CDR2. |
| V55 | KE695928.1(4371573-4371870) | CACAGTGCTACAGCCCCGTACAAAAACC |  |
| V56 | KE695928.1(4364799-4365096) | CACAGTGTTACACTTCAGCACAAAAACC |  |
| V57 | KE695928.1(4344013-4344307) | CACAGTGATGCAGCCCACTACACAAACC |  |
| V58 | KE695928.1 (4339628-4339925) | CACAGTGACACAGCCCAGCACAAAAACT |  |
| V59 | KE695928.1 (4336437-4336770) | CACAGTGCTACAGCCCCGTGCAAAAACC |  |
| V60 | KE695928.1 (4332845-4333142) | CACAGTGATACACTGCAGTACAAAAACC |  |
| V61 | KE695928.1 (4328468-4328762) | CACAGTGATACAACCCAGTACAAAAACC |  |
| ΨV54 | KE695928.1 (4326145-4326423) | CACAGTGATAAAACTCAGCACAAAAACC | One in frame stop codon in CDR1. |
| V62 | KE695928.1 (4319511-4319808) | CACAGTGCTACAGCCCAATACAAAAACC |  |
| ΨV55 | KE695928.1 (4316913-4317145) | CACAGTGATACAGACCAGTACAAAAACC | No leader peptide. |
| ΨV56 | KE695928.1 (4309664-4309851) | CACACAATGACACAGTCCACTACAAAACC | Deletion of FR1, CDR1. |
