# Supplementary material for: A Comprehensive Analysis of the Phylogeny, Genomic Organization and Expression of Immunoglobulin Light Chain Genes in Alligator sinensis, an Endangered Reptile Species
Source: PLoS One. 2016 Feb 22;11(2):e0147704. doi: 10.1371/journal.pone.0147704 (PMC4762898; doi:10.1371/journal.pone.0147704)
Supplement: S6 Appendix — (DOCX) [file pone.0147704.s006.docx]

**The alignment of the deduced amino acid sequence of 62 functional Vκ genes in the Alligator sinensis**

**Gene Names**   **Vκ Sequences**

**Ig**_κ_**V family1** (25 members)

<------------FR1------------><------CDR1-----><------FR2 ------>CDR<-----------------FR3------------------>

VK20 SSGDVVVTQTPASLSVSPGDTATINCNTGSYSTS-----------S-IAWYQQKPGQAPKLLIYSKTSRPSGIPDRFSGSGSS---T-AFTFTISRVEAGDAGDYYC

VK26 .......M.M.........E....I...I..SG.-----------Y-.H.....K..P.T...SG.N..VL....Q..S.K.D---.-...L.......D.......

VK28 ....N......E..A......V..K.KAS.SVI.-----------N-.GL.......V......GTNH.........I....G---.-D......Q..T....A...

VK29 ..V....N...E..V....E.V..K.K.S.SG.G-----------.-.HL..............GTN...............G---.-D..................

VK30 ...........E..A......V..KYK.I.NIF.-----------R-..L..............GT.......F......E.G---.-D.A..............C.

VK31 VC...A.....E..A..A...V..K.KASE..G.-----------.S.......S.........DV................G---.-D..L...............

VK33 ..............A......V..S.KAS.S.G------------NA.S................G..LA....S....RQ.GSGY.-D.AL...G..SE.......

VK35 .L.E.......E..AA.....V..K.KAS.SI.------------NY.G..L............DS.N.V............G---.-D..L......TK.......

VK37 ...........E..A....E.V..K.K.S.GVG------------NN.H...............YA.R..............G---.-D.....RQ...........

VK38 ...........E..A......V..K.K.S.SV..-----------N-..L..........C...GTN...............G---.-D..................

VK39 ....S......E..A......V..Q.K.SYS.RN-----------N-..L........L......TNN........V.....G---.-D..................

VK40 ...........EA.V......V..K.K.S.SV.------------NW.N...............KT.N........V.....G---.-D..................

VK41 ..R........E..A......V..K.K.S.SV..-----------Y-..L..............YT.N......EL......G---.-D..................

VK42 ...........E..A......V..K.K.S.SV.------------KW.N...K.......N...KT.N..............G---.-D..................

VK43 ...........E..A......V..K.TAS.SVN------------NY.S....N..........YTNR.......W....E.G---.-D......Q...........

VK44 ...Q.......E....V..Q.V....KAS.SIG------------.YAH...K...........NAN...............G---.-D..L.......D.......

VK46 ...Q.......E..A.....QV..K.KAS.SL.YSSSWGS----YQQ.................GG...Q....AW......G---.-D..L...G.A.E.......

VK48 ...........E..A......V..Q.K.S.SI..-----------.R.NLH.............GTNN.L............G---.-D..................

VK49 ...........E..A......V..K.K.S.SV..-----------.R.H...............YA.R..............G---.-D..................

VK50 ...........E..A......V..K.KPS.SV..-----------.Y.H................TN......S........G---.-D..................

VK51 ...........E..AK.....V..K.TAS.SV..-----------W-.HL..............RTNN..............G---.-D.........D........

VK52 ...........E..A......V..K.K.S.SV..-----------.-.DL..............NTNN.A............G---.-D..........D.......

VK53 YC.........EA.IA.....V..K.K.S.SAG------------.S.H...............DTN...............G---.-D.....RQ..G........

VK54 ...........E..A......V..K.K.S.SV..-----------W-..L..............RA.N..............G---.-D..................

VK62 ...........E..A.........K.KAS.SI..-----------.-.H.....S........KYA...I............G---.-D......Q...E.......

**Ig**_κ_**V family2** (7 members)

<------------FR1------------><------CDR1-----><------FR2 ------>CDR<-----------------FR3------------------>

VK21 .......N.............A..T...V.YSG.-----------N-.......S..P......S.N.............R..---.-...L......V........

VK22 .N.........E......Q..V..T..A......-----------.-.......S.KS.....H.T...A...S.....AR.G---.-D..L...............

VK23 .......N...E..A.........T.KAV...G.-----------N-.......S..P.......TN.............R.G---.-D..L......V........

VK24 .N.....I...E....A.Q..V..T..A......-----------.-.......S.KS.......T...A...S.C...AR.G---N-D..LN..............

VK25 ...........E..A......V..K.K.S.SV..-----------.-.................GTNR.A............G---.-D.........P...A....

VK27 ...........E..A......V..K.K.S.SV..-----------Y-..L...............TN...............G---.-D..................

VK32 ....F......E..A....G.V..K.KAS.SV..-----------Y-.HL..M...........YTKVC..K...L....V.G---.DD.I........D.......

**Ig**_κ_**V family3** (2 members)

<------------FR1------------><------CDR1-----><------FR2 ------>CDR<-----------------FR3------------------>

VK55 ....NP...M.E..A.AL...V..K.RVS.SI..-----------N-.DL.H.....P......KTN...............G---.-D..........E.......

VK59 ...G.......E.........V..K.K.S.GIGR-----------D-.N........P.M...HDTNKG....LAQ......G---.-D.........V..S.....

**Ig**_κ_**V family4** (2 members)

<------------FR1------------><------CDR1-----><------FR2 ------>CDR<-----------------FR3------------------>

VK19 ...........K......................-----------.N.H...............S.........A........---.-.......H...........

VK45 ...........E..A..T.E.V..K.K.S.SV..-----------.-.................GTNR.A............G---.-DS....V....D.......

**Ig**_κ_**V family5** (17 members)

<------------FR1------------><------CDR1-----><------FR2 ------>CDR<-----------------FR3------------------>

VK1 ...Q.......E..A.....QV..K.KAS.SL.YSSSWGS----YQQ.................GG...Q....AW......G---.-D..L...G.A.E.......

VK2 ....R....A.E..A...R.RV..K.KCS.SL.YSG---R-----EE................TFA..QQP...TW......G---M-D..L...S...E.......

VK3 ...Q.......E..A.....QV..K.KAS.SL.YTFSDSNTPKYYHH.................GG...Q....A.......G---.-D..L...G.A.E.......

VK4 ....R......E..AP.L...V..K.KCS.SL..AG---K-----EF.G..............TFA...QP...A.......G---.-D..L...S...E.......

VK5 ...Q.......E..A.....RV..K.KAS.SL.YSSSGST----YQL.H...............DA...Q....A.......G---.-D..L...S...E.D.....

VK6 ...A.......Q..A.........K.K.S.SL.YSG---R-----EE.................NAV..Q....A.......G---.-D..L...H...E.......

VK7 ...Q.......E........RV..K.KAS.SL..RIISSS----YEQ.................LG...Q....A.......G---.-D..L...S...E.......

VK8 ...A.......Q..A...R.....K.K.S.SL.YSG---R-----EE............E....DAV..Q....A.......G---.-D..L...H...E.......

VK9 ...Q.......E..A.....RV..K.KAS.SL.YSSSGRT----YQG.................DAV..Q....A.......G---.-D..L...S...E.......

VK10 ....R......E..A.F.......K.KCS.SL..AG---K-----EF................TFA...QP...A.......G---.-D.IL.......E...H...

VK12 ...Q...R...E..A.....RV..K.KAS.SL.----YSG----NEY.................GG...Q....A.......G---.-D..L...S...E.......

VK13 ...Q.......E..A.....QV..K.KAS.SF.YSS--------YQV.................RA..QQ....A.......G---.-D..L...S...E.......

VK14 ...Q.......E..A.....RV..K.KAS.SL.YSSSGST----YQL.H...............DA...Q....A.......G---.-D..L...S...E.......

VK15 ...Q.......E..A.....RV..K.KAS.SLA----SGS----YQL.................FG...Q....A.......G---.-D..L...S...E.......

VK16 ...Q.......E..A.....RV..K.KAS.SL.DSR--------YQY.............C...DAV..Q....A.......G---.-D..L...S...E.......

VK17 ...Q.......E..A.....RV..K.KAS.SL.YSSSGST----YQL.................NAV..Q....A.......G---.-D..L...S...E.......

VK18 ...QA...........F...RV....RAS.SIKATS-------EV.Y.N..............RYA................G---.-D.............A....

**Ig**_κ_**V family6** (2 members)

<------------FR1------------><------CDR1-----><------FR2 ------>CDR<-----------------FR3------------------>

VK47 ...Q.....P....SDPV.GR...T....AR..------------NC.NR.H.....T......YA.ILQA...A..I.....---.-.V.....N...D..A.N..

VK58 ...Q.......E....PV.G.V..T.KAS.SV.------------.C.N........V.N....YA..LQ....AW......G---.-D......S...D..A....

**Ig**_κ_**V family7** (2 members)

<------------FR1------------><------CDR1-----><------FR2 ------>CDR<-----------------FR3------------------>

VK57 AQ.Q...M.........V.E....QS.S.RG.G------------KE.S....N..........SGN-HY..N..L..P....---.-...P...GF..E.T.....

VK61 AQ.Q.......E..A..V.E.V..Q.R.SRSIG------------.E.S..............N.V.-.Y........P...G---.-D..L...G.A.E.......

**Ig**_κ_**V family8** (1 member)

<------------FR1------------><------CDR1-----><------FR2 ------>CDR<-----------------FR3------------------>

VK34 .....A.............G............G.-----------N-..L..........Q...G.NN...............---.-..........VD.......

**Ig**_κ_**V family9** (1 member)

<------------FR1------------><------CDR1-----><------FR2 ------>CDR<-----------------FR3------------------>

VK36 ...........................T......-----------N-.NLH.............G..N........V......---.-...................

**Ig**_κ_**V family10 (1 member)**

<-----------FR1------------><------CDR1-----><------FR2 ------>CDR<-----------------FR3------------------>

VK56 LN.........A.......E.......P..P.G------------NE.N..LF.G.AR.....HHA...IP...E..N..H.S---.-.V....RG..ME.......

**Ig**_κ_**V family11** (1 member)

<------------FR1------------><------CDR1-----><------FR2 ------>CDR<-----------------FR3------------------>

VK60 ...........A..TAL..E....Q........------------DD..LLLF.S..T.....H.GNN.V..........Y..---.-......RG..VE.......

**Ig**_κ_**V family12** (1 member)

<------------FR1------------><------CDR1-----><------FR2 ------>CDR<-----------------FR3------------------>

VK11 ...Q........C.......R............HIR.EI.----.QE.................LG...Q....A........---.-...L...S...E.......
