# Supplementary material for: A Comprehensive Analysis of the Phylogeny, Genomic Organization and Expression of Immunoglobulin Light Chain Genes in Alligator sinensis, an Endangered Reptile Species
Source: PLoS One. 2016 Feb 22;11(2):e0147704. doi: 10.1371/journal.pone.0147704 (PMC4762898; doi:10.1371/journal.pone.0147704)
Supplement: S7 Appendix — (DOCX) [file pone.0147704.s007.docx]

**Sequence of the C region chimeras in the cDNA clones**

**LV2-8(C3+C2)**

**LV2-8(C3+C2)**: GACAGCCAAAGGCCTCTCCTACCGTCCACCTCTTCCCTCCCTCCTCGGAACAGGTCAACAGCAAGAGCCAGGCCACGCTGGTGTGTCTGATGGACAGCTTTTACCCCAGCTCAGTCCAGGTCACCTGGAAAGCTGATGGCACAACCATGTCCAGTGGAGTGGAGACCACCAAACCATCCAAACAGAGCGACAACAAGTACATGGCCAGC

**C3_λ_.seq:**  ...............................................................................................G...........G....G.............................C...........C................CG..G..............................G..

**C2_λ_.seq:** .........................................................G..........A............................................................................................................................................

**LV2-11(C2+C1)**

**LV2-11(C2+C1)**: GACAGCCAAAGGCCTCTCCTACCGTCCACCTCTTCCCTCCCTCCTCGGAACAGGTCAGCAGCAAGAGCAAGGCCACGCTGGTGTGTCTGATGGACAGCTTTTACCCCAGCTCAGTCCAGGTCACCTGGAAAGCTGATGGCACCCCCATCACCAGTGGAGTGGAGACCACCAAGCCGTCCAAACAGAGCGACAACAAGTACATGGCCAGC

**C2_λ_.seq**: ..............................................................................................................................................AA....GT......................A..A.................................

**C1_λ_.seq**: ......................A..G..............A.........G..A.....................................A...G...........G....G....T.........................................................C.................................

**LV2-38(C3+C1)**

**LV2-38(C3+C1)**: GACAGCCAAAGGCCTCTCCTACCGTCCACCTCTTCCCTCCCTCCTCGGAACAGGTCAACAGCAAGAGCCAGGCCACGCTGGTGTGTCTGATGGACGGCTTTTACCCCGGCTCGGTCCAGGTCACCTGGAAAGCTGATGGCACCACCATGTCCAGTGGAGTGGAGACCACCAAGCCCTCCAAACAGAGCGACAACAAGTACATGGCCAGC

**C3_λ_.seq**: ..........................................................................................................................................................C................C...G..............................G..

**C1_λ_.seq**: ......................A..G..............A.........G..A...G..........A......................A.........................T.........................C....CA...........................................................

**LV6-91(C2+C1)**

**LV6-91(C2+C1**): GACAGCCAAAGGCCTCTCCTACCGTCCACCTCTTCCCTCCCTCCTCGGAACAGGTCAGCAGCAAGAGCAAGGCCACGCTGGTGTGTCTGATGGACAGCTTTTACCCCAGCTCAGTCCTGGTCACCTGGAAAGCTGATGGCACCCCCATCACCAGTGGAGTGGAGACCACCAAGCCGTCCAAACAGAGCGACAACAAGTACATGGCCAGCAGCTACCTGTCGCTG

**C2_λ_.seq**: .....................................................................................................................A........................AA....GT......................A..A................................................

**C1_λ_.seq**: ......................A..G..............A.........G..A.....................................A...G...........G....G..............................................................C................................................

**LV6-91(C2+C1)**: AGCGCCTCCGCCTGGAAGAGCCACGAGACCTACAC

**C2_λ_.seq**: ..........A........................

**C1_λ_.seq**: ...................................

**LV-11(C3+C1)**

**LV-11(C3+C1)**: GACAGCCAAAGGCCTCTCCTACCGTCCACCTCTTCCCTCCCTCCTCGGAACAGGTCAACAGCAAGAGCCAGGCCACGCTGGTGTGTCTGATGGACGGCTTTTACCCCGGCTCGGTCCAGGTCACCTGGAAAGCTGATGGCACCCCCATCACCAGTGGAGTGGAGACCACCAAGCCGTCCAAACAGAGCGACAACAAGTACATGGCCAGCAGCTACCTGTCGCTG

**C3_λ_.seq**: ...............................................................................................................................................A....GT....C................C..................................G.................

**C1_λ_.seq**: ......................A..G..............A.........G..A...G..........A......................A.........................T.........................................................C................................................

**LV-11(C3+C1)**: AGCGCCTCCGACTGGAAGAGCCACGAGACCTACACCTGTCAGGTGACGCACGACGGGAAGAGCATCGAGAAGACCC

**C3_λ_.seq**: ............................................................................

**C1_λ_**.seq: .........C..................................................................

**LV-14(C4+C1)**

**LV-14(C4+C1)**: GACAGCCAAAGGCCTCTCCTACAGTGCACCTCTTCCCTCCATCCCCGGAAGAGATCACCAGCAAGAGCAAGGCCACGCTGGTGTGTCTGATGGACGGCTTTTACCCCGGCTCGGTCCAGGTCACCTGGAAAGCTGATGGCACCACCATGTCCAGCGGAGTGGAGACCACCAAGCCCTCCAAACAGAGCGACAACAAGTACATGGCCAGCAGCTACCTGTCGCTG

**C4_λ_.seq**: ...........................................................................................................................................................................C...G................................................

**C1_λ_**.seq: ............................................T............G.................................A.........................T.........................C....CA....T.....................................................................

**LV-14(C4+C1)**: AGCGCCTCCGCCTGGAAGAGCCACGAGACCTACACCTGTCAGGTGACGCACGACGGGAAGAGCATCGAGAAGACCCT

**C4_λ_**.seq: .............................................................................

**C1_λ_**.seq: .............................................................................

**LV-56(C3+C1)**

**LV-56(C3+C1)**: GACAGCCAAAGGCCTCTCCTACCGTCCACCTCTTCCCTCCCTCCTCGGAACAGGTCAACAGCAAGAGCCAGGCCACGCTGGTGTGTCTGATGGACGGCTTTTACCCCGGCTCGGTCCAGGTCACCTGGAAAGCTGATGGCACCACCATCACCAGTGGAGTGGAGACCACCAAGCCGTCCAAACAGAGCGACAACAAGTACATGGCCAGCAGCTACCTGTCGCT

**C3_λ_.seq**: ....................................................................................................................................................GT....C................C..................................G................

**C1_λ_.seq**: ......................A..G..............A.........G..A...G..........A......................A.........................T.........................C...............................C...............................................

**LV-56(C3+C1)**: GAGCGCCTCCGCCTGGAAGAGCCACGAGACCTACACCTGTCAGGTGACGCACGACGGGAAGAGCATCGAGAAGACCCT

**C3_λ_.seq**: ...........A..................................................................

**C1_λ_.seq**: ...............................................................................
