# Supplementary material for: A Comprehensive Analysis of the Phylogeny, Genomic Organization and Expression of Immunoglobulin Light Chain Genes in Alligator sinensis, an Endangered Reptile Species
Source: PLoS One. 2016 Feb 22;11(2):e0147704. doi: 10.1371/journal.pone.0147704 (PMC4762898; doi:10.1371/journal.pone.0147704)
Supplement: S8 Appendix — The letter in the middle indicates N/P nucleotides. The column “N+P” indicates the total nucleotide length of the N and P nucleotides, and the column “CDR3” indicates the codon numbers. The column “Deletions in 3’ end of Vλ” indicates the number of nucleotides deleted by exonuclease activity at the 3’ end of Vλ, and the column “Deletions in 5’ end of Vλ” indicates the number of nucleotides deleted by exonuclease activity at the 5’ end of Jλ. Germline sequences of each Vλ gene segment are shown above the cDNA clones in bold, and the CDR3 is also underlined. (DOCX) [file pone.0147704.s008.docx]

**V-J junctions of the λ chain genes**

N+P CDR3 Deletions in Deletions in

3’ end of V_λ_ 5’ end of J_λ_

Y Y C Q V W D S D S S T H

V_λ_2+J1 TATTACTGCCAGGTGTGGGACAGCGACAGCAGCACTCAC CGTATGTCTTCGGCGGCGGAACCCAGCTGACCGTCCTCG **J1**

LV5-4 ....................T......G......----- .......................................  0 11 5 0

Y Y C A V W H S D A C

V_λ_3+J1 TATTACTGTGCTGTGTGGCACAGTGATGCTTGT CGTATGTCTTCGGCGGCGGAACCCAGCTGACCGTCCTCG J1

LV5-53 .....T................A.....G.... -----..................................  0 9 0 5

LV2-32 ..................T..G......----- TA --..................................... 2 9 5 2

Y Y C Q V W D S S K A

V_λ_11+J1 TATTACTGCCAGGTGTGGGATAGCAGCAAGGCT CGTATGTCTTCGGCGGCGGAACCCAGCTGACCGTCCTCG **J1**

LV5-74 ..............A.......C..A....... -----..................................  0 9 0 5

V_λ_11+J3 TATTACTGCCAGGTGTGGGATAGCAGCAAGGCT GGCTACCTTCGGAGGAGGAACCCGGCTGACCGTCCTCG **J3**

LV5-6 ....T........C........A..C.....-- --..........TC........................ 0 9 2 2

Y Y C Q V W D S S S S Q A

V_λ_12+J1 TATTACTGCCAGGTGTGGGACAGCAGCAGTTCTCAGGCT CGTATGTCTTCGGCGGCGGAACCCAGCTGACCGTCCTCG J1

LV-2 .......................T.C..........--- --........T............................ 0 11 3 2

LV5-24 ......................C............---- ----................................... 0 10 4 4

LV5-67 .........................A..CA.......-- ---....................................  0 11 2 3

LV6-102 ..............A...C...C.C.............. -----.................................. 0 11 0 5

V_λ_12+J2 TATTACTGCCAGGTGTGGGACAGCAGCAGTTCTCAGGCT GTGGGTGTTCGGAGCAGGAACCCAGCTGACCGTCCTTG **J2**

LV5-49 ...........C.........TC...T.C.........- C ----.........C....C................... 1 11 1 4

V_λ_12+J3 TATTACTGCCAGGTGTGGGACAGCAGCAGTTCTCAGGCT GGCTACCTTCGGAGGAGGAACCCGGCTGACCGTCCTCG **J3**

LV2-43 .............C.............G........--- CT ---.........GC........................ 2 11 3 3

LV6-98 .....................G................. ----................G................. 0 11 0 4

Y Y C V A A G G G A S

V_λ_14+J2 TATTATTGTGTGGCAGCAGGTGGTGGTGCGTCTC GTGGGTGTTCGGAGCAGGAACCCAGCTGACCGTCCTTG J2

LV5-58 ..C..........T.A......A..A.A...--- --....T.....C......................... 0 9 3 2

Y Y C V A A G G G A S

V_λ_16+J2 TATTATTGTGTGGCAGCAGGTGGTGGTGCGTCT GTGGGTGTTCGGAGCAGGAACCCAGCTGACCGTCCTTG J2

LV6-91 ...........................T...-- --....................................  0 9 2 2

V_λ_16+J4 TATTATTGTGTGGCAGCAGGTGGTGGTGCGTCT GTGGGTGTTCGGAGGAGGAACCCAGCTGACCGTCCTTG J4

LV6-59 ..............G.......A........-- --.......................T............  0 9 2 2

Y Y C Q V W D S S S D L P

V_λ_17+J1 TATTACTGCCAGGTGTGGGACAGCAGCAGTGATCTGCCA CGTATGTCTTCGGCGGCGGAACCCAGCTGACCGTCCTCG **J1**

LV5-56 ..............C.......A.GA..A.......--- AA -..................G................... 2 12 3 1

LV4-1 ......................A.CA..A.A..G...-- ....................................... 0 12 2 0

LV5-27 ......................T.GA..A....A..--- --........T..T............T....A.......  0 11 3 2

LV6-61 .....................G...AGG.......---- -...................................... 0 11 4 1

LV5-51 .T......................G..........---- T --......................G.............. 1 11 4 2

V_λ_17+J2 TATTACTGCCAGGTGTGGGACAGCAGCAGTGATCTGCCA GTGGGTGTTCGGAGCAGGAACCCAGCTGACCGTCCTTG **J2**

LV5-20 ........................CT............- ......................................  0 12 1 0

V_λ_17+J4 TATTACTGCCAGGTGTGGGACAGCAGCAGTGATCTGCCA GTGGGTGTTCGGAGGAGGAACCCAGCTGACCGTCCTTG **J4**

LV6-64 ........................GA..A......---- ......................................  0 11 4 0

Y Y C S M W V S S S S Q A

V_λ_20+J1 TATTACTGCAGCATGTGGGTTAGCAGCAGTTCTCAGGCA CGTATGTCTTCGGCGGCGGAACCCAGCTGACCGTCCTCG **J1**

LV-39 ....................................--- --..................................... 0 11 3 2

LV5-47 ........TT..G.....C...CT...G.......---- A --......................TA............. 1 11 4 2

V_λ_20+J3 TATTACTGCAGCATGTGGGTTAGCAGCAGTTCTCAGGCA GGCTACCTTCGGAGGAGGAACCCGGCTGACCGTCCTCG **J3**

LV6-34 ...C......A.......C.................... ----.................................. 0 11 0 0

Y Y C Q V W D S S S D L P

V_λ_21+J1 TATTACTGCCAGGTGTGGGACAGCAGCAGTGATCTGCCA CGTATGTCTTCGGCGGCGGAACCCAGCTGACCGTCCTCG **J1**

LV5-55 ..............T........G.A.....G...---- -..T..C................................ 0 11 4 1

V_λ_21+J3 TATTACTGCCAGGTGTGGGACAGCAGCAGTGATCTGCCA GGCTACCTTCGGAGGAGGAACCCGGCTGACCGTCCTCG **J3**

LV6-84 .............................---------- ...................................... 0 9 10 0

Y H C S M W V S S S S P P

V_λ_22+J1 TATCACTGCAGCATGTGGGTTAGCAGCAGTTCTCCGCCT CGTATGTCTTCGGCGGCGGAACCCAGCTGACCGTCCTCG **J1**

LV6-76 ..................C..GA.GT...........-- ....................................... 0 12 2 0

V_λ_22+J2 TATCACTGCAGCATGTGGGTTAGCAGCAGTTCTCCGCCT GTGGGTGTTCGGAGCAGGAACCCAGCTGACCGTCCTTG **J2**

LV6-1 ....................................... A --.................................... 1 12 0 2

V_λ_22+J3 TATCACTGCAGCATGTGGGTTAGCAGCAGTTCTCCGCCT GGCTACCTTCGGAGGAGGAACCCGGCTGACCGTCCTCG **J3**

LV2-36 ...T......T.............GC..C.........- ...................................... 0 12 1 0

Y Y C S M W V S S S S Q A

V_λ_25+J1 TATTACTGCAGCATGTGGGTTAGCAGCAGTTCTCAGGCA CGTATGTCTTCGGCGGCGGAACCCAGCTGACCGTCCTCG **J1**

LV5-23 ..........C.........................--- --..................................... 0 11 3 2

Y Y C A A Y D M D T D T P

V_λ_28+J1 TATTACTGTGCAGCTTATGATATGGACACTGATACACCT CGTATGTCTTCGGCGGCGGAACCCAGCTGACCGTCCTCG **J1**

LV2-7 .........TT..T......A.A............---- T --......................C.....T........ 1 11 4 2

LV2-39 ....................C...........C.----- ....................................... 0 11 5 0

LV6-54 ...................TC...........C..---- -...................................... 0 11 4 1

LV6-11 ..........GC.G................A.....--- GT ----................................... 2 11 3 4

LV5-26 ..........T.T..........C....T....T...-- ...................G................... 0 12 2 0

LV-18 ................T...................... AC -------......A..A.......G.............. 2 11 0 7

LV5-17 .........T..T........G...C.T....G...... -----.................................. 0 11 0 5

V_λ_28+J2 TATTACTGTGCAGCTTATGATATGGACACTGATACACCT GTGGGTGTTCGGAGCAGGAACCCAGCTGACCGTCCTTG **J2**

LV5-8 ..........T..G....TT...............---- .............A.......T................ 0 11 4 0

LV2-11 ...............C..CC.....T...A...G..... A --..C.........T...G................... 1 12 0 2

Y Y C A A Y D M D T D T P

V_λ_28+J3 TATTACTGTGCAGCTTATGATATGGACACTGATACACCT GGCTACCTTCGGAGGAGGAACCCGGCTGACCGTCCTCG **J3**

LV5-5 .............T....C..............T.C... ----.................................. 0 11 0 4

LV6-18 ..................A.........T....------ GT ---..T..................T............. 2 10 6 3

V_λ_28+J4 TATTACTGTGCAGCTTATGATATGGACACTGATACACCT GTGGGTGTTCGGAGGAGGAACCCAGCTGACCGTCCTTG **J4**

LV6-53 ............C......C...............---- .....C..............................A. 0 11 4 0

LV6-107 ..........T.A........T.......C....----- T ...................................... 1 11 5 0

Y Y C A A Y D A T S D T P

V_λ_29+J1 TATTACTGTGCAGCTTATGATGCTACCAGTGATACACCT CGTATGTCTTCGGCGGCGGAACCCAGCTGACCGTCCTCG **J1**

LV-64 ......................T.C..........---- T --..................................... 1 11 4 2

V_λ_29+J2 TATTACTGTGCAGCTTATGATGCTACCAGTGATACACCT GTGGGTGTTCGGAGCAGGAACCCAGCTGACCGTCCTTG **J2**

LV2-2 ........C.GT.G.....CA.G...T.AGAC.C..--- -............C....................G... 0 11 3 1

V_λ_29+J3 TATTACTGTGCAGCTTATGATGCTACCAGTGATACACCT GGCTACCTTCGGAGGAGGAACCCGGCTGACCGTCCTCG **J3**

LV2-8 ........--....A.G..G-.T..G...CAG.T.---- TCA ...................................... 3 11 4 0

Y Y C A A Y D M D S N T P

V_λ_31+J2 TATTACTGTGCAGCTTATGATATGGACAGCAACACTCCT GTGGGTGTTCGGAGCAGGAACCCAGCTGACCGTCCTTG **J2**

LV2-16 .............G..............TTG.A..---- ...................................... 0 11 4 0

Y Y C S T Y D T G S S S P

V_λ_37+J2 TATTACTGCAGCACTTATGATACTGGCAGTAGCTCTCCA GTGGGTGTTCGGAGCAGGAACCCAGCTGACCGTCCTTG **J2**

LV6-6 .....T...G.TGG.....G..G.CC..C..A.AG..-- --....A...............................  0 11 2 2

LV-10 .........G.TGG..T..CCGG.A.T.AG.---..... -............A....................G... 0 11 0 1

LV-61 .T.......G.T.....C.C.GG.A.T.AC.-------- CT -.T............G........C............. 2 10 8 1

LV6-46 ....TT..TG.T.....C...GG..A..C...T..---- ......C............................... 0 11 4 0

V_λ_37+J3 TATTACTGCAGCACTTATGATACTGGCAGTAGCTCTCCA GGCTACCTTCGGAGGAGGAACCCGGCTGACCGTCCTCG **J3**

LV2-10 .....T..T..TGT...C.T.....A.....AG.....- ---................T.................A  0 11 1 3

LV6-56 .........G.T.G.....---G..AT...GA.A....- ---...A............................... 0 10 1 3

V_λ_37+J4 TATTACTGCAGCACTTATGATACTGGCAGTAGCTCTCCA GTGGGTGTTCGGAGGAGGAACCCAGCTGACCGTCCTTG **J4**

LV5-66 .........G.TGG.....---...AT.A..A.A...-- --.....................C.............. 0 10 2 2

LV-25 .........G.TGG....A---....T....A.A..... -.....A............................... 0 11 0 1

Y Y C S A Y A G S G F P

V_λ_38+J1 TATTATTGCAGTGCTTATGCTGGTAGTGGCTTTCCA CGTATGTCTTCGGCGGCGGAACCCAGCTGACCGTCCTCG **J1**

LV5-41 ............A.....T..TCC.C.A...----- .......................................  0 10 5 0

LV5-45 ..................................-- ....................................... 0 11 2 0

LV5-76 ...................G................ --..................................... 0 11 0 2

LV6-51 .....C......T.....A....C...--------- CATA ...................TG.................. 4 10 9 0

V_λ_38+J2 TATTATTGCAGTGCTTATGCTGGTAGTGGCTTTCCA GTGGGTGTTCGGAGCAGGAACCCAGCTGACCGTCCTTG **J2**

LV5-13 ............A.....A....C...--------- TATACA -.............A....................... 6 10 9 1

LV5-34 .....C............A....C...--------- TACTC ...................................... 5 10 9 0

LV5-64 .............G.............TC.CA...- ....A...................T.........T.A. 0 11 1 0

LV6-33 ........TT........A....C...TAT.C.... -..............G.........G.C.......... 0 11 1 1

LV-21 ........T....AA.................---- ......................................  0 10 4 0

LV6-41 ........T...A.....A....C...TAT.----- C ..............C..........G............  1 10 5 0

V_λ_38+J3 TATTATTGCAGTGCTTATGCTGGTAGTGGCTTTCCA GGCTACCTTCGGAGGAGGAACCCGGCTGACCGTCCTCG **J3**

LV5-60 ...................TA.A.......A..--- -...GGT.................T............. 0 10 3 1

Y Y C I A Y D S G S N S L

V_λ_39+J1 TATTACTGCATCGCTTATGATAGTGGCAGTAACTCTCTG CGTATGTCTTCGGCGGCGGAACCCAGCTGACCGTCCTCG **J1**

LV2-3 .............G...........A..C.TC...---- A --..................................... 1 11 4 2

LV6-69 .....T....G.A...G.....C.....C..-------- GGA ....................................... 3 11 8 0

LV-36 .............T...........A..C......---- -...................................... 0 11 4 2

V_λ_39+J2 TATTACTGCATCGCTTATGATAGTGGCAGTAACTCTCTG GTGGGTGTTCGGAGCAGGAACCCAGCTGACCGTCCTTG **J2**

LV5-11 .....T...TG....C........CC.....TG..---- ......................................  0 11 4 0

LV6-8 .....T..T.G..G.C........CC....C.G..GT.- CTTT -------............................... 4 11 1 7

LV6-42 ....CT....G.A...---............CT....-- C ---.................................G. 1 11 2 0

V_λ_39+J3 TATTACTGCATCGCTTATGATAGTGGCAGTAACTCTCTG GGCTACCTTCGGAGGAGGAACCCGGCTGACCGTCCTCG **J3**

LV6-100 .....T....ATA.....C.......A........---- ......................................  0 11 4 0

V_λ_39+J4 TATTACTGCATCGCTTATGATAGTGGCAGTAACTCTCTG GTGGGTGTTCGGAGGAGGAACCCAGCTGACCGTCCTTG **J4**

LV-1 .....T....AT.......---.......G.G.....-- C ...................................... 1 11 2 0

LV5-62 .............G..............A..G...---- C -..................................... 1 11 4 1

LV-L2 ....TT....GT.........C............T..-- C ....C................................. 1 12 2 0

LV5-29 .........GGT.G.....C.G..---......A...-- C ---................................... 1 10 2 3

Y Y C L T A D I S S A S P

V_λ_40+J4 TATTACTGCCTCACTGCTGATATTAGCAGTGCTTCTCCA GTGGGTGTTCGGAGGAGGAACCCAGCTGACCGTCCTTG **J4**

LV6-82 .................C....C..C..C........-- --.......................T............  0 11 2 2

Y Y C S A Y D S G S S S P

V_λ_51+J1 TATTACTGCAGCGCTTATGATAGTGGCAGTAGCTCTCCA CGTATGTCTTCGGCGGCGGAACCCAGCTGACCGTCCTCG **J1**

LV6-24 .....T..T................C..C...------- -...................................... 0 10 7 1

V_λ_51+J2 TATTACTGCAGCGCTTATGATAGTGGCAGTAGCTCTCCA GTGGGTGTTCGGAGCAGGAACCCAGCTGACCGTCCTTG **J2**

LV2-14 ..........T..G........C............---- ...................................... 0 11 4 0

V_λ_51+J3 TATTACTGCAGCGCTTATGATAGTGGCAGTAGCTCTCCA GGCTACCTTCGGAGGAGGAACCCGGCTGACCGTCCTCG **J3**

LV6-99 .........CT.TG...C...G..TC....GC.A.---- .C...T..............G................. 0 11 4 0

LV-28 .........TT..............T..C.........- ---........C............C............. 0 11 1 3

LV-56 ...........T..........C..A....G.TC.---- ------................................ 0 9 4 6

V_λ_51+J4 TATTACTGCAGCGCTTATGATAGTGGCAGTAGCTCTCCA GTGGGTGTTCGGAGGAGGAACCCAGCTGACCGTCCTTG **J4**

LV5-70 ..........T..G........C............---- ......A...............................  0 11 4 0

LV6-110 .....T.....T..........................- ---..........CG....................... 0 11 1 3

Y Y C S A Y D S G I S S P

V_λ_54+J2 TATTACTGCAGCGCTTATGATAGTGGCATTAGCTCTCCA GTGGGTGTTCGGAGCAGGAACCCAGCTGACCGTCCTTG **J2**

LV5-21 .....T....A..T...........C..G......---- ..............GG......................  0 11 4 0

LV2-35 .....T.....T.TC.....AGAG.T.T..CA......- ...................................... 0 12 1 0

V_λ_54+J3 TATTACTGCAGCGCTTATGATAGTGGCATTAGCTCTCCA GGCTACCTTCGGAGGAGGAACCCGGCTGACCGTCCTCG **J3**

LV5-12 .....T.......T.......G......G...G..---- ---...................................  0 10 4 3

LV5-43 ............A.......C.C.....G..AT..---- ............C......................... 0 11 4 0

V_λ_54+J4 TATTACTGCAGCGCTTATGATAGTGGCATTAGCTCTCCA GTGGGTGTTCGGAGGAGGAACCCAGCTGACCGTCCTTG **J4**

LV2-17 ....T.....CT.T........TC.C......TG.---- ......................................  0 11 4 0

LV6-96 ...C.T.......T..---T..C.....G..A...---- C -..........C...G........C............. 1 10 4 1

LV5-16 ............A...T.....C.....G..A......- ---................................... 0 11 1 3

Y Y C S V W T G S A Q

V_λ_61+J1 TATTACTGTTCAGTGTGGACTGGGAGTGCTCAG CGTATGTCTTCGGCGGCGGAACCCAGCTGACCGTCCTCG **J1**

LV2-15 ............................----- ........C.............................. 0 9 5 0

LV6-90 ....T.......A.........AA...------ A ....................................... 1 9 6 0

LV-53 ...............C.........A.....-- ---.......................T............ 0 9 2 3

Y Y C L T S D S S S N

V_λ_67+J3 TATTACTGTTTAACATCTGACAGCAGCAGCAATGC GGCTACCTTCGGAGGAGGAACCCGGCTGACCGTCCTCG **J3**

LV5-40 ...........G..C.A......GGA.GA....C. C -.....T............................... 1 11 0 1

Y Y C V A W I G S A H

V_λ_70+J2 TATTACTGTGTTGCATGGATTGGGAGTGCTCAC GTGGGTGTTCGGAGCAGGAACCCAGCTGACCGTCCTTG **J2**

LV6-19 .............................---- ...................................... 0 9 4 0

V_λ_70+J4 TATTACTGTGTTGCATGGATTGGGAGTGCTCAC GTGGGTGTTCGGAGGAGGAACCCAGCTGACCGTCCTTG **J4**

LV2-42 .............................---- .........................T............  0 9 4 0

Y Y C S V W T G S A

V_λ_71+J4 TATTACTGTTCAGTATGGACTGGGAGTGCTTG GTGGGTGTTCGGAGGAGGAACCCAGCTGACCGTCCTTG **J4**

LV-29 .............CT................. ---...................................  0 9 0 3

Y Y C I V Y T G S S A W

V_λ_72+J4 TACTACTGTATTGTGTATACTGGCAGCAGTGCGTGGTG GTGGGTGTTCGGAGGAGGAACCCAGCTGACCGTCCTTG **J4**

LV5-52 .........C....T....................... ---................................... 0 11 3 3

Y Y C A V W H S D S L

V_λ_74+J2 TATTACTGTGCTGTCTGGCACTCTGACTCTCTT GTGGGTGTTCGGAGCAGGAACCCAGCTGACCGTCCTTG **J4**

LV6-14 .............................---- ..............G....................... 0 9 4 0

Y Y C A V Y T G S N P L

V_λ_76+J1 TACTACTGTGCTGTGTATACTGGCAGCAACCCACTG CGTATGTCTTCGGCGGCGGAACCCAGCTGACCGTCCTCG **J1**

LV-34 .....T......C....................... C ---.................................... 1 11 0 3

LV-42 ..........T..............A......GT.- ----...................................  0 10 1 4

V_λ_76+J3 TACTACTGTGCTGTGTATACTGGCAGCAACCCACTG GGCTACCTTCGGAGGAGGAACCCGGCTGACCGTCCTCG **J3**

LV-55 ...................A......G..T..---- ---................................... 0 9 4 3

V_λ_76+J4 TACTACTGTGCTGTGTATACTGGCAGCAACCCACTG GTGGGTGTTCGGAGGAGGAACCCAGCTGACCGTCCTTG **J4**

LV6-73 ..T..T........T......TAT..TT....---- AGTATTA -------............................... 7 10 4 7

LV5-31 .........................A.G....---- .........G............................ 0 10 4 0

Y Y C A V Y T G S S S A L R

V_λ_77+J2 TACTACTGTGCTGTGTATACTGGCAGCAGCAGTGCGTTACGA GTGGGTGTTCGGAGCAGGAACCCAGCTGACCGTCCTTG **J2**

LV-15 .........TT....C............A..A...A.----- G ...................................... 1 12 5 0

V_λ_77+J4 TACTACTGTGCTGTGTATACTGGCAGCAGCAGTGCGTTACGA GTGGGTGTTCGGAGGAGGAACCCAGCTGACCGTCCTTG **J4**

LV6-28 ..............A...........G.A......A.....- ---...A............................... 0 12 1 3

LV5-19 ..............C...T.....GA......A.....---- .........................T............ 0 12 4 0

Y Y C M L W I G S T .

V_λ_78+J2 TATTACTGTATGTTATGGATTGGGAGTACTTAA GTGGGTGTTCGGAGCAGGAACCCAGCTGACCGTCCTTG **J2**

LV5-59 ...................C.........---- ...................................... 0 9 4 0

V_λ_78+J4 TATTACTGTATGTTATGGATTGGGAGTACTTAA GTGGGTGTTCGGAGGAGGAACCCAGCTGACCGTCCTTG **J4**

LV6-93 .........C.........C..A..-------- T ..................G................... 1 8 8 0

LV-14 ............G......C.........---- ...................................... 0 9 4 0

Y Y C A V H A G S S A W

V_λ_81+J4 TACTACTGTGCTGTACATGCTGGCAGCAGTGCGTGGTG GTGGGTGTTCGGAGGAGGAACCCAGCTGACCGTCCTTG **J4**

LV2-1 ..T........AC.....A.......TTA.A....... ---...C...............................  0 11 0 3

Y Y C A T W T G S A

V_λ_86+J4 TATTACTGTGCTACCTGGACTGGGAGTGCTTG GTGGGTGTTCGGAG.AGGAACCCAGCTGACCGTCCTTG **J4**

LV6-95 .............T..............---- G ....A................................. 1 9 4 0
