# Supplementary material for: A Comprehensive Analysis of the Phylogeny, Genomic Organization and Expression of Immunoglobulin Light Chain Genes in Alligator sinensis, an Endangered Reptile Species
Source: PLoS One. 2016 Feb 22;11(2):e0147704. doi: 10.1371/journal.pone.0147704 (PMC4762898; doi:10.1371/journal.pone.0147704)
Supplement: S9 Appendix — The letter in the middle indicates N/P nucleotides. The column “N+P” indicates the total nucleotide length of the N and P nucleotides, and the column “CDR3” indicates the codon numbers. The column “Deletions in 3’ end of Vκ” indicates the number of nucleotides deleted by exonuclease activity at the 3’ end of Vκ, and the column “Deletions in 5’ end of Jκ” indicates the number of nucleotides deleted by exonuclease activity at the 5’ end of Jκ. Germline sequences of each Vκ gene segment are shown above the cDNA clones in bold, and the CDR3 is also underlined. (DOCX) [file pone.0147704.s009.docx]

**V-J junctions of the κ chain genes**

N+P CDR3 Deletions in Deletions in

3’ end of V_κ_ 5’ end of J_κ_

Y Y C Q Q N Y G A P

V_κ_4+J4 TATTACTGTCAGCAGAATTATGGTGCACCTCT GTTCACTTTCGGCGGGGGGACCAAGGTGGAGATAAAAC **J4**

KV2-20 ................C........T...... ---................................... 0 9 0 3

Y Y C Q Q S Q S A P

V_κ_5+J1 TATTATTGTCAGCAGTCCCAGAGTGCTCCTCA GTGGACGTTCGGTCAAGGAACCAAGGTAGAAATCAAAC **J1**

KV3-44 ...............A...GC....G------ A -..................................... 1 8 6 1

KV2-40 .................T.......T...--- ...................................... 0 9 3 0

V_κ_5+J2 TATTACTGTCAGCAGAATTATGGTGCACCTCT GTACAATTTCGGCAAGGGGACCCGGGTAGAGATCAAAC J2

KV-21 .............................--- ...................................... 0 9 3 0

V_κ_5+J3 TATTATTGTCAGCAGTCCCAGAGTGCTCCTCA GTACACTTTCGGCAAGGGGACCCGAGTAGAAATCAAAC **J3**

KV-36 ......................C......--- ......................................  0 9 3 0

V_κ_5+J5 TATTATTGTCAGCAGTCCCAGAGTGCTCCTCA GATCACTTTTGGCAAGGGGACCAAGCTGGAGATAAAAC **J5**

KV-47 ....T..........A...GC....G...--- .................C.................... 0 9 3 0

V_κ_5+J6 TATTATTGTCAGCAGTCCCAGAGTGCTCCTCA GCTCACTTTCGGCAAAGGGACCAAGTTGGAGATAAAAC **J6**

KV-92 ...............A...GCG...G..---- --.................................... 0 8 4 2

Y Y C Q Q Y K S S P

V_κ_7+J1 TATTACTGTCAGCAGTACAAAAGCTCACCTCA GTGGACGTTCGGTCAAGGAACCAAGGTAGAAATCAAAC **J1**

KV2-2 .............................--- ...................................... 0 9 3 0

V_κ_7+J2 TATTACTGTCAGCAGTACAAAAGCTCACCTCA GTACAATTTCGGCAAGGGGACCCGGGTAGAGATCAAAC **J2**

KV-4(0601)................T.....C........- --..G................................. 0 9 1 2

Y Y C H Q S R S D P

V_κ_13+J1 TATTACTGTCACCAGTCCCGCAGTGATCCTCA GTGGACGTTCGGTCAAGGAACCAAGGTAGAAATCAAAC **J1**

KV3-9 ...............................- --G...C............................... 0 9 1 2

KV2-4 ..........................G....- GT ----........G........T................ 2 9 1 4

KV2-62 .............................--- ...................................... 0 9 3 0

V_κ_13+J2 TATTACTGTCACCAGTCCCGCAGTGATCCTCA GTACAATTTCGGCAAGGGGACCCGGGTAGAGATCAAAC **J2**

KV3-26 .............................--- ...................................... 0 9 3 0

V_κ_13+J6 TATTACTGTCACCAGTCCCGCAGTGATCCTCA GCTCACTTTCGGCAAAGGGACCAAGTTGGAGATAAAAC **J6**

KV2-37 ...............A...............- --......................A........T.... 0 9 1 2

Y Y C Q Q S Q S A P

V_κ_15+J1 TATTATTGTCAGCAGTCCCAGAGTGCTCCTCA GTGGACGTTCGGTCAAGGAACCAAGGTAGAAATCAAAC **J1**

KV2-50 ............................---- --.................................... 0 8 4 2

KV2-52 ....T...................A....--- .................TT................... 0 9 3 0

KV3-8 ..........TA.....A.......T...... ---................................... 0 9 0 3

KV-17 .........................T...--- ---G....................C............. 0 8 3 3

V_κ_15+J2 TATTATTGTCAGCAGTCCCAGAGTGCTCCTCA GTACAATTTCGGCAAGGGGACCCGGGTAGAGATCAAAC **J2**

KV-65 .............................TC- --.................................... 0 9 1 2

KV3-32 .............................--- ...................................... 0 9 3 0

KV-90 ...............G..........------ .....CC....................T.......... 0 8 6 0

V_κ_15+J3 TATTATTGTCAGCAGTCCCAGAGTGCTCCTCA GTACACTTTCGGCAAGGGGACCCGAGTAGAAATCAAAC **J3**

KV-54 .....C.......................... ---................................... 0 8 3 0

V_κ_15+J4 TATTATTGTCAGCAGTCCCAGAGTGCTCCTCA GTTCACTTTCGGCGGGGGGACCAAGGTGGAGATAAAAC **J4**

KV2-18 .............................--- ............G..........T.............. 0 9 3 0

KV2-63 ...........C.................--- ---...C..........................G.... 0 8 3 3

V_κ_15+J6 TATTATTGTCAGCAGTCCCAGAGTGCTCCTCA GCTCACTTTCGGCAAAGGGACCAAGTTGGAGATAAAAC **J6**

KV2-44 .............................--- ...................................... **0 9 3 0**

Y Y C Q Q G H S S P

V_κ_16+J1 TATTACTGTCAGCAGGGCCACAGCTCACCTCT GTGGACGTTCGGTCAAGGAACCAAGGTAGAAATCAAAC **J1**

KV2-6 .............................--- ...................................... 0 9 3 0

KV-58 ......................AT....---- --.................................... 0 8 4 2

Y Y C H Q Y R S S H

V_κ_17+J6 TATTACTGTCACCAGTACCGCAGCTCACATCA GCTCACTTTCGGCAAAGGGACCAAGTTGGAGATAAAAC **J6**

KV-27 ............................---- --...........C..................C..... 0 8 4 2

Y Y C H Q S R S T P

V_κ_18+J1 TATTACTGTCACCAGTCCCGTAGCACTCCTCA GTGGACGTTCGGTCAAGGAACCAAGGTAGAAATCAAAC **J1**

KV2-28 ......................A..G...--- ...................................... 0 10 3 0

KV2-22 ...............C......A......--- ---........................C.......... 0 8 3 3

KV-49 ....G......G...................- C ---................................... 1 9 1 3

V_κ_18+J2 TATTACTGTCACCAGTCCCGTAGCACTCCTCA GTACAATTTCGGCAAGGGGACCCGGGTAGAGATCAAAC **J2**

KV2-5 ..C................T.....G.....- --.................................... 0 9 1 3

KV-73 ......................A......--- ...................................... 0 9 3 0

V_κ_18+J3 TATTACTGTCACCAGTCCCGTAGCACTCCTCA GTACACTTTCGGCAAGGGGACCCGAGTAGAAATCAAAC **J3**

KV-19 .....................G....------ ...................................... 0 8 6 0

V_κ_18+J4 TATTACTGTCACCAGTCCCGTAGCACTCCTCA  GTTCACTTTCGGCGGGGGGACCAAGGTGGAGATAAAAC **J4**

KV-6(0601).........T...................... ...................................... 0 10 0 0

KV2-17 ............................. ..............................A...G... 0 9 3 0

V_κ_18+J6 TATTACTGTCACCAGTCCCGTAGCACTCCTCA GCTCACTTTCGGCAAAGGGACCAAGTTGGAGATAAAAC **J6**

KV2-34 .............................--- ...................................... 0 9 3 0

Y Y C Q Q Y S S S P L

V_κ_24+J4 TATTACTGTCAACAGTATAGCAGTTCCCCTCTCA GTTCACTTTCGGCGGGGGGACCAAGGTGGAGATAAAAC **J4**

KV-8 ..................GA.........----- ...................................... 0 9 5 0

Y Y C Q Q G S S G S

V_κ_26+J2 TATTACTGTCAGCAAGGTAGCAGCGGGTCT GTACAATTTCGGCAAGGGGACCCGGGTAGAGATCAAAC **J2**

KV2-1 ...........A........AC......-- --.................................... 0 8 2 2

Y Y C Q Q G H L M P

V_κ_29+J1 TATTACTGTCAGCAAGGCCACCTAATGCCTCT GTGGACGTTCGGTCAAGGAACCAAGGTAGAAATCAAAC **J1**

KV-1(0601)..........TA............C....--- ...................................... 0 9 3 0

Y Y C Q Q Y Y G S P

V_κ_32+J1 TATTACTGTCAACAGTATTATGGTAGCCCTCG GTGGACGTTCGGTCAAGGAACCAAGGTAGAAATCAAAC **J1**

KV2-25 ..............C........C..G...-- -...................................G. 0 9 2 1

KV-12 ...................T......T....- --.........................T.......G.. 0 9 1 2

Y Y C Q Q G T S F P

V_κ_34+J4 TATTACTGTCAGCAGGGTACTAGCTTCCCTCA GTTCACTTTCGGCGGGGGGACCAAGGTGGAGATAAAAC **J4**

KV3-13 .....T.............GG.TGA.T..--- .........................C............ 0 9 3 0

Y Y C Q Q Y Q S W P

V_κ_37+J1 TATTACTGTCAGCAGTATCAGAGCTGGCCTCT GTGGACGTTCGGTCAAGGAACCAAGGTAGAAATCAAAC **J1**

KV3-17 ................T.....CT.C.CC--- ....................................G. 0 9 3 0

Y Y C Q Q S Y S S

V_κ_38+J1 TATTACTGTCAGCAGAGTTATAGCTCTCC GTGGACGTTCGGTCAAGGAACCAAGGTAGAAATCAAAC **J1**

KV2-10 .........GGA..........ATA.C.. ...................................... 0 9 0 0

KV-25 .........GG..........GATAT... ---............................G...... 0 8 0 3

V_κ_38+J2 TATTACTGTCAGCAGAGTTATAGCTCTCC GTACAATTTCGGCAAGGGGACCCGGGTAGAGATCAAAC **J2**

KV-87 ...C.....GGC....A.....ATA.C.. ...................................... 0 9 0 0

V_κ_38+J3 TATTACTGTCAGCAGAGTTATAGCTCTCC GTACACTTTCGGCAAGGGGACCCGAGTAGAAATCAAAC **J3**

KV-6 .........GG...........ATA.C.. ...................................... 0 9 0 0

Y Y C Q Q Y Q S W P

V_κ_39+J1 TATTACTGTCAGCAGTATCAGAGCTGGCCTCT GTGGACGTTCGGTCAAGGAACCAAGGTAGAAATCAAAC **J1**

KV3-10 ................................ AG -----................................. 2 9 0 5

KV-9 ..................G............. ---...................................  0 9 0 3

KV2-48 ......................C......... TT -----......................T.......... 2 9 0 5

KV-24 ................T...C..........- G ---...................................  1 9 1 3

KV3-18 ..................G............. GG -----......................T.....T.... 2 9 0 5

KV2-41 ..............A.T..T..CT.CC..--- ...................................... 0 9 3 0

V_κ_39+J2 TATTACTGTCAGCAGTATCAGAGCTGGCCTCT GTACAATTTCGGCAAGGGGACCCGGGTAGAGATCAAAC **J2**

KV2-60 ................................ A ----..................................  1 9 0 4

V_κ_39+J3 TATTACTGTCAGCAGTATCAGAGCTGGCCTCT GTACACTTTCGGCAAGGGGACCCGAGTAGAAATCAAAC **J3**

KV3-30 ...........A..A..........CC..--- .........................C.....T...... 0 9 3 0

V_κ_39+J4 TATTACTGTCAGCAGTATCAGAGCTGGCCTCT GTTCACTTTCGGCGGGGGGACCAAGGTGGAGATAAAAC **J4**

KV-57 ................C............... ---------............................. 0 7 0 9

KV3-45 ....................C........... ---..G................................ 0 9 0 3

V_κ_39+J6 TATTACTGTCAGCAGTATCAGAGCTGGCCTCT GCTCACTTTCGGCAAAGGGACCAAGTTGGAGATAAAAC **J6**

KV2-42 ..............................-- -................................T....  0 9 2 1

Y Y C Q Q A S S P

V_κ_41+J1 TATTACTGTCAGCAGGCTAGTAGCCCTCC GTGGACGTTCGGTCAAGGAACCAAGGTAGAAATCAAAC **J1**

KV2-36 ...............AG.GC...TAGC.. ...................................... 0 9 0 0

V_κ_41+J2 TATTACTGTCAGCAGGCTAGTAGCCCTCC GTACAATTTCGGCAAGGGGACCCGGGTAGAGATCAAAC **J2**

KV2-45 ...............AG.TA....T..-- -..................................... 0 8 2 1

V_κ_41+J3 TATTACTGTCAGCAGGCTAGTAGCCCTCC GTACACTTTCGGCAAGGGGACCCGAGTAGAAATCAAAC **J3**

KV2-8 ...............AG.TA.GA.T.--- .....G........C................G...T.. 0 8 3 0

Y Y C Q Q Y G N T P

V_κ_42+J1 TATTACTGCCAGCAGTATGGTAATACCCCTCT GTGGACGTTCGGTCAAGGAACCAAGGTAGAAATCAAAC **J1**

KV2-67 ........TGG....AG.TAC........--- .................C......C....CT...GG.. 0 9 3 0

V_κ_42+J2 TATTACTGCCAGCAGTATGGTAATACCCCTCT GTACAATTTCGGCAAGGGGACCCGGGTAGAGATCAAAC **J2**

KV-44 ........TGG....AG.TT.C...A...--- ...T.G.....................C.......... 0 9 3 0

V_κ_42+J4 TATTACTGCCAGCAGTATGGTAATACCCCTCT GTTCACTTTCGGCGGGGGGACCAAGGTGGAGATAAAAC J4

KV-70 ........TGG....AG.TA.....T...--- ...................................... 0 9 3 0

KV-61 .....................C...T...... ---................................G.. 0 9 0 3

Y Y C Q Q Y Q S F P

V_κ_43+J1 TATTACTGTCAGCAGTATCAAAGTTTCCCTCC GTGGACGTTCGGTCAAGGAACCAAGGTAGAAATCAAAC **J1**

KV-20 ...............GG.G.C..........- --.................................... 0 9 1 2

KV-31 ...............GG.A....C.......- T ---.................G................. 1 9 1 3

V_κ_43+J3 TATTACTGTCAGCAGTATCAAAGTTTCCCTCC GTACACTTTCGGCAAGGGGACCCGAGTAGAAATCAAAC **J3**

KV-51 ..C......GG....AC.T.T.T..C...... ---................................... 0 9 0 3

V_κ_43+J4 TATTACTGTCAGCAGTATCAAAGTTTCCCTCC GTTCACTTTCGGCGGGGGGACCAAGGTGGAGATAAAAC **J4**

KV2-26 .........GG....AG...T.A......--- ...................................... 0 9 3 0

V_κ_43+J6 TATTACTGTCAGCAGTATCAAAGTTTCCCTCC GCTCACTTTCGGCAAAGGGACCAAGTTGGAGATAAAAC **J6**

KV-94 ...............GG.G.C........--- ........................C.....C....... 0 9 3 0

Y Y C Q Q G Y T P

V_κ_44+J2 TATTACTGTCAGCAGGGTTATACCCCTCT **GTACAAT**TTCGGCAAGGGGACCCGGGTAGAGATCAAAC **J2**

KV-32 ...............A......GTA.--- .....G................................ 0 8 3 0

V_κ_44+J3 TATTACTGTCAGCAGGGTTATACCCCTCT GTACACTTTCGGCAAGGGGACCCGAGTAGAAATCAAAC **J3**

KV3-23 ...............A.....GA.T.--- ....................................G.  0 8 3 0

Y Y C Q Q G N S F P

V_κ_49+J1 TATTACTGTCAGCAGGGTAACAGCTTCCCTCT GTGGACGTTCGGTCAAGGAACCAAGGTAGAAATCAAAC **J1**

KV-7 ...........C......GCT.TTAC...--- ...................................... 0 9 3 0

Y Y C G Q S S Y N P

V_κ_49+J4 TATTACTGTCAGCAGGGTAACAGCTTCCCTCT GTTCACTTTCGGCGGGGGGACCAAGGTGGAGATAAAAC **J4**

KV-50 ...........C......GCT..TAC...--- ...................................... 0 9 3 0

Y Y C Q Q G D S T P

V_κ_50+J1 TATTACTGTCAGCAGGGTGATAGTACCCCTCC GTGGACGTTCGGTCAAGGAACCAAGGTAGAAATCAAAC **J1**

KV-35 ...........C....C.......G......- --.................................... 0 9 1 2

KV-59 ...........T...........A........ ---................................... 0 9 0 3

V_κ_50+J2 TATTACTGTCAGCAGGGTGATAGTACCCCTCC GTACAATTTCGGCAAGGGGACCCGGGTAGAGATCAAAC **J2**

KV3-46 ..................TT...C.....--- .................C.................... 0 9 3 0

V_κ_50+J3 TATTACTGTCAGCAGGGTGATAGTACCCCTCC GTACACTTTCGGCAAGGGGACCCGAGTAGAAATCAAAC **J3**

KV-38 ..............................-- -..................................... 0 9 2 1

KV3-41 ........................G....--- ...................................... 0 9 3 0

KV-3 .....T.....A...TCCC.G.....T....- --......................C..C.......... 0 9 1 2

V_κ_50+J4 TATTACTGTCAGCAGGGTGATAGTACCCCTCC GTTCACTTTCGGCGGGGGGACCAAGGTGGAGATAAAAC **J4**

KV-23 ..........................T..--- ..............................C....... 0 9 3 0

KV3-22 .........................A...... ---................................... 0 9 0 3

Y Y C G Q S S Y N P

V_κ_51+J4 TATTACTGTGGGCAGAGCTCATATAACCCTTG GTTCACTTTCGGCGGGGGGACCAAGGTGGAGATAAAAC **J4**

KV-37 .................T.ATA...T...--- ...................................... 0 9 3 0

Y Y C Q Q H D D T P

V_κ_54+J3 TATTACTGCCAGCAGCATGATGATACCCCTCT GTACACTTTCGGCAAGGGGACCCGAGTAGAAATCAAAC **J3**

KV-26 ...............T................ ---................................... 0 9 0 3

KV-11 ...............T...G....GT...--- ...........C.............A............ 0 9 3 0

Y Y C Q Q C N Q W P

V_κ_58+J3 TACTACTGTCAGCAGTGTAACCAATGGCCTCC GTACACTTTCGGCAAGGGGACCCGAGTAGAAATCAAAC **J3**

KV-5(0601).............................--- ...................................... 0 9 3 0

KV3-16 ..................G....T.....--- T -..................................... 1 9 3 1

V_κ_58+J6 TACTACTGTCAGCAGTGTAACCAATGGCCTCC GCTCACTTTCGGCAAAGGGACCAAGTTGGAGATAAAAC **J6**

KV-89 .............................--- ...............C...................... 0 9 3 0
