# Supplementary figures and images for: A Comprehensive Analysis of the Phylogeny, Genomic Organization and Expression of Immunoglobulin Light Chain Genes in Alligator sinensis, an Endangered Reptile Species
Source: PLoS One. 2016 Feb 22;11(2):e0147704. doi: 10.1371/journal.pone.0147704 (PMC4762898; doi:10.1371/journal.pone.0147704)

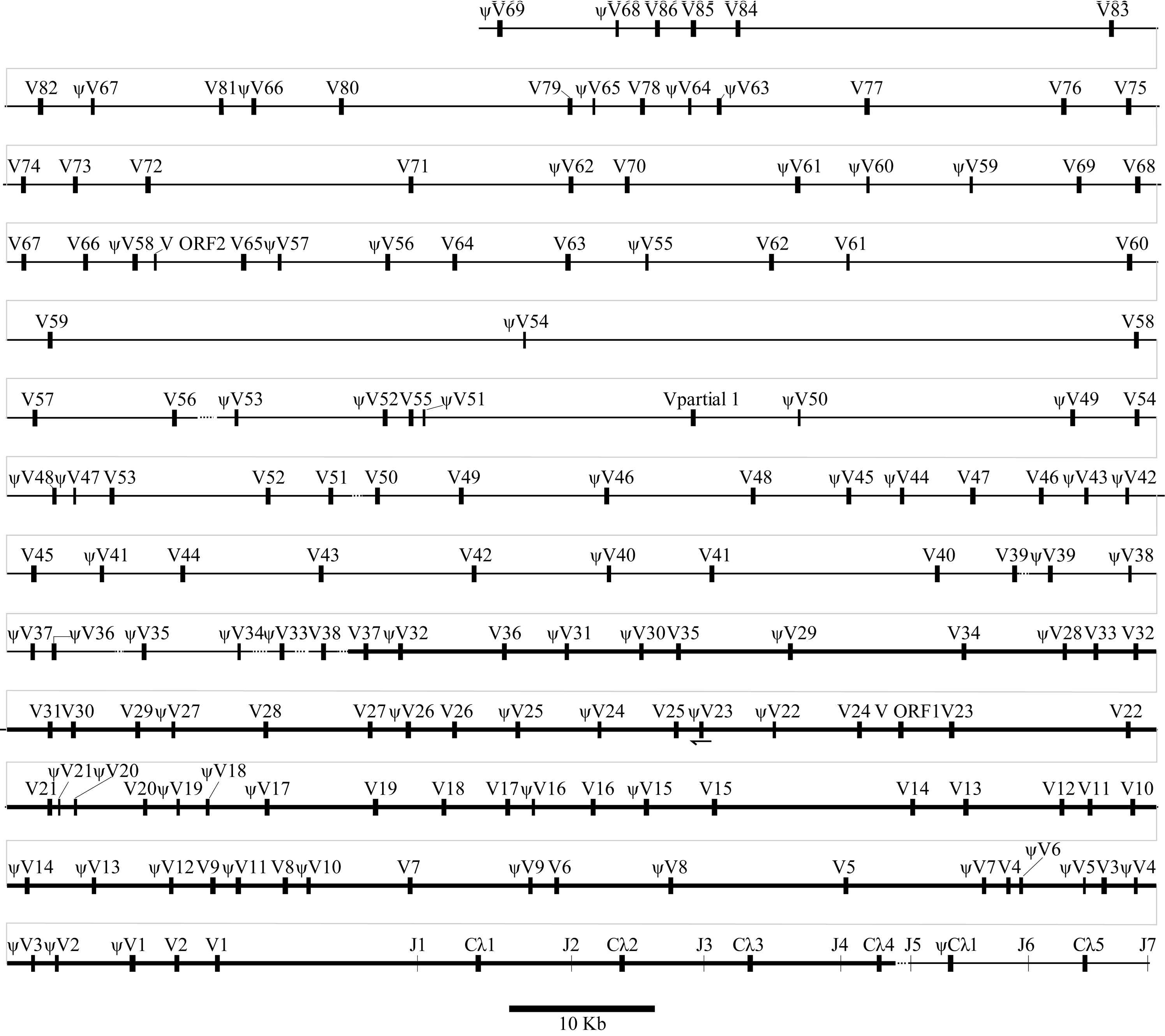

Supplement: S1 Fig — V: variable gene segments; ΨV: pseudo-variable gene segments; ORF: variable gene segments with open reading frames but with defects in splicing sites, RSS and/or regulatory elements, and/or changing the conserved amino acids, which have been suggested to lead to incorrect folding [69]; J: joining gene segments; C: constant region gene; ΨC: pseudo-constant region gene. Gaps between contigs are indicated by a dotted black line, and the sequences from BAC are indicated by a bold line. (TIF) [file pone.0147704.s010.tif]

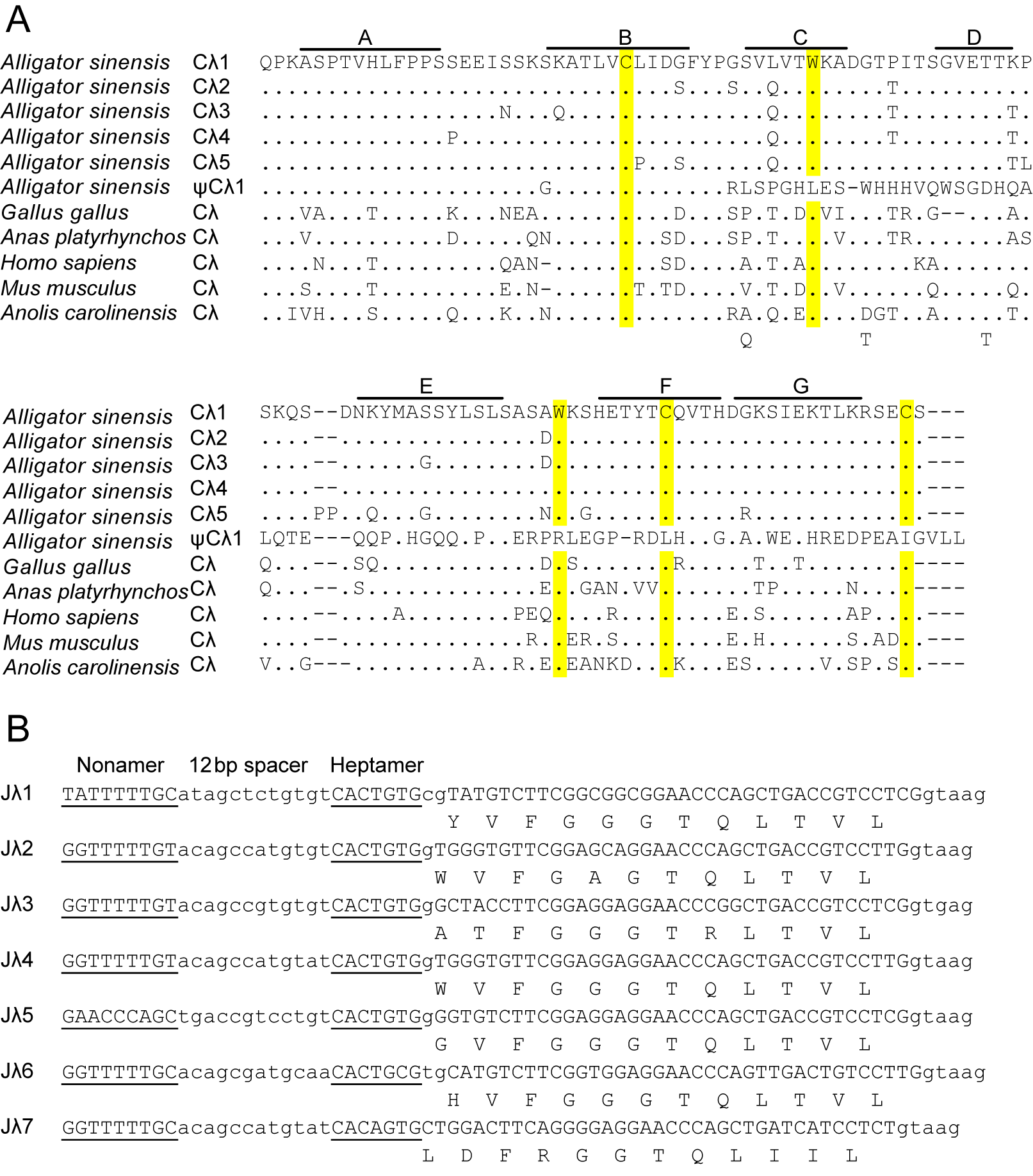

Supplement: S2 Fig — (A) Nucleotide and amino acid sequences of the seven Alligator sinensis Jλ segments. (B) Sequence comparison of the six Alligator sinensis Cλ genes with their counterparts in the Homo sapiens, Mus musculus, Gallus gallus, Anas platyrhynchos and Anolis carolinensis. In the alignment, dots indicate identical amino acids and A-G over the lines represent potential IgSF strands. The cysteine (C) and tryptophan (W) residues are shaded. (TIF) [file pone.0147704.s011.tif]

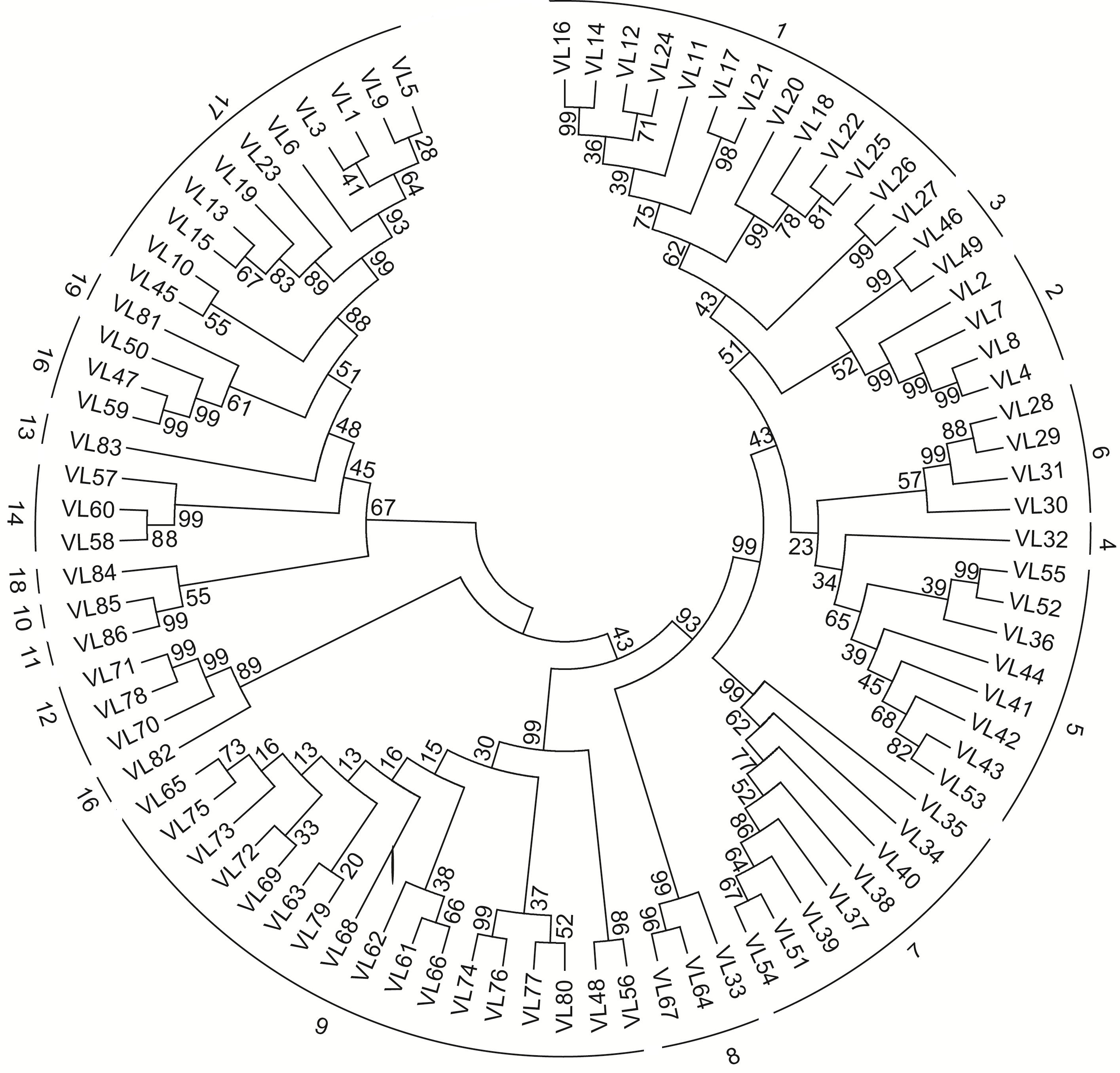

Supplement: S4 Fig — The tree is made by Neighbor-joining P-distance and pairwise deletions using MEGA6.0. (TIF) [file pone.0147704.s013.tif]

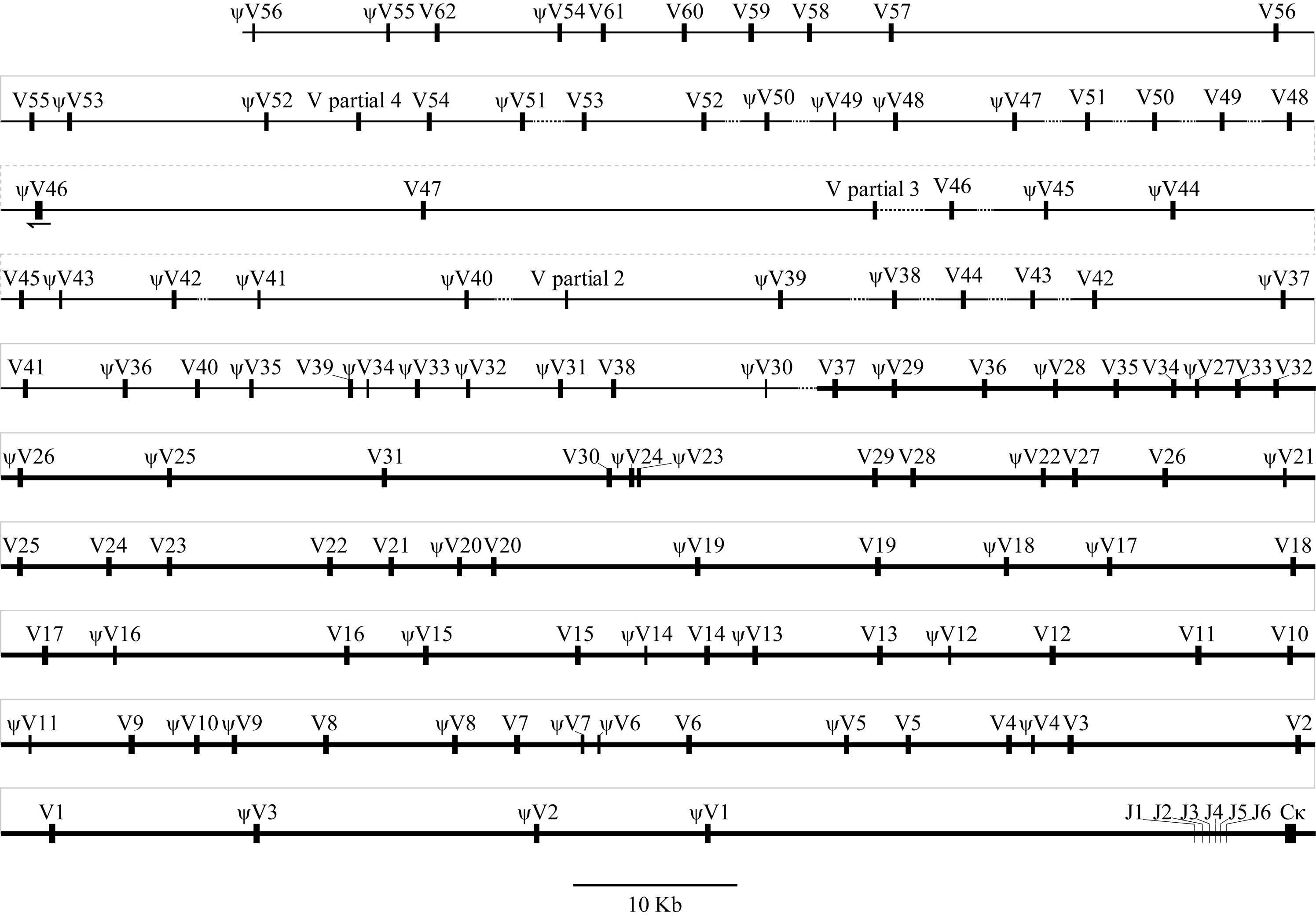

Supplement: S5 Fig — V: variable gene segments; ΨV: pseudo-variable gene segments; ORF: variable gene segments with open reading frames but with defects in splicing sites, RSS and/or regulatory elements, and/or changing the conserved amino acids, which have been suggested to lead to incorrect folding [69]; J: joining gene segments; C: constant region gene. Gaps between contigs are indicated by a dotted black line, and the sequences from BAC are indicated by a bold line. (TIF) [file pone.0147704.s014.tif]

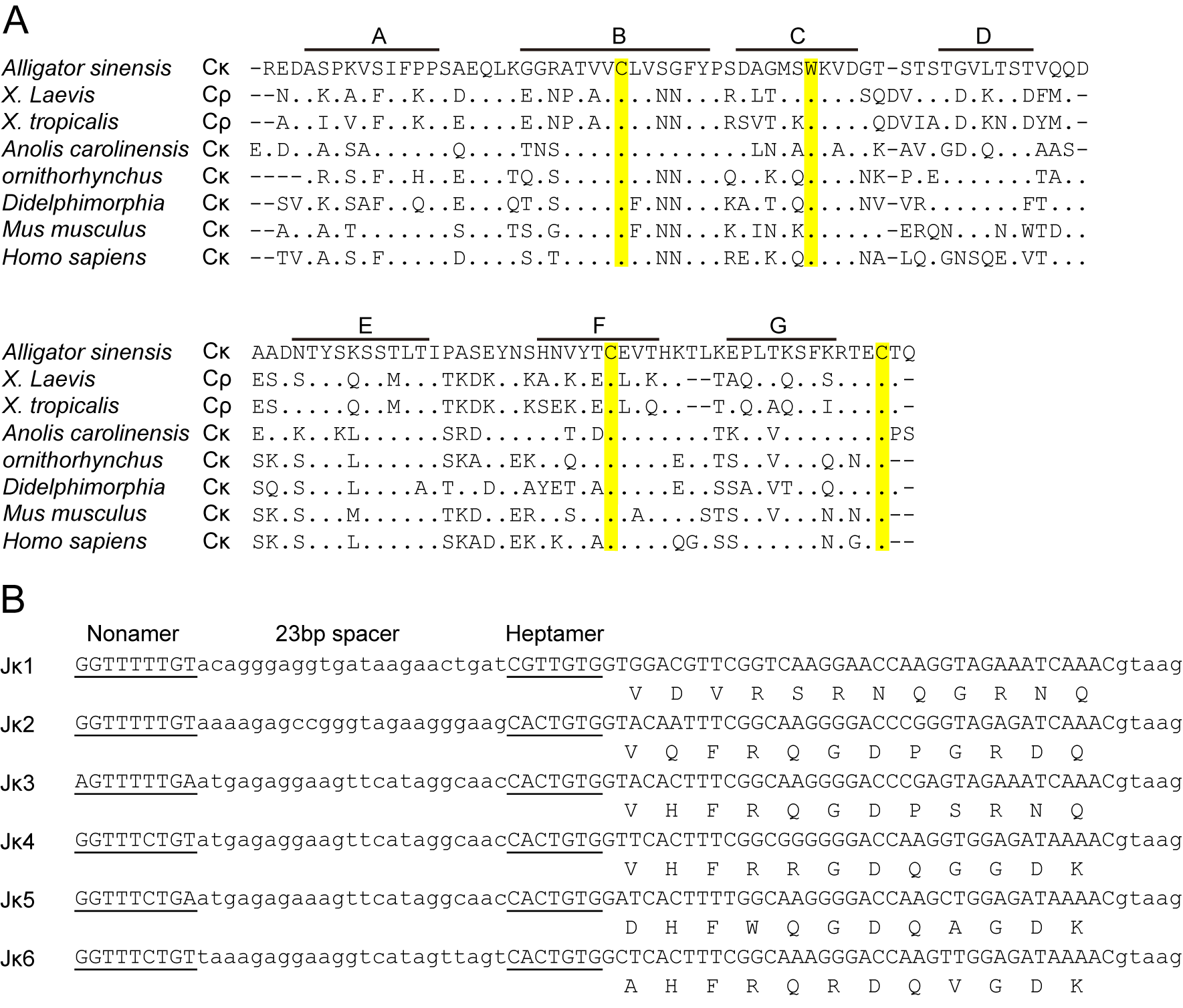

Supplement: S6 Fig — (A) Nucleotide and amino acid sequences of the six Alligator sinensis Jκ segments. (B) Sequence comparison of the Alligator sinensis Cκ genes with their counterparts in Homo sapiens, Mus musculus, Didelphimorphia, Ornithorhynchus, X. laevis, X. tropicalis and Anolis carolinensis. In the alignment, dots indicate identical amino acids and A-G over the lines represent the potential IgSF strands. The cysteine (C) and tryptophan (W) residues are shaded. (TIF) [file pone.0147704.s015.tif]

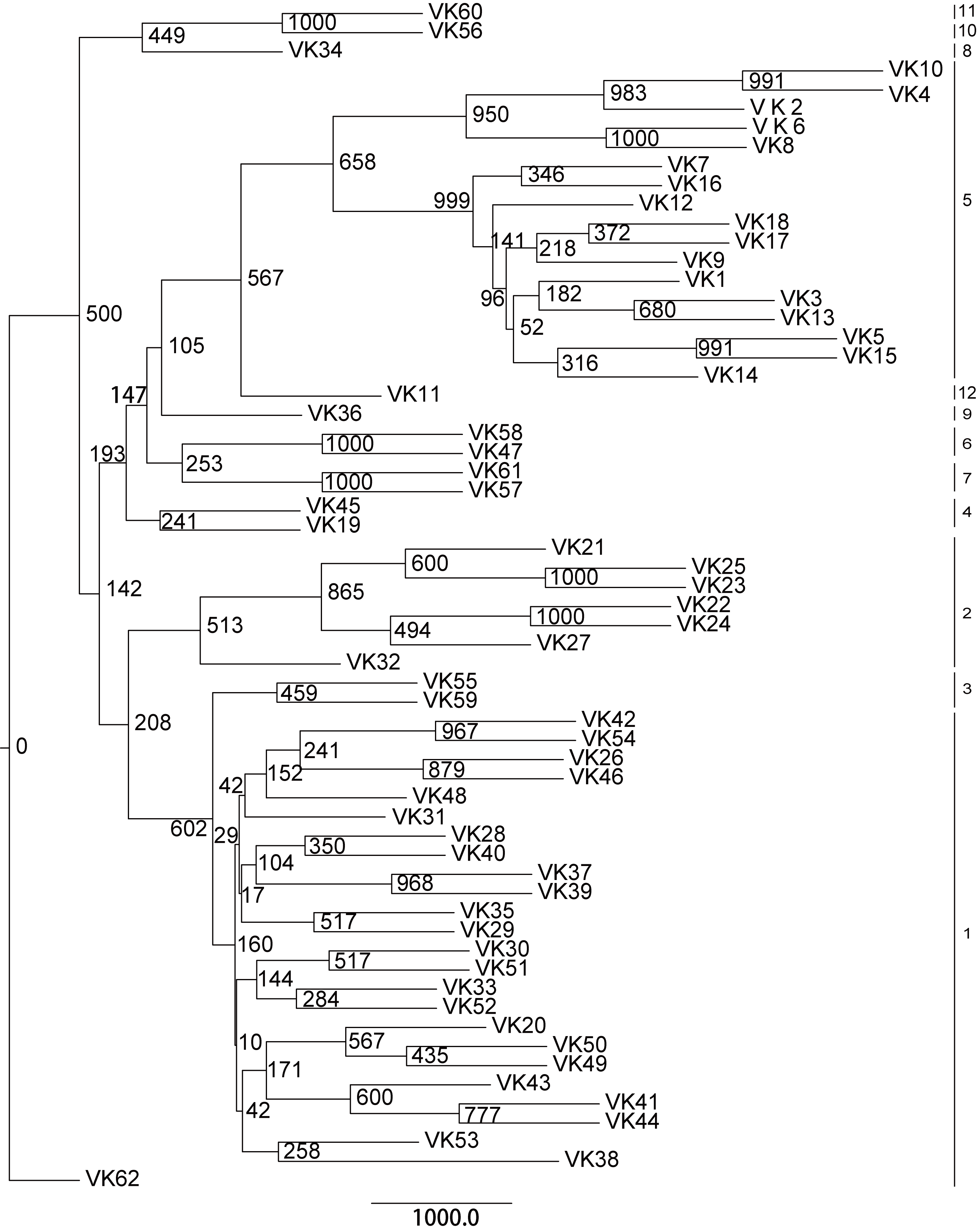

Supplement: S7 Fig — The phylogenetic tree was constructed using Phylip3.695 [60] and viewed in TREEVIEW [59]. (TIF) [file pone.0147704.s016.tif]

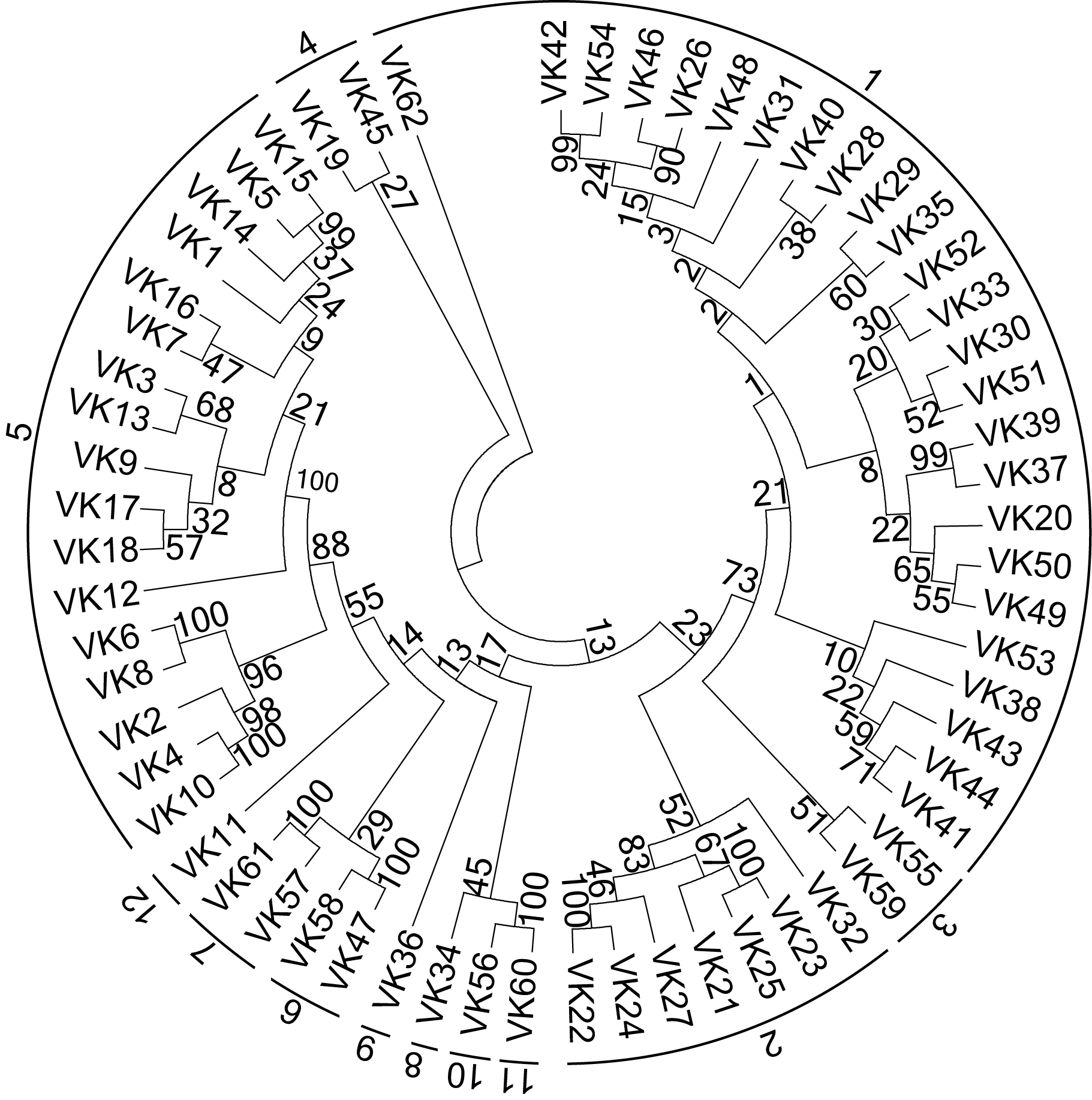

Supplement: S8 Fig — The tree is made by Neighbor-joining P-distance and pairwise deletions using MEGA6.0. (TIF) [file pone.0147704.s017.tif]

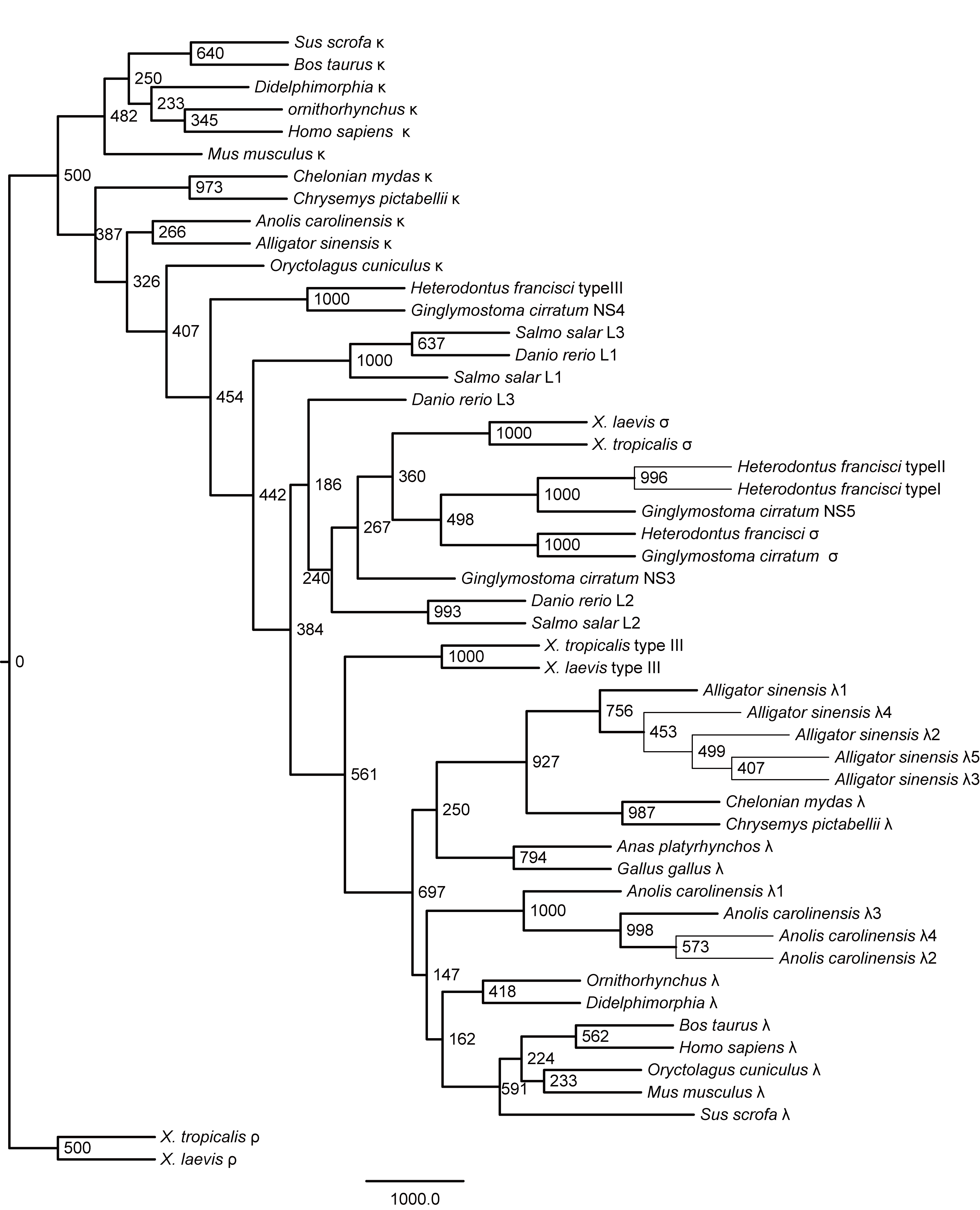

Supplement: S9 Fig — The phylogenetic tree was constructed using Phylip3.695 [60] and viewed in TREEVIEW [59]. (TIF) [file pone.0147704.s018.tif]

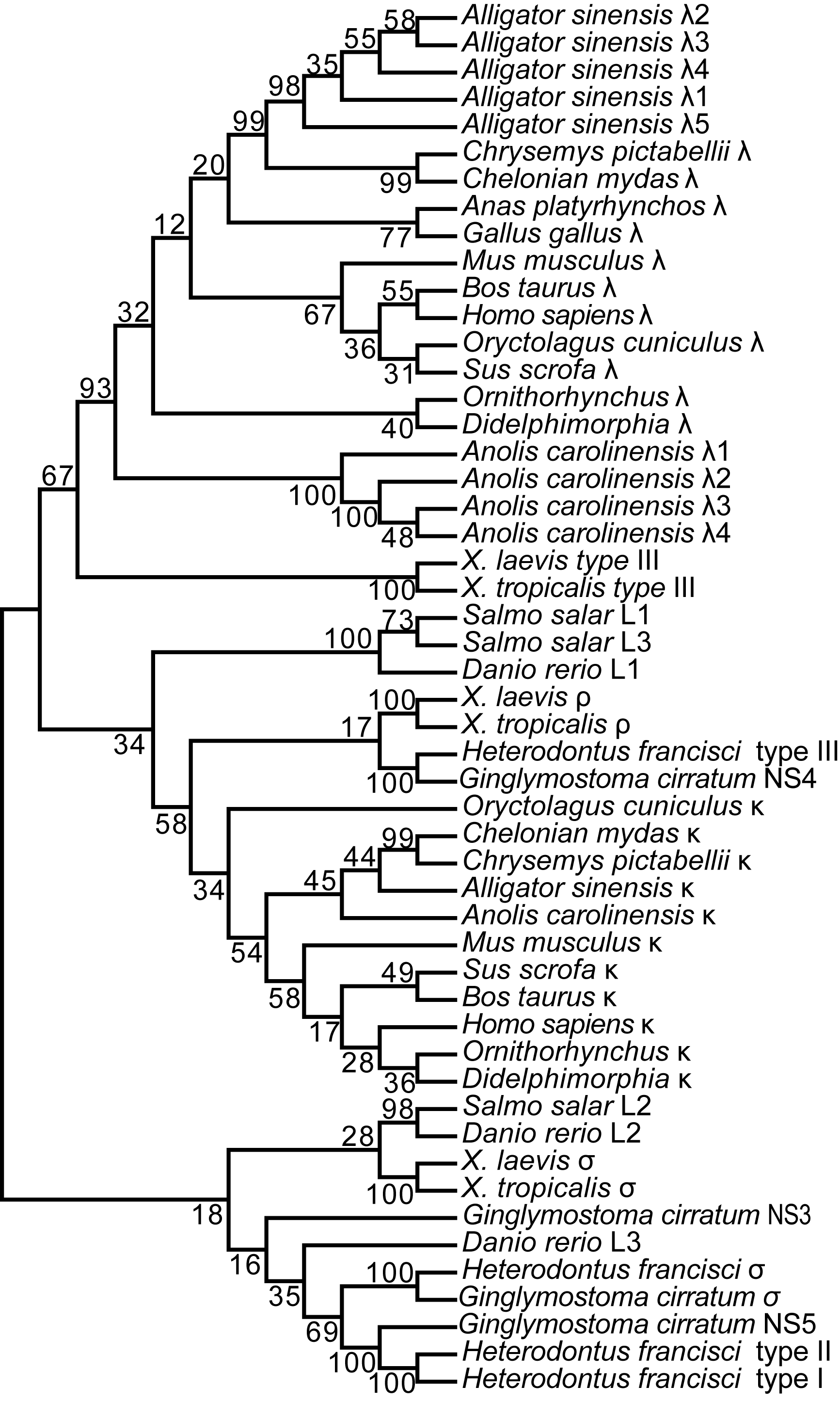

Supplement: S10 Fig — The phylogenetic tree was constructed using C domains, and by Neighbor-joining P-distance and pairwise deletions using MEGA6.0. (TIF) [file pone.0147704.s019.tif]

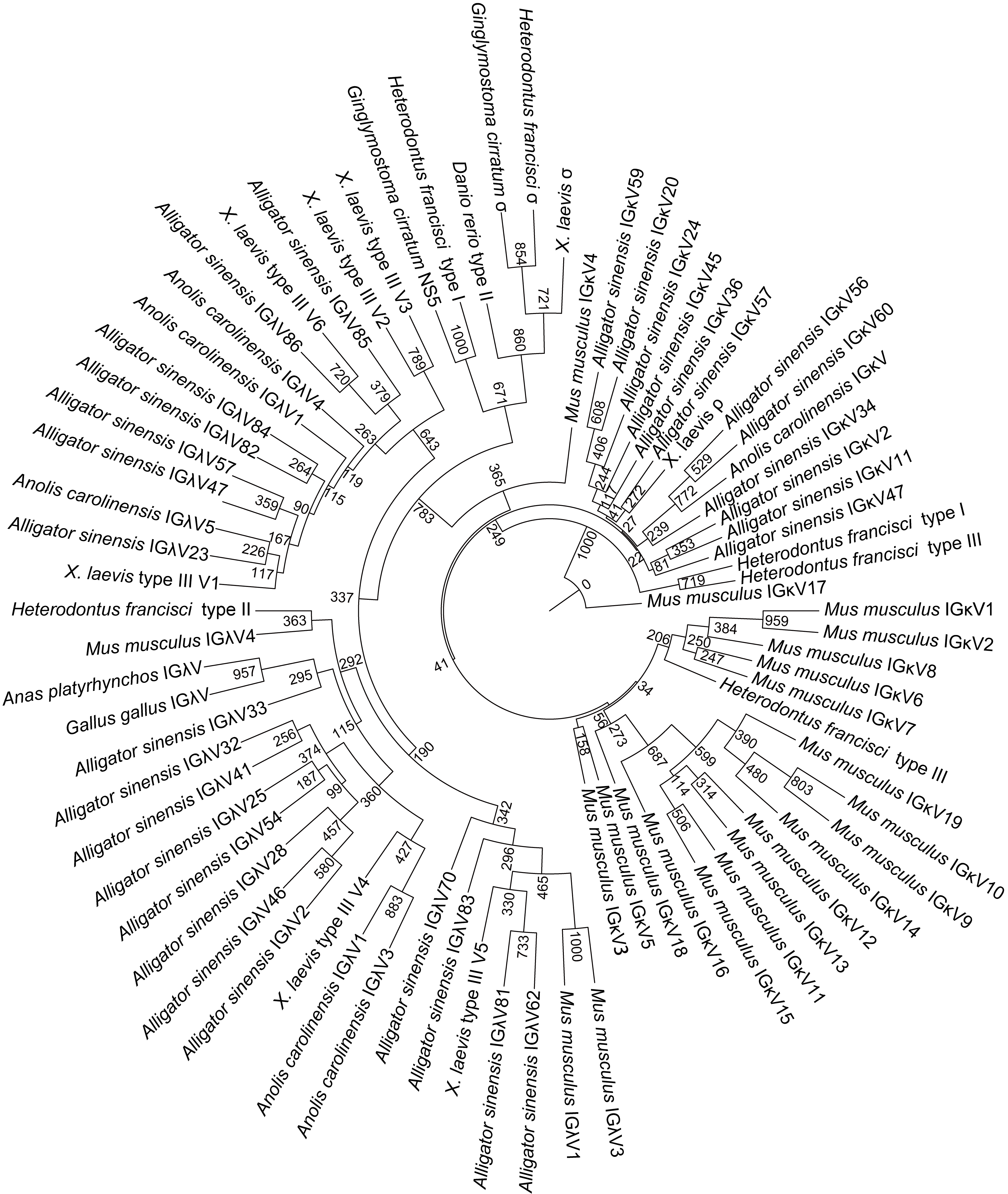

Supplement: S11 Fig — The phylogenetic tree was constructed using V domains. Each V subgroup is represented with one sequence per species chosen at random among the functional genes. The scale shown as a bar represents the genetic distance (number of nucleotide changes in the given scale). The credibility value for each node is shown. The phylogenetic tree was constructed using Phylip3.695 [60] and viewed in TREEVIEW [59]. (TIF) [file pone.0147704.s020.tif]

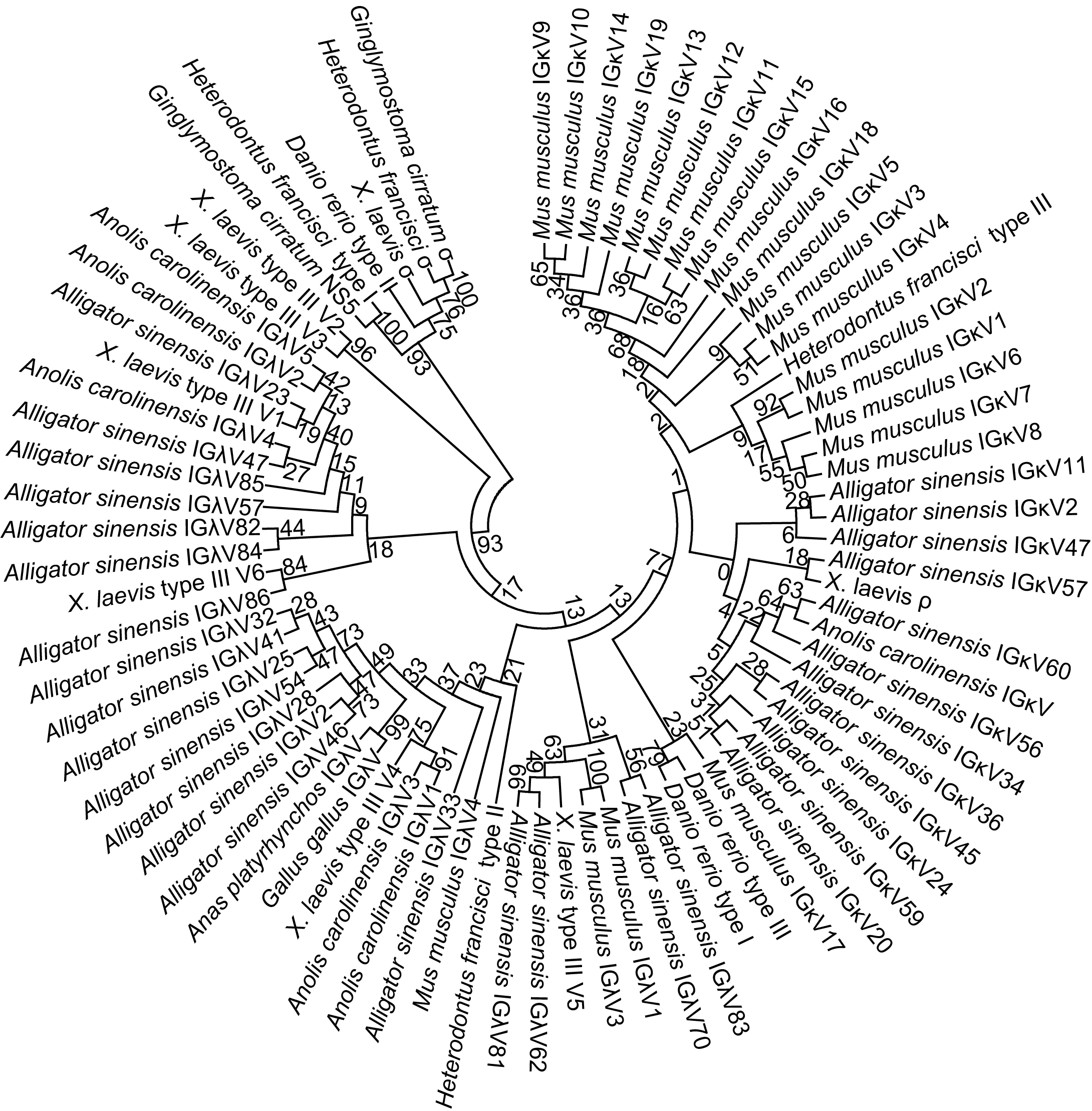

Supplement: S12 Fig — The phylogenetic tree was constructed using V domains, and by Neighbor-joining P-distance and pairwise deletions using MEGA6.0. (TIF) [file pone.0147704.s021.tif]
