# Supplementary material for: A Comprehensive Analysis of the Phylogeny, Genomic Organization and Expression of Immunoglobulin Light Chain Genes in Alligator sinensis, an Endangered Reptile Species
Source: PLoS One. 2016 Feb 22;11(2):e0147704. doi: 10.1371/journal.pone.0147704 (PMC4762898; doi:10.1371/journal.pone.0147704)
Supplement: S1 Table — (DOCX) [file pone.0147704.s022.docx]

**Table S1. Primers used for screening BACs**

| IgL gene | Location | Primer name | Primer sequence (5´→3´) | Product length (bp) | Positive BAC clones |
| --- | --- | --- | --- | --- | --- |
| Igκ | IgκC | IgLCkappa6U21 | GGCTTCTATCCAAGTGATGCG | 109 | Y329F14 |
|  |  | IgLCkappa114L21 | TGTACGTGTTGTCAGCGGCAT |  |  |
|  | End of Y329F14 | Y329F14F3 | GCATGGGAATGAATACACCAC | 317 | Y146B4 |
|  |  | Y329F14R3 | ACAGGGGGTTCCTCTTAGTCA |  |  |
|  | End of Y146B4 | Y146B4F | TGCTCTTCTCCCCAAAGCTA | 341 | Y77E6 |
|  |  | Y146B4R | ATAAGCAACAGGCTGCCATC |  |  |
|  | End of Y77E6 | Y77E6F2 | TCAGCCAGATCAGCTTCAGA | 394 | Y65C14 |
|  |  | Y77E6R2 | CCAAGCCAATGTGATGTGAC |  |  |
|  | End of Y65C14 | Y65C14F4 | TGTATCAGCTGTTTCCAGCACT | 597 | Y146M9 |
|  |  | Y65C14R4 | TCTGTGGGCAATAAGTTCAATG |  |  |
| Igλ | IgλC | IgLClambda6U21 | GGCCACGCTGGTGTGTCTGAT | 134 | Y127H24 |
|  |  | IgLClambda139L21 | CCATGTACTTGTTGTCGCTCT |  |  |
|  | End of Y127H24 | Y127H24-T7 263U21 | CACACTAATATCCCAAGGGAG | 317 | Y147P18 |
|  |  | Y127H24-T7 452L21 | GGGTGACACAGGCTCTTGCAC |  |  |
|  | End of Y147P18 | Y147P18F | CTTGTCTCTTGGGGAAGCAG | 324 | Y47P24 |
|  |  | Y147P18R | CGAAGCTCCTGTCAATCTCC |  |  |
|  | End of Y47P24 | Y47P24F3 | TTTGCATGAAGGGGCAGCT | 305 | Y210O3 |
|  |  | Y47P24R3 | TCCTGGAGACACAGACACTG |  |  |
