# Supplementary material for: A Comprehensive Analysis of the Phylogeny, Genomic Organization and Expression of Immunoglobulin Light Chain Genes in Alligator sinensis, an Endangered Reptile Species
Source: PLoS One. 2016 Feb 22;11(2):e0147704. doi: 10.1371/journal.pone.0147704 (PMC4762898; doi:10.1371/journal.pone.0147704)
Supplement: S2 Table — (DOCX) [file pone.0147704.s023.docx]

| Contig | Length (bp) | V_λ_ gene ^a^ | Partial V_λ_ | Pseudogene ^b^ | ORF | Total ^c^ | V_λ_ gene segments |
| --- | --- | --- | --- | --- | --- | --- | --- |
| KE698600.1 | 2063 | 1 | 0 | 0 | 0 | 1 | V_λ_38 |
| AVPB01102472.1 | 1781 | 0 | 0 | 1 | 0 | 1 | ΨV_λ_33 |
| KE698001.1 | 25665 | 0 | 0 | 2 | 0 | 2 | ΨV_λ_34 ΨV_λ_35 |
| KE698031.1 | 22920 | 0 | 0 | 4 | 0 | 4 | ΨV_λ_36 ΨV_λ_37 ΨV_λ_38 ΨV_λ_39 |
| KE697531.1 | 116501 | 12 | 0 | 7 | 0 | 19 | V_λ_39 V_λ_40 V_λ_41 V_λ_42 V_λ_43 V_λ_44 V_λ_45 V_λ_46 V_λ_47 V_λ_48 V_λ_49 V_λ_50 ΨV_λ_40 ΨV_λ_41 ΨV_λ_42 ΨV_λ_43 ΨV_λ_44 ΨV_λ_45 ΨV_λ_46 |
| KE697626.1 | 85990 | 5 | 1 | 7 | 0 | 13 | V_λ_51 V_λ_52 V_λ_53 V_λ_54 V_λ_55 ΨV_λ_47 ΨV_λ_48 ΨV_λ_49 ΨV_λ_50  ΨV_λ_51 ΨV_λ_52 ΨV_λ_53 V_λ_ partial 1 |
| KE695978.1 | 3621002 | 30 | 0 | 16 | 1 | 47 | V_λ_56 V_λ_57 V_λ_58 V_λ_59 V_λ_60 V_λ_61 V_λ_62 V_λ_63 V_λ_64 V_λ_65 V_λ_66 V_λ_67 V_λ_68 V_λ_69 V_λ_70 V_λ_71 V_λ_72 V_λ_73 V_λ_74 V_λ_75 V_λ_76 V_λ_77 V_λ_78 V_λ_79 V_λ_80 V_λ_81 V_λ_82 V_λ_83 V_λ_84 V_λ_85 V_λ_86 ΨV_λ_54 ΨV_λ_55 PV_λ_56 ΨV_λ_57 ΨV_λ_58 ΨV_λ_59 ΨV_λ_60 ΨV_λ_61 ΨV_λ_62 ΨV_λ_63 ΨV_λ_64 PV_λ_65 ΨV_λ_66 ΨV_λ_67 ΨV_λ_68 ΨV_λ_69 ORF2 |
| BAC Y127H24 | 93294 | 5 | 0 | 7 | 0 | 12 | V_λ_1 V_λ_2 V_λ_3 V_λ_4 V_λ_5 ΨV_λ_1 ΨV_λ_2 ΨV_λ_3 ΨV_λ_4 ΨV_λ_5 ΨV_λ_6 ΨV_λ_7 |
| BAC Y147P18 | 100680 | 8 | 0 | 7 | 0 | 15 | V_λ_6 V_λ_7 V_λ_8 V_λ_9 V_λ_10 V_λ_11 V_λ_12 V_λ_13 ΨV_λ_8 ΨV_λ_9 ΨV_λ_10 ΨV_λ_11 ΨV_λ_12 ΨV_λ_13 ΨV_λ_14 |
| BAC Y47P24 | 113265 | 12 | 0 | 10 | 1 | 23 | V_λ_14 V_λ_15 V_λ_16 V_λ_17 V_λ_18 V_λ_19 V_λ_20 V_λ_1 V_λ_22 V_λ_23 V_λ_24 V_λ_25 ΨV_λ_15 ΨV_λ_16 ΨV_λ_17 ΨV_λ_18 ΨV_λ_19 ΨV_λ_20 ΨV_λ_21 ΨV_λ_22 ΨV_λ_23 ΨV_λ_24 ORF1 |
| BAC Y213O3 | 88700 | 12 | 0 | 8 | 0 | 20 | V_λ_26 V_λ_27 V_λ_28 V_λ_29 V_λ_30 V_λ_31 V_λ_32 V_λ_33 V_λ_34 V_λ_35 V_λ_36 V_λ_37 ΨV_λ_25 ΨV_λ_26 ΨV_λ_27 ΨV_λ_28 ΨV_λ_29 ΨV_λ_30 ΨV_λ_31 ΨV_λ_32 |

**Table 1 Summary of the *Alligator sinensis* germline V_λ_ in contigs**

^a^ V_λ_ genes indicate the potentially functional V_λ_ genes

^b^ The pseudogenes that are contain either in – frame stop codons or lack of leading peptide.

^c^ Total number sums up the result from each of these contigs contain V_λ_.
