# Supplementary material for: A Comprehensive Analysis of the Phylogeny, Genomic Organization and Expression of Immunoglobulin Light Chain Genes in Alligator sinensis, an Endangered Reptile Species
Source: PLoS One. 2016 Feb 22;11(2):e0147704. doi: 10.1371/journal.pone.0147704 (PMC4762898; doi:10.1371/journal.pone.0147704)
Supplement: S3 Table — (DOCX) [file pone.0147704.s024.docx]

**Table 2 Summary of the *Alligator sinensis* germline V_κ_ in contigs.**

| Contig | Length (bp) | V_κ_ gene ^a^ | Partial V_κ_ | Pseudogene ^b^ | Total ^c^ | V_κ_ gene segments |
| --- | --- | --- | --- | --- | --- | --- |
| AVPB01043985.1 | 2345 | 1 | 0 | 0 | 1 | V_κ_43 |
| KE697554.1 | 105813 | 5 | 0 | 8 | 13 | V_κ_38 V_κ_39 V_κ_40 V_κ_41 V_κ_42 ΨV_κ_30 ΨV_κ_31 ΨV_κ_32 ΨV_κ_33 ΨV_κ_34 ΨV_κ_35 ΨV_κ_36 ΨV_κ_37 |
| KE698055.1 | 20612 | 1 | 0 | 0 | 1 | V_κ_44 |
| KE698335.1 | 7712 | 0 | 0 | 1 | 1 | ΨV_κ_38 |
| KE698081.1 | 18319 | 0 | 1 | 1 | 2 | V_κ_ partial 2 ΨV_κ_39 |
| KE698149.1 | 15488 | 0 | 0 | 2 | 2 | ΨV_κ_40 ΨV_κ_41 |
| AVPB01053098.1 | 13945 | 1 | 0 | 2 | 3 | V_κ_45 ΨV_κ_42 ΨV_κ_43 |
| KE698098.1 | 17453 | 0 | 0 | 2 | 2 | ΨV_κ_44 ΨV_κ_45 |
| AVPB01130521.1 | 2996 | 1 | 0 | 0 | 1 | V_κ_46 |
| KE697644.1 | 80977 | 1 | 1 | 1 | 3 | V_κ_47 ΨV_κ_46 V_κ_ partial 3 |
| KE698428.1 | 5642 | 1 | 0 | 0 | 1 | V_κ_48 |
| KE698356.1 | 7053 | 1 | 0 | 0 | 1 | V_κ_49 |
| KE698585.1 | 2398 | 1 | 0 | 0 | 1 | V_κ_50 |
| AVPB01143799.1 | 4208 | 1 | 0 | 0 | 1 | V_κ_51 |
| KE698008.1 | 25074 | 0 | 0 | 3 | 3 | ΨV_κ_47 ΨV_κ_48 ΨV_κ_49 |
| AVPB01013186.1 | 4343 | 0 | 0 | 1 | 1 | ΨV_κ_50 |
| KE698096.1 | 17506 | 2 | 0 | 0 | 2 | V_κ_52 V_κ_53 |
| KE695928.1 | 4398749 | 9 | 0 | 6 | 15 | V_κ_54 V_κ_55 V_κ_56 V_κ_57 V_κ_58 V_κ_59 V_κ_60 V_κ_61 V_κ_62 ΨV_κ_51 ΨV_κ_52 ΨV_κ_53 ΨV_κ_54 ΨV_κ_55 ΨV_κ_56 |
| BAC Y329F14 | 107661 | 0 | 0 | 1 | 1 | ΨV_κ_1 |
| BAC Y146B4 | 117481 | 7 | 0 | 6 | 13 | V_κ_1 V_κ_2 V_κ_3 V_κ_4 V_κ_5 V_κ_6 V_κ_7 ΨV_κ_2 ΨV_κ_3 ΨV_κ_4 ΨV_κ_5 ΨV_κ_6 ΨV_κ_7 |

| Contig | Length (bp) | V_κ_ gene ^a^ | Partial V_κ_ | Pseudogene ^b^ | Total ^c^ | V_κ_ gene segments |
| --- | --- | --- | --- | --- | --- | --- |
| BAC Y77E6 | 100267 | 10 | 0 | 5 | 15 | V_κ_16 V_κ_17 V_κ_18 V_κ_19 V_κ_20 V_κ_21 V_κ_22 V_κ_23 V_κ_24 V_κ_25 ΨV_κ_16 ΨV_κ_17 ΨV_κ_18 ΨV_κ_19 ΨV_κ_20 |
| BAC Y65C14 | 105355 | 12 | 0 | 9 | 21 | V_κ_25 V_κ_26 V_κ_27 V_κ_28 V_κ_29 V_κ_30 V_κ_31 V_κ_32 V_κ_33 V_κ_34 V_κ_35 V_κ_36 V_κ_37 ΨV_κ_21 ΨV_κ_22 ΨV_κ_23 ΨV_κ_24 ΨV_κ_25 ΨV_κ_26 ΨV_κ_27 ΨV_κ_28 ΨV_κ_29 |
| BAC Y146M19 | 105355 | 12 | 0 | 9 | 21 | V_κ_25 V_κ_26 V_κ_27 V_κ_28 V_κ_29 V_κ_30 V_κ_31 V_κ_32 V_κ_33 V_κ_34 V_κ_35 V_κ_36 V_κ_37 ΨV_κ_21 ΨV_κ_22 ΨV_κ_23 ΨV_κ_24 ΨV_κ_25 ΨV_κ_26 ΨV_κ_27 ΨV_κ_28 ΨV_κ_29 |

**Table 2 Summary of the Chinese alligator germline Vκ in contigs (continued)**.

^a^ V_κ_ genes indicate the potentially functional V_κ_ genes.

^b^ The pseudogenes that are contain either in – frame stop codons or lack of leading peptide.

^c^ Total number sums up the result from each of these contigs contain V_κ_.
